# Interactions between the lipidome and genetic and environmental factors in autism

**DOI:** 10.1038/s41591-023-02271-1

**Published:** 2023-04-19

**Authors:** Chloe X. Yap, Anjali K. Henders, Gail A. Alvares, Corey Giles, Kevin Huynh, Anh Nguyen, Leanne Wallace, Tiana McLaren, Yuanhao Yang, Leanna M. Hernandez, Michael J. Gandal, Narelle K. Hansell, Dominique Cleary, Rachel Grove, Claire Hafekost, Alexis Harun, Helen Holdsworth, Rachel Jellett, Feroza Khan, Lauren P. Lawson, Jodie Leslie, Mira Levis Frenk, Anne Masi, Nisha E. Mathew, Melanie Muniandy, Michaela Nothard, Jessica L. Miller, Lorelle Nunn, Lachlan T. Strike, Gemma Cadby, Eric K. Moses, The Busselton Health Study Investigators, Joseph Hung, Jennie Hui, John Beilby, Greig I. de Zubicaray, Paul M. Thompson, Katie L. McMahon, Margaret J. Wright, Peter M. Visscher, Paul A. Dawson, Cheryl Dissanayake, Valsamma Eapen, Helen S. Heussler, Andrew J. O. Whitehouse, Peter J. Meikle, Naomi R. Wray, Jacob Gratten

**Affiliations:** 1 Mater Research Institute, The University of Queensland, Brisbane, Queensland Australia; 2 Institute for Molecular Bioscience, The University of Queensland, Brisbane, Queensland Australia; 3 Cooperative Research Centre for Living with Autism, Long Pocket, Queensland Australia; 4 Telethon Kids Institute, The University of Western Australia, Perth, Western Australia Australia; 5 Baker Heart and Diabetes Institute, Melbourne, Victoria Australia; 6 Baker Department of Cardiometabolic Health, The University of Melbourne, Melbourne, Victoria Australia; 7 Department of Psychiatry and Biobehavioral Sciences, Semel Institute, David Geffen School of Medicine, University of California Los Angeles, Los Angeles, CA USA; 8 Lifespan Brain Institute at Penn Medicine and The Children’s Hospital of Philadelphia, Department of Psychiatry, University of Pennsylvania, Philadelphia, PA USA; 9 Program in Neurobehavioral Genetics, Semel Institute, David Geffen School of Medicine, University of California Los Angeles, Los Angeles, CA USA; 10 Department of Human Genetics, David Geffen School of Medicine, University of California Los Angeles, Los Angeles, CA USA; 11 Queensland Brain Institute, The University of Queensland, Brisbane, Queensland Australia; 12 Faculty of Health, University of Technology Sydney, Sydney, New South Wales Australia; 13 School of Psychiatry, Faculty of Medicine, University of New South Wales, Sydney, New South Wales Australia; 14 Child Health Research Centre, The University of Queensland, Brisbane, Queensland Australia; 15 Olga Tennison Autism Research Centre, La Trobe University, Melbourne, Victoria Australia; 16 Department of Psychology, Counselling and Therapy, La Trobe University, Melbourne, Victoria Australia; 17 School of Population and Global Health, The University of Western Australia, Perth, Western Australia Australia; 18 Menzies Institute for Medical Research, University of Tasmania, Hobart, Tasmania Australia; 19 School of Biomedical Sciences, The University of Western Australia, Perth, Western Australia Australia; 20 School of Psychology and Counselling, Faculty of Health, Queensland University of Technology, Brisbane, Queensland Australia; 21 Imaging Genetics Center, Mark and Mary Stevens Neuroimaging and Informatics Institute, Keck School of Medicine, University of Southern California, Los Angeles, CA USA; 22 School of Clinical Sciences, Centre for Biomedical Technologies, Queensland University of Technology, Brisbane, Queensland Australia; 23 Centre for Advanced Imaging, The University of Queensland, Brisbane, Queensland Australia; 24 Academic Unit of Child Psychiatry South West Sydney, Ingham Institute for Applied Medical Research, Liverpool Hospital, Sydney, New South Wales Australia; 25 Child Development Program, Children’s Health Queensland, Brisbane, Queensland Australia; 26 Baker Department of Cardiovascular Research, Translation and Implementation, La Trobe University, Melbourne, Victoria Australia; 27 The Busselton Population Medical Research Institute Inc., Perth, Australia

**Keywords:** Lipidomics, Data integration, Genomics, Biomarkers, Autism spectrum disorders

## Abstract

Autism omics research has historically been reductionist and diagnosis centric, with little attention paid to common co-occurring conditions (for example, sleep and feeding disorders) and the complex interplay between molecular profiles and neurodevelopment, genetics, environmental factors and health. Here we explored the plasma lipidome (783 lipid species) in 765 children (485 diagnosed with autism spectrum disorder (ASD)) within the Australian Autism Biobank. We identified lipids associated with ASD diagnosis (*n* = 8), sleep disturbances (*n* = 20) and cognitive function (*n* = 8) and found that long-chain polyunsaturated fatty acids may causally contribute to sleep disturbances mediated by the *FADS* gene cluster. We explored the interplay of environmental factors with neurodevelopment and the lipidome, finding that sleep disturbances and unhealthy diet have a convergent lipidome profile (with potential mediation by the microbiome) that is also independently associated with poorer adaptive function. In contrast, ASD lipidome differences were accounted for by dietary differences and sleep disturbances. We identified a large chr19p13.2 copy number variant genetic deletion spanning the *LDLR* gene and two high-confidence ASD genes (*ELAVL3* and *SMARCA4*) in one child with an ASD diagnosis and widespread low-density lipoprotein-related lipidome derangements. Lipidomics captures the complexity of neurodevelopment, as well as the biological effects of conditions that commonly affect quality of life among autistic people.

## Abstract (web summary)

Detailed analyses pairing deep phenotyping and molecular profiles reveal mediating roles of plasma lipids in genetic and environmental factors associated with autism-related traits in children.

## Main

Autism spectrum disorder (ASD) is a neurodevelopmental condition characterized by social and communication difficulties, restricted and repetitive behaviors and differences in sensory sensitivity. ASD commonly co-occurs with other medical and psychiatric conditions, including sleep disorders, feeding disorders, gastrointestinal complaints, anxiety and seizures^1^. Autistic people are greatly interested in these co-occurring conditions as they adversely impact on development, quality of life and long-term health and wellbeing^2^^,^^3^. However, the biological interactions between ASD and co-occurring conditions are understudied. Emerging high-throughput omics technologies that assay molecular traits (for example, RNA transcripts, proteins and lipids) may help to improve biological understanding and identify novel biomarkers to improve the detection of ASD and commonly co-occurring conditions.

Rare variation in lipid metabolism genes (for example, *EFR3A*^4^) is associated with nonsyndromic idiopathic ASD, and genetic syndromes of lipid metabolism are frequently associated with neurodevelopmental delay^5^ (for example, Smith–Lemli–Opitz syndrome, Niemann–Pick syndrome and Tay–Sachs syndrome). Outside of these rare genetic diagnoses, others have investigated relationships between clinical lipids and ASD diagnosis, with mixed results. Smaller studies have identified potential associations between hypocholesterolemia and ASD diagnosis^6–8^, whereas a large study integrating genetic and electronic health record data suggested that there may be a dyslipidemia subtype of ASD^9^. Indeed, autistic people may be at a higher risk of treatable cardiometabolic disease^10^.

Co-occurring conditions could predispose autistic people to altered lipid and metabolic profiles. For example, ASD-associated restricted interests and sensory sensitivities predispose to specific dietary preferences^11,12^, in turn impacting metabolic health and development. Furthermore, children diagnosed with ASD commonly have sleep disruption^13^, partly mediated by shared genetic predisposition^14,15^. In the general pediatric population, longitudinal studies have found that difficulty at mealtimes^16^ and sleep disorders^16,17^ in early childhood are associated with overweight or obesity in childhood, and sleep disturbances may predispose to clinical lipid derangement^18,19^, although these reports are not yet conclusive^20^. Specifically for ASD, relationships between the wider lipidome and sleep disruption have not been investigated and it is unclear whether dietary patterns are a confounder.

Despite interest in the ASD lipidome spanning almost 20 years, there are limited well-powered and appropriately designed studies accompanied by extensive metadata (for example, genetic, dietary, sleep, medication, demographic and psychometric data) to disentangle potential mediators of ASD–lipid relationships. In this article, we investigate relationships between the plasma lipidome (783 species) and autism-associated traits, using rich phenotypic and biological data from participants in the Australian Autism Biobank (AAB) and Queensland Twin Adolescent Brain (QTAB) Project. We looked for associations between autism-related traits (ASD diagnosis, cognitive function and sleep disruption) and various aspects of the lipidome. We also integrated lipidomics with genetic and environmental data (diet, the microbiome and medications).

## Results

### Overview of the dataset

**Fig. 1:**
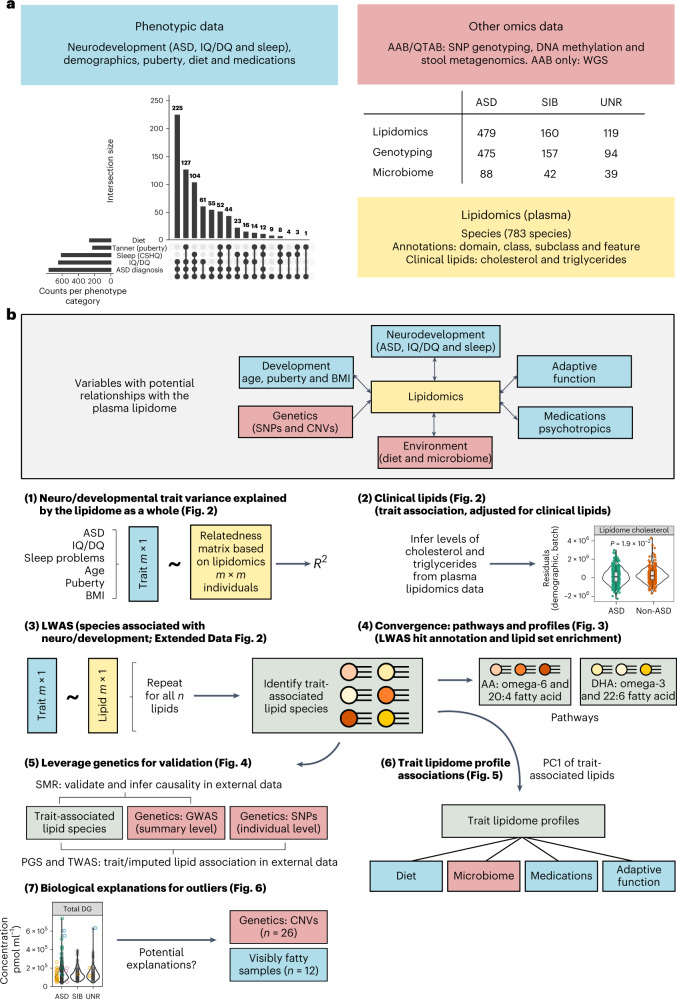
Overview of data and analysis. **a**, Phenotypic data and multi-omics data that were used in this analysis. **b**, Outline of the methods. Blue boxes correspond to phenotypic data, yellow boxes correspond to lipidomics-based data and red boxes correspond to other omics data. AA, arachidonic acid; WGS, whole-genome sequencing.

**Extended Data Fig. 1:**
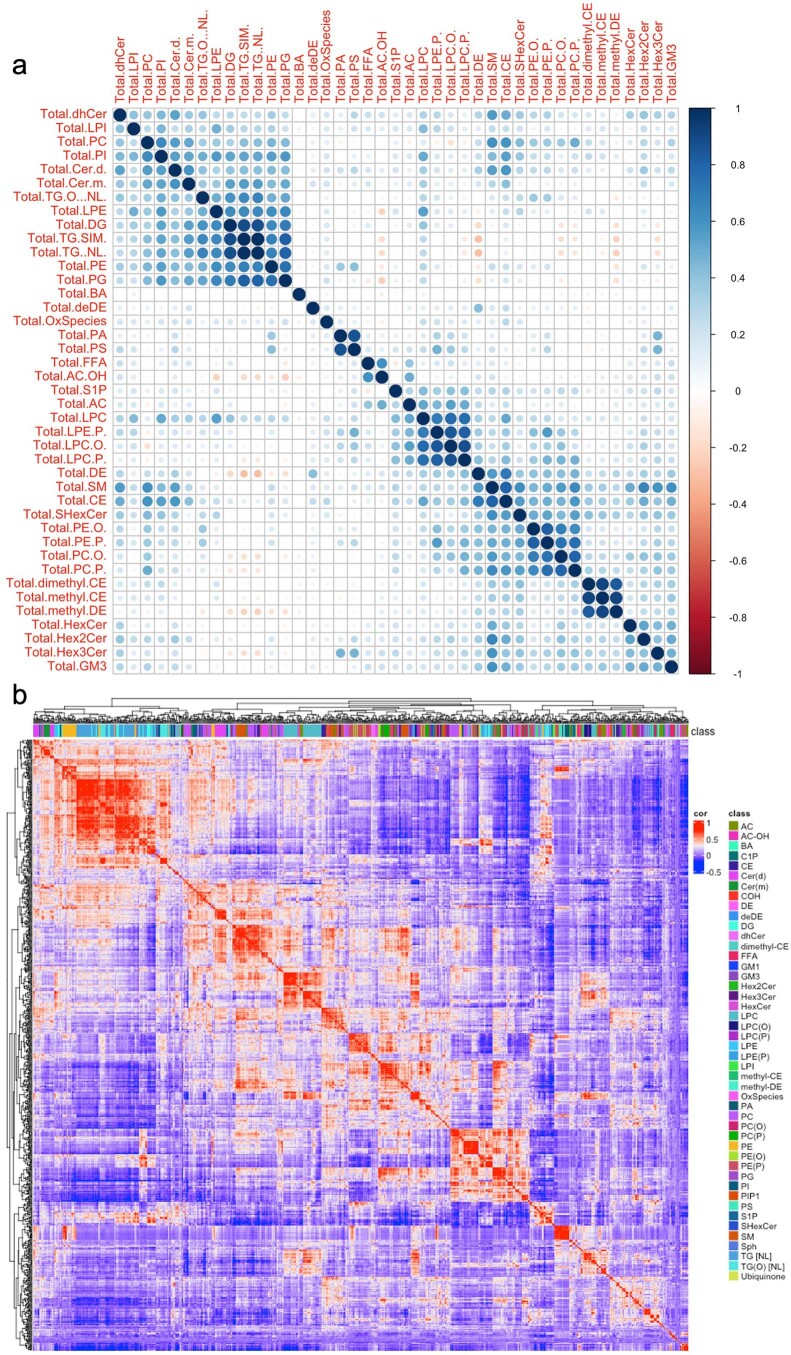
Lipidome data correlation structures. Correlation (Pearson’s r) between **a**) lipid classes and **b**) lipid species (grouped by class).

The AAB combines deep biological and phenotypic data collected from children diagnosed with ASD, siblings without a diagnosis (SIB) and unrelated children without a diagnosis (UNR) (Fig. 1a and Supplementary Table 1). We profiled the plasma lipidome (783 species after quality control; Methods and Extended Data Fig. 1) in 765 children (*n* = 485 ASD, 160 SIB and 120 UNR, with 500 boys and 265 girls, including 24 UNR participants from the QTAB Project) at the species level (the most granular classification) and class level (which species collapse into) within the lipid ontology^21^; these can be further annotated by subclass, feature and domain (Supplementary Table 2). We identified several outlier groups (*n* = 7 statistical outliers, *n* = 64 storage duration outliers (all in the ASD group; Supplementary Figs. 1 and 2) and *n* = 12 visibly fatty samples). Depending on the analysis, some of these outlier groups were retained (Methods).

We considered three key neurodevelopmental phenotypes: ASD diagnosis, intelligence quotient/developmental quotient composite score (IQ/DQ; Methods) and sleep disturbances (measured using the Children’s Sleep Habits Questionnaire (CSHQ) total score) as the latter two are associated with ASD.

We assessed relationships between neurodevelopmental phenotypes and covariates with potential lipidome effects: demographics, batch, diet and medications. Age was well matched between ASD, SIB and UNR groups, although children with higher IQ/DQ tended to be slightly older (linear model; *b* = 0.018; s.e. = 0.007; *P* = 5.9 × 10^−3^). Male sex was associated with ASD diagnosis (chi-squared test; *P* = 8.7 × 10^−10^) and lower IQ/DQ (linear model (IQ/DQ ~ sex); *b* = 6.6; s.e. = 1.9; *P* = 4.6 × 10^−4^). For batch variables, neurodevelopmental phenotypes were associated with sample storage duration but not injection batch or collection time of day, informing our approach to outliers (Methods and Supplementary Figs. 1 and 2). Reduced meat intake was associated with ASD diagnosis (dietary principal component 3 (PC3); see Methods) (*b* = −0.45; s.e. = 0.16; *P* = 5.8 × 10^−3^) and lower IQ/DQ scores (*b* = 2.4; s.e. = 1.1; *P* = 2.5 × 10^−2^), both adjusted for age and sex. For medication classes, ASD diagnosis was associated with attention deficit hyperactivity disorder (ADHD)/behavioral medications (odds ratio (OR) = 5.6; s.e. = 0.4; *P* = 7.9 × 10^−5^), antipsychotics (OR = 10.5; s.e. = 1.0; *P* = 2.2 × 10^−2^), anxiolytics/antidepressants (OR = 19.4; s.e. = 1.1; *P* = 3.6 × 10^−3^), sleep medications (OR = 13.4; s.e. = 0.6; *P* = 1.3 × 10^−5^) and fish oil/docosahexaenoic acid (DHA) supplements (OR = 7.39; s.e. = 0.34; *P* = 4.2 × 10^−9^). Lower IQ/DQ was associated with antiepileptic drug use (*b* = −18.1; s.e. = 6.6; *P* = 6.6 × 10^−3^), sleep medications (*b* = −8.8; s.e. = 3.2; *P* = 6.5 × 10^−3^) and fish oil/DHA supplements (*b* = −4.9; s.e. = 2.5; *P* = 5.0 × 10^−2^). Sleep disturbances were associated with ADHD/behavioral medications (*b* = 2.7; s.e. = 1.3; *P* = 4.2 × 10^−2^) and sleep medications (*b* = 7.4; s.e. = 1.2; *P* = 3.6 × 10^−9^).

### Associations between ASD diagnosis and inferred clinical lipids

**Fig. 2:**
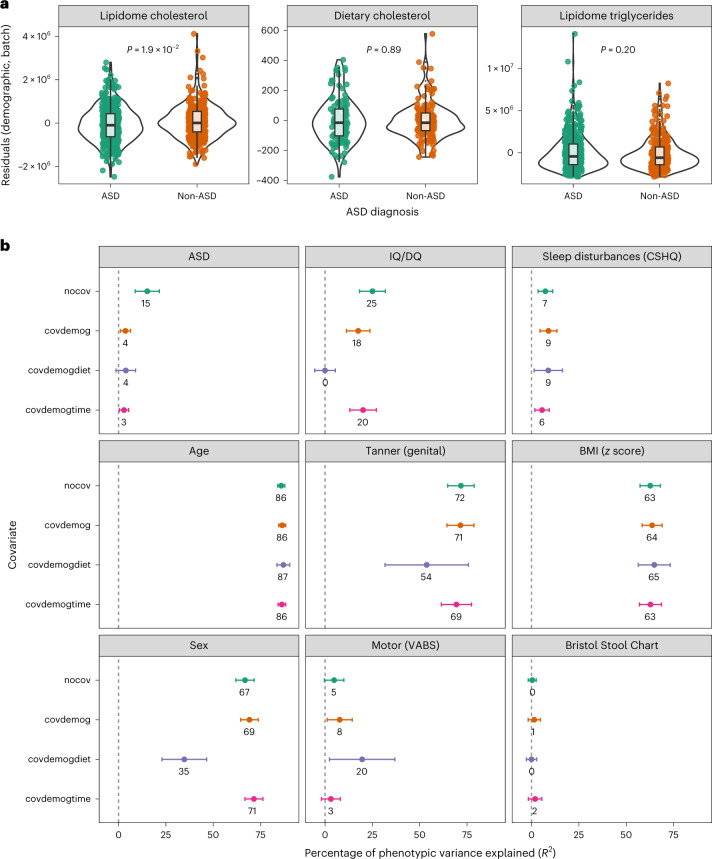
Associations with inferred clinical lipids and variance component analysis. **a**, Differences in residuals for inferred clinical lipids (cholesterol and triglycerides) and dietary cholesterol (after regressing out demographic and batch variables: age, age^2^, sex, batch, injection order and storage time) between ASD and non-ASD groups (*n* = 694). The box plots show median values and quartiles of the distribution. Statistical significance was determined by logistic regression (ASD diagnosis ~ age + sex + batch + injection order + clinical lipid). The *P* values are unadjusted for multiple testing (Methods). **b**, Percentage of trait variance associated with the overall lipidome. The error bars represent s.e. Sensitivity analyses were performed for the following covariate combinations: nocov (no covariates); covdemog (demographic, batch and storage duration); covdemogdiet (covdemog and dietary PC1–PC3; this analysis had the smallest subset of individuals with complete data and hence wider CIs); and covdemogtime (covdemog and collection time of day). For the covdemog analysis (that is, the primary analysis), the sample sizes were as follows: *n* = 694 (ASD), *n* = 642 (IQ/DQ), *n* = 611 (sleep disturbances), *n* = 758 (age), *n* = 224 (Tanner (genital) score), *n* = 715 (BMI), *n* = 758 (sex), *n* = 217 (motor (VABS)) and *n* = 258 (Bristol Stool Chart).

In the absence of clinical lipid measurements, we inferred total plasma cholesterol and triglycerides from lipidome data (Methods). ASD diagnosis (*n* = 694, excluding storage duration outliers) was modestly associated with decreased cholesterol levels (OR = 0.82 per s.d.; 95% confidence interval (CI) = 0.70–0.97; *P* = 1.9 × 10^−2^; Fig. 2a, Supplementary Fig. 3 and Methods) independent of dietary cholesterol, antipsychotics, ADHD/behavioral medications and fish oil/DHA intake (Methods). In contrast with ASD diagnosis, there were no associations between IQ/DQ (*n* = 642) or sleep disturbances (*n* = 607) and either inferred total cholesterol or triglycerides. As expected, body mass index (BMI) was associated with increased dietary cholesterol (*b* = 1.4 × 10^−3^; s.e. = 0.6 × 10^−3^; *P* = 2.3 × 10^−2^), inferred lipidome cholesterol (*b* = 1.3 × 10^−7^; s.e. = 0.3 × 10^−8^; *P* = 2.0 × 10^−7^) and triglycerides (*b* = 1.7 × 10^−7^; s.e. = 0.6 × 10^−7^; *P* = 8.0 × 10^−3^).

### Trait variance associated with the lipidome

We investigated the lipidome’s overall association with neurodevelopmental and anthropomorphic traits under an additive model using omics data-based restricted maximum likelihood (OREML)^22^ analysis (Fig. 2 and Supplementary Table 3). As primary analyses, we adjusted for demographics and batch effects (Fig. 2b, “covdemog”) to maximize sample size.

The lipidome explained false discovery rate (FDR)-significant (*q* < 0.05) variance in age (coefficient of determination (*R*^2^) = 86.4%; s.e. = 1.8%; *P* = ~0; *n* = 758), Tanner score for pubertal stage (*R*^2^ = 71.5%; s.e. = 7.1%; *P* = 5.6 × 10^−17^; *n* = 224), sex (*R*^2^ = 69.1%; s.e. = 4.6%; *P* = ~0; *n* = 758) and BMI (*R*^2^ = 63.7%; s.e. = 5.3%; *P* = ~0; *n* = 715) (Fig. 2b), as well as dietary traits (Supplementary Fig. 4).

Lipidome associations with the following neurodevelopmental traits were more modest (albeit still FDR significant; Fig. 2b): IQ/DQ (*R*^2^ = 17.5%; s.e. = 6.2%; *P* = 2.8 × 10^−6^; *n* = 642), sleep disturbances (*R*^2^ = 9.0%; s.e. = 4.5%; *P* = 4.1 × 10^−5^; *n* = 607) and ASD diagnosis (*R*^2^ = 3.6%; s.e. = 2.7%; *P* = 4.7 × 10^−3^; *n* = 694), after excluding storage outliers. There was also a nominally significant association with the adaptive motor domain score of the second edition of the Vineland Adaptive Behaviour Scale (VABS-II) despite a small sample size (*R*^2^ = 8%; s.e. = 7%; *P* = 4.7 × 10^−2^; *n* = 217). The lipidome was not associated with stool consistency (*R*^2^ = 1.5%; s.e. = 3.2%; *P* = 0.28; *n* = 255).

The *R*^2^ estimates were broadly consistent in sensitivity analyses excluding covariates, adjusting for diet in a smaller subset of participants and adjusting for collection time of day (Fig. 2b and Methods), with two exceptions: analysis of ASD without covariates (“nocov”), probably reflecting residual confounding from storage duration, and analysis of sex adjusted for diet (“covdemogdiet”), which may reflect that the ASD group had more male participants and dietary differences (Methods).

### Lipidome-wide association studies

**Extended Data Fig. 2:**
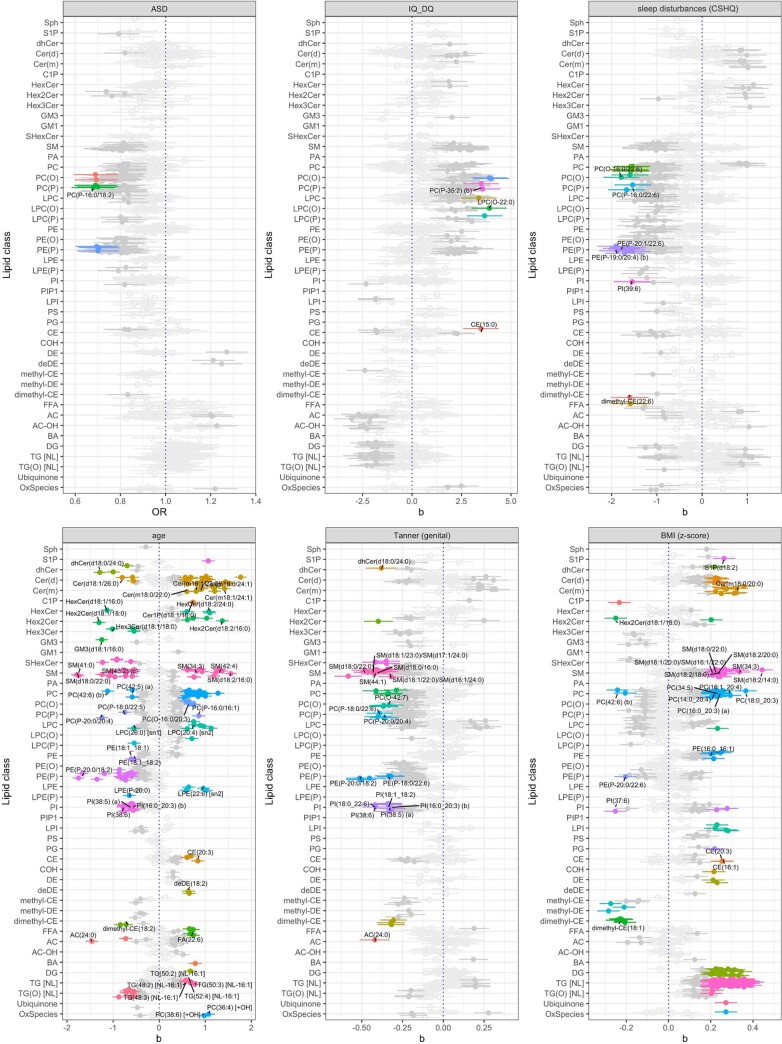
Lipidome-wide association study (LWAS) forest plots at the lipid species level. Plots shown for ASD, IQ/DQ, sleep disturbances (CSHQ total score), age, Tanner score (genital), and BMI (z-score). Point denotes effect estimate and error bars denote standard error. All analyses included covariates of injection order and storage time; the ASD, IQ/DQ and sleep disturbances LWAS also included age, age2 and sex as covariates; the age and Tanner score (genital) LWAS also included sex; the BMI (z-score) LWAS did not include additional covariates as these are accounted for within the population-normed z-score. The ASD analysis excluded storage time outliers. Each point denotes a lipid species, grouped by rows as lipid classes. Lighter-grey open points represent species with association p > 0.05, darker-grey filled points represent species with association p < =0.05. Colour denotes species passing multiple testing correction (dividing by the effective number of independent lipids; see Methods). Text denotes lipid species retained in a backwards stepwise regression model with covariates, representing the effective number of independent LWAS hits.

Next, we performed lipidome-wide association studies (LWASs) to test for associations between individual lipids and six traits (ASD, IQ/DQ, sleep disturbances, age, Tanner stage and BMI) with significant lipidome associations in the variance component analyses (Extended Data Fig. 2).

**Extended Data Fig. 3:**
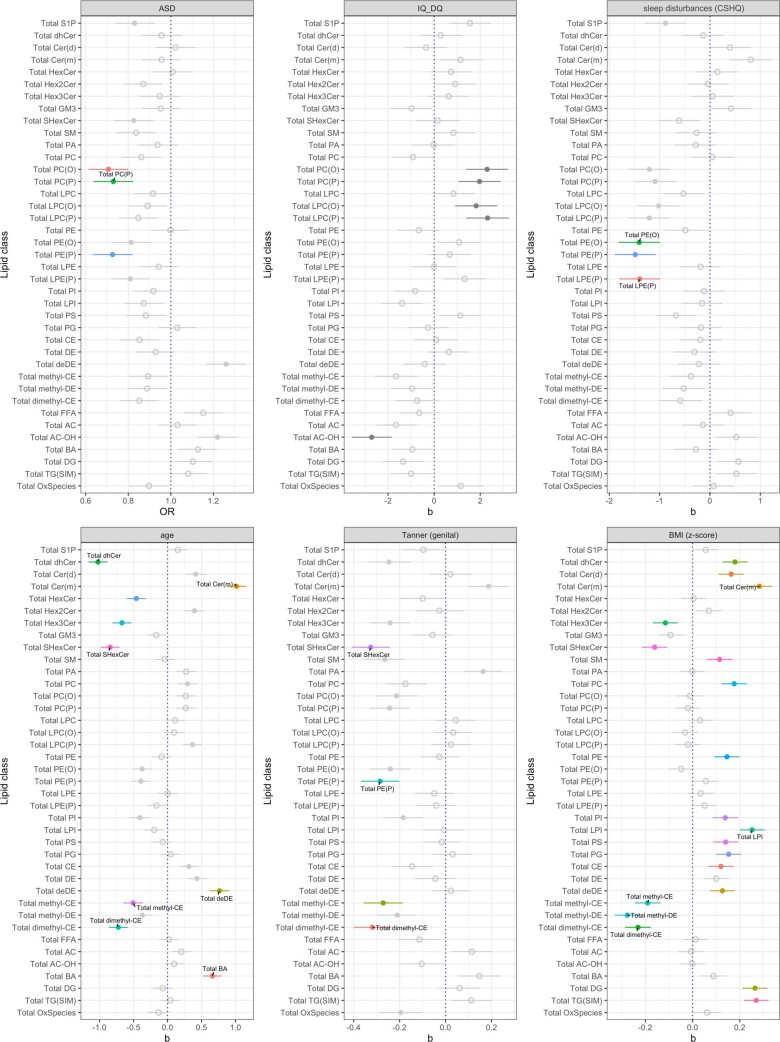
Lipidome-wide association study (LWAS) forest plots at the lipid class level. Plots shown for ASD, IQ/DQ, sleep disturbances (CSHQ total score), age, Tanner score (genital), and BMI (z-score). Point denotes effect estimate and error bars denote standard error. All analyses included covariates of injection order and storage time; the ASD, IQ/DQ and sleep disturbances LWAS also included age, age2 and sex as covariates; the age and Tanner score (genital) LWAS also included sex; the BMI (z-score) LWAS did not include additional covariates as these are accounted for within the population-normed z-score. The ASD analysis excluded storage time outliers. Open points represent classes with association p > 0.05, filled points represent classes with association p ≤ 0.05. Colour denotes classes passing multiple testing correction (dividing by the effective number of independent lipids; see Methods). For IQ/DQ, there were no significantly associated lipid classes and the nominally significant classes (p < 0.05) are shown in dark grey. Text denotes lipid classes retained in a backwards stepwise regression model with covariates, representing the effective number of independent LWAS hits.

We identified lipid species significantly associated with ASD diagnosis (*n* = 8), IQ/DQ (*n* = 8) and sleep disturbances (*n* = 20) (Extended Data Fig. 2, Supplementary Tables 4–6 and Supplementary Fig. 5a). There were also numerous species-level associations with age (*n* = 181), Tanner stage (*n* = 43), BMI (*n* = 159) and sex (*n* = 71) (Extended Data Fig. 2 and Supplementary Tables 7–10), consistent with the strong associations in the variance component analyses (Fig. 2b). We also identified lipid class associations (Extended Data Fig. 3). Sensitivity analyses gave consistent results (Supplementary Figs. 6–10 and Methods).

**Fig. 3:**
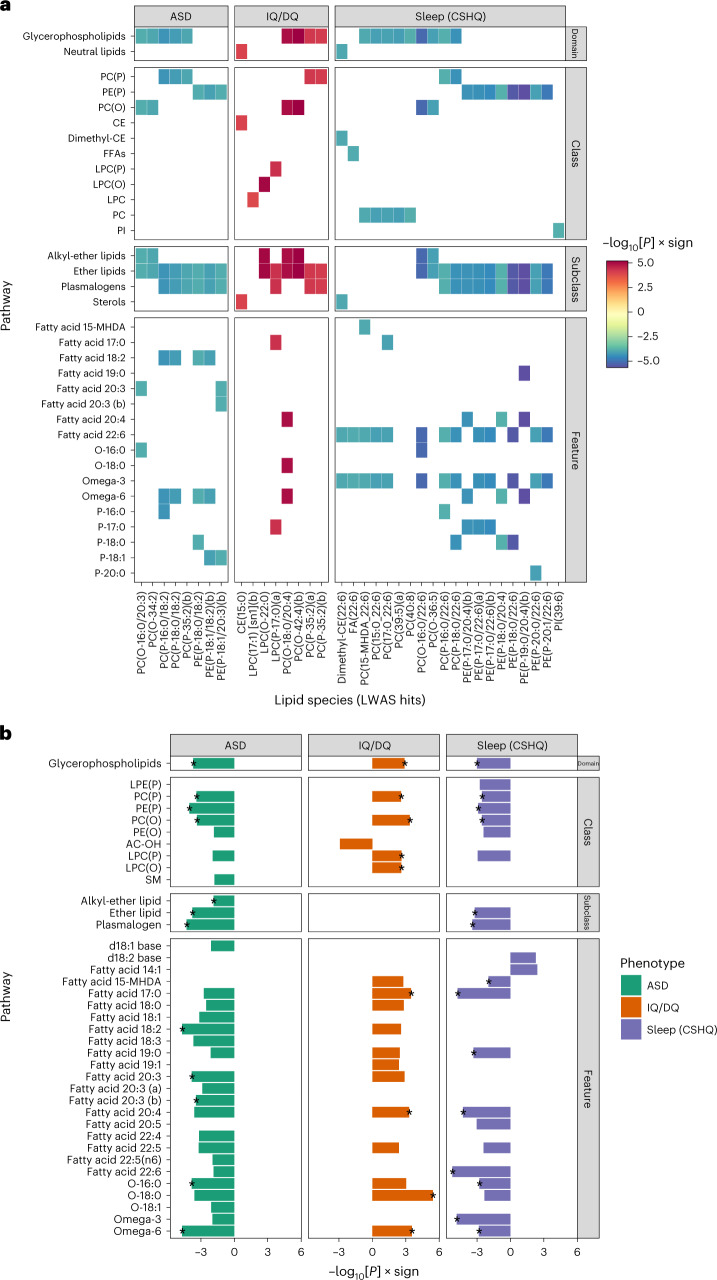
Lipid pathways and annotations associated with ASD, IQ/DQ and sleep disturbances. **a**, Tile plot showing trait-associated lipid species from LWAS (*x* axis) and higher-level annotations (*y* axis). The values on the color scale bar show −log_10_[*P*] multiplied by the test statistic sign. 15-MHDA, 15-methylhexadecanoic acid; CE, cholesteryl ester; dimethyl-CE, dimethylcholesteryl ester; FFA, free fatty acid; LPC, lysophosphatidylcholine; LPC(O), lysoalkylphosphatidylcholine; LPC(P), lysoalkenylphosphatidylcholine; PC, phosphatidylcholine; PC(O), alkylphosphatidylcholine; PC(P), alkenylphosphatidylcholine; PE(P), alkenylphosphatidylethanolamine; PI, phosphatidylinositol. **b**, LSEA results for ASD diagnosis, IQ/DQ and sleep disturbances (CSHQ total score). FDR-significant results (*q* < 0.05) are shown. The rows show lipid annotations. The bar lengths represent −log_10_[*P* value for the LSEA] multiplied by the test statistic sign. Asterisks represent annotations that were also significant by LWAS. Linoleic acid-containing lipid species map to fatty acid 18:2 and omega-6 features. Arachidonic acid-containing lipid species map to fatty acid 20:4 and omega-6 features. DHA-containing lipid species map to fatty acid 22:6 and omega-3 features. LC-PUFAs (including linoleic acid, arachidonic acid and DHA) correspond to lipids with both long fatty acid chains (18 carbons or more) and omega-3 or omega-6 features. At the feature hierarchical level, there were sometimes multiple annotations relating to a single feature (for example, feature–omega-6 as well as subclass–plasmalogen | feature–omega-6), so the values in **b** correspond to the most significant annotation. We have ensured that these match the LWAS hits (Supplementary Tables 11–13). AC-OH, hydroxylated acylcarnitine; LPE(P), alkenyllysophosphatidylethanolamine; PE(O), alkylphosphatidylethanolamine; SM, sphingomyelin.

**Extended Data Fig. 4:**
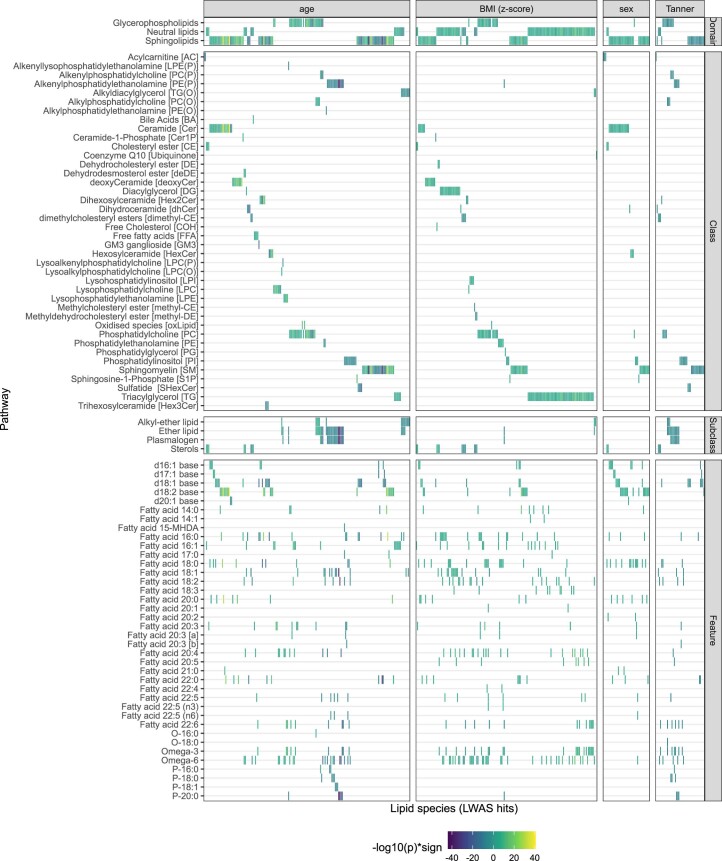
Lipid species annotations for age, BMI, sex and Tanner score. Annotated lipid species significantly associated with age, BMI (z-score), sex and Tanner score (genital) in the LWAS analysis.

**Extended Data Fig. 5:**
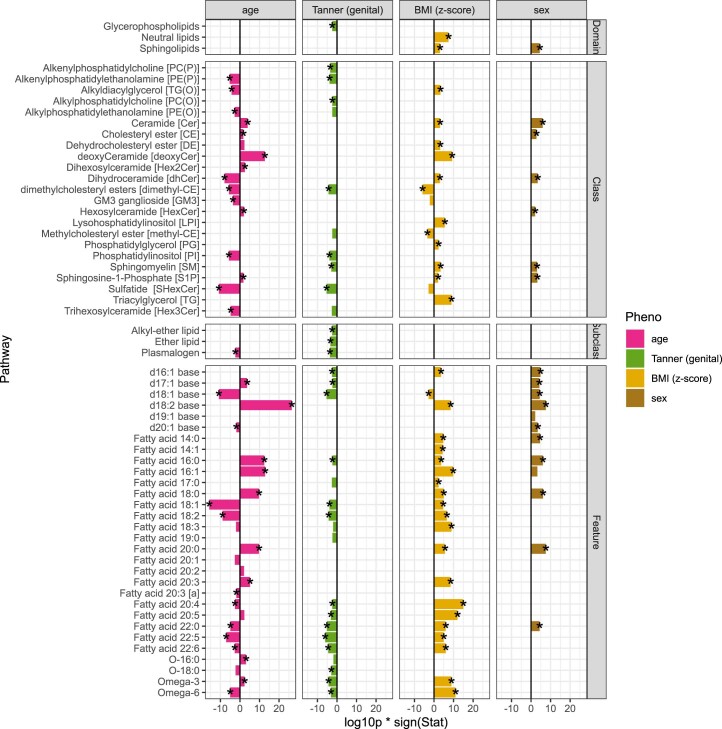
Lipid-set enrichment analysis (LSEA) results for age, Tanner score (genital) and BMI (z-score). Results shown are FDR-significant (q < 0.05). Rows: lipid annotations. Length of bars: -log10 p-value for the LSEA analysis, multiplied by the test statistic sign. *: annotations that were also significant in LWAS.

To interpret the LWAS hits, we assigned functional annotations using a lipid ontology (Fig. 3a and Extended Data Fig. 4). Across the neurodevelopmental traits, multiple LWAS hits mapped to long-chain polyunsaturated fatty acids (LC-PUFAs): between ASD diagnosis and decreased linoleic acid (ontology terms fatty acid 18:2 and omega-6; also a precursor to other LC-PUFAs); between sleep disturbances and decreased docosahexanoic acid (DHA; terms fatty acid 22:6 and omega-3); and both sleep disturbances and decreased IQ/DQ were associated with decreased arachidonic acid (terms fatty acid 20:4 and omega-6). The neurodevelopmental LWAS hits typically belonged to plasmalogen subclasses (important roles in the brain include myelination, synaptic vesicles and secretory granules^23^) or ether lipid subclasses and had glycerophospholipid domains. The lipid set enrichment analysis (LSEA) was highly consistent, including for the same LC-PUFAs: linoleic acid, arachidonic acid and DHA (Fig. 3b and Supplementary Tables 11–13). We also performed LSEAs for age, Tanner score, BMI and sex (Extended Data Fig. 5 and Supplementary Tables 14–17).

### Validation of LWAS hits using genetic data

**Fig. 4:**
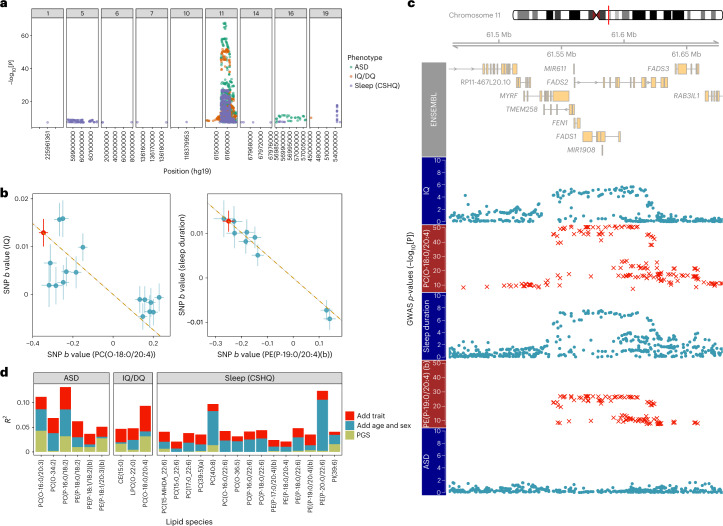
Genetic contributions of LWAS hits for ASD, IQ/DQ and sleep disturbances. **a**, Plots showing chromosomes with genetic signals for the lipid species associated with ASD, IQ/DQ and sleep disturbances. **b**, Effect sizes (*b*) of SNPs in the chromosome 11 *FADS* gene region (used for the HEIDI test) from GWAS summary statistics for two lipid–neurodevelopmental trait pairs: IQ–PC(O-18:0/20:4) and sleep duration–PE(P-19:0/20:4)(b). Red indicates the SMR instrument—the SNP with the most significant association with both the lipid and neurodevelopmental trait in AAB/QTAB (Extended Data Fig. 6). The gold dashed line represents the estimate from SMR of the effect of the lipid on the neurodevelopmental trait at the instrumental SNP (*b*_*xy*_), rather than the regression line. **c**, Plot of the chromosome 11 *FADS* gene region (top), with Manhattan plots showing colocalization of the genetic signal from lipid (red plots) and neurodevelopmental (blue plots) trait GWASs. **d**, Bar plot of the variance (*R*^2^) explained in the ANOVA model with terms ordered as: lipid species ~ PGS + demographic information (age, age^2^ and sex) + trait (ASD, IQ/DQ or CSHQ total score). The displayed lipids are limited to those with summary statistics in the BHS lipid GWAS, which were used to generate the PGSs. The sleep-associated lipid PC(P-18:0/22:6) has *R*^2^_PGS_ = 0 as all participants had identical genotypes at the *n* = 2 PGS loci.

In the absence of an equivalent pediatric dataset with matching lipidomic and phenotypic data, and given that the adult lipidome has a strong genetic basis^24^, we used genetic data to replicate lipid–neurodevelopmental trait associations. Among the 36 lipid species LWAS hits for the three neurodevelopmental traits, 24 had genome-wide association study (GWAS) summary statistics from the Busselton Health Study (BHS)^24^ (data generated by the same laboratory on a slightly smaller lipid panel). Aggregating the genome-wide-significant single-nucleotide polymorphisms (SNPs) for lipids significantly associated with ASD, IQ/DQ and sleep disturbances, the majority of SNPs localized to the *FADS* gene cluster on chr11:61.4–61.7 Mb (Fig. 4a). Fatty acid desaturase (FADS) enzymes catalyze the two steps in the conversion of linoleic acid to arachidonic acid, which is consistent with the LWAS hit annotations (Fig. 3a). This colocalization of genetic signal across multiple lipid species is consistent with strong phenotypic correlation across the lipidome (Extended Data Fig. 1).

**Extended Data Fig. 6:**
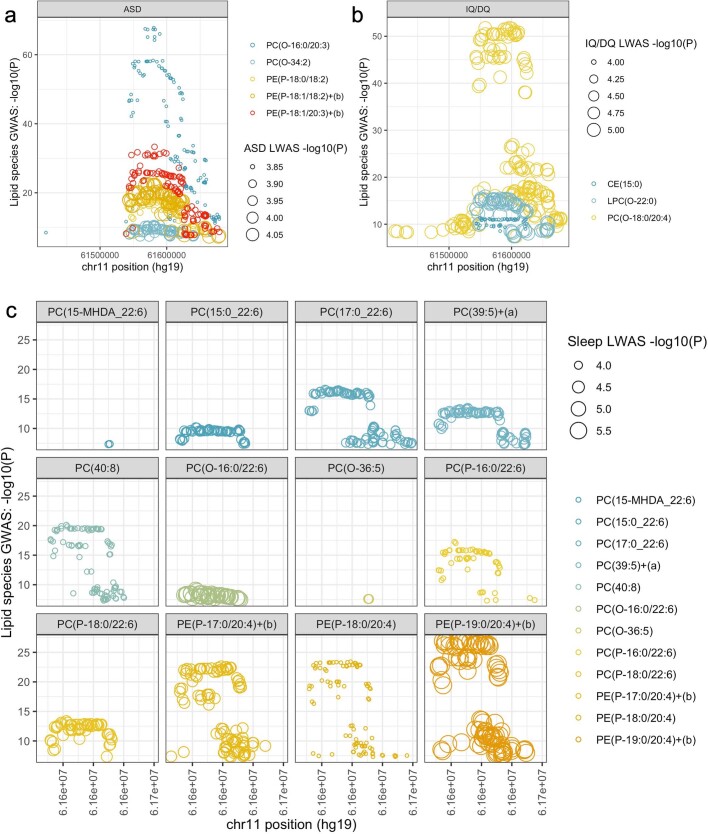
GWAS locus plots (from the Busselton Health Study) in the FADS gene cluster on chromosome 11 for neurodevelopment-associated lipids. Lipids shown here were significant in the AAB+ QTAB LWAS for **a**) ASD, **b**) IQ/DQ, **c**) sleep disturbances. Colours respond to individual lipids. The size of the open circle is proportional to the -log10(P) between that lipid species and the neurodevelopmental trait. In c), the sleep disturbances-associated lipids have separate panels for readability. Note that only the LWAS significant lipids that also have summary statistics in the Busselton Health Study are shown here, hence why there are fewer lipids here than in Fig. 3a.

Leveraging this strong genetic signal for neurodevelopment-associated lipids, we investigated potential pleiotropy or causality between lipids and neurodevelopmental traits. We applied summary data-based Mendelian randomization (SMR) by integrating GWAS summary statistics from independent studies, choosing a locus-specific method (over a genome-wide one) as lipids have an oligogenic architecture^24^. We selected lipids with: (1) neurodevelopmental trait associations in AAB/QTAB; and (2) strong chromosome 11 *FADS* region genetic effects in the external BHS lipidomics GWAS^24^. Hence, for IQ/DQ, we chose the phosphatidylcholine PC(O-18:0/20:4) and for sleep disturbances we chose the phosphatidylethanolamine PE(P-19:0/20:4)(b); both have arachidonic acid groups and both had strong LWAS associations and strong genetic effects related to *FADS* genes (Extended Data Fig. 6). There was no genetic signal for ASD in the *FADS* region (Fig. 4c), so we did not apply SMR to any ASD–lipid combination. For the neurodevelopmental GWAS summary statistics, we used intelligence quotient (IQ)^25^ and sleep duration^26^ GWAS summary statistics as proxy traits. We then chose the most strongly associated SNP for the lipid trait as the SMR instrument: rs99780 for the IQ/PC(O-18:0/20:4) GWAS lipid trait pair and rs102274 for the sleep duration/PE(P-19:0/20:4)(b) GWAS lipid trait pair.

SMR identified strong associations between PC(O-18:0/20:4) and IQ (*b* = −0.037; s.e. = 0.009; *P*_SMR_ = 1.7 × 10^−5^) and between PE(P-19:0/20:4)(b) and sleep duration (*b* = −0.051; s.e. = 0.011; *P*_SMR_ = 1.37 × 10^−6^) (Fig. 4b,c). However, only the latter passed the Heterogeneity in Dependent Instruments (HEIDI) test (IQ/PC(O-18:0/20:4) *P*_HEIDI_ = 1.1 × 10^−3^ (*n* = 20 SNPs) and sleep duration/PE(P-19:0/20:4)(b) *P*_HEIDI_ = 0.96 (*n* = 20 SNPs)), suggesting that the IQ/PC(O-18:0/20:4) association may be due to genetic linkage, whereas the sleep duration/PE(P-19:0/20:4)(b) association is consistent with a SNP-to-lipid-to-trait causal (or pleiotropic) relationship (Supplementary Table 18).

As another replication, we genetically predicted lipid levels and investigated their neurodevelopmental associations in the Adolescent Brain Cognitive Development (ABCD) study: a larger population-ascertained pediatric dataset. The directions of effect were consistent but not statistically significant (for IQ/PC(O-18:0/20:4), *b* = 4.6 × 10^−2^ (s.e. = 1.8 × 10^−2^; *P* = 0.79); for sleep disturbances/PE(P-19:0/20:4)(b), *b* = −2.1 × 10^−3^ (s.e. = 3.0 × 10^−3^; *P* = 0.48)), probably because genetic prediction of lipids in pediatric cohorts using adult summary statistics is imperfect (Methods) and because the *FADS* locus explains only a fraction of variance in sleep and cognitive traits.

We noted that the chromosome 11 locus is associated with multiple lipids that were also associated with neurodevelopmental traits in AAB/QTAB. To capture the locus’ pleiotropic effects, we looked for associations between neurodevelopmental traits and predicted gene expression. We prioritized *FADS1*, *FADS2* and *TMEM258* for individual-level transcriptome-wide association study (TWAS) analyses using evidence from multistep SMR mapping gene expression–lipid trait associations (Supplementary Table 18). There were no associations between genetically predicted expression and neurodevelopmental traits in the AAB/QTAB dataset or the larger ABCD dataset (*n* = 4,592). However, genetically predicted *FADS1* (*R*^2^ = 3.93% and *P* = 1.6 × 10^−6^ for the prefrontal cortex; *R*^2^ = 2.56% and *P* = 1.1 × 10^−4^ for whole blood), *FADS2* (*R*^2^ = 2.01% and *P* = 6.4 × 10^−4^ for whole blood) and *TMEM258* (*R*^2^ = 3.11% and *P* = 2.1 × 10^−5^ for whole blood) could predict plasma PC(O-18:0/20:4) levels in AAB/QTAB (Methods).

### Dissecting contributions to neurodevelopment-associated lipids

To contextualize our ability to replicate our findings using genetic data, we took the *n* = 646 AAB/QTAB children of European ancestry and generated polygenic scores (PGS) for 215 trait-associated lipid species from the LWAS analyses (Supplementary Tables 4–10) using BHS GWAS summary statistics. We confirmed that the lipid PGS significantly predicted 152 out of 215 lipid species (analysis of variance (ANOVA); *P* < 0.05; 145 out of 215 after Benjamini–Hochberg correction) and checked that selected lipid GWAS results within the AAB/QTAB European cohort were comparable to the BHS GWAS results (Supplementary Fig. 11 and Methods).

**Extended Data Fig. 7:**
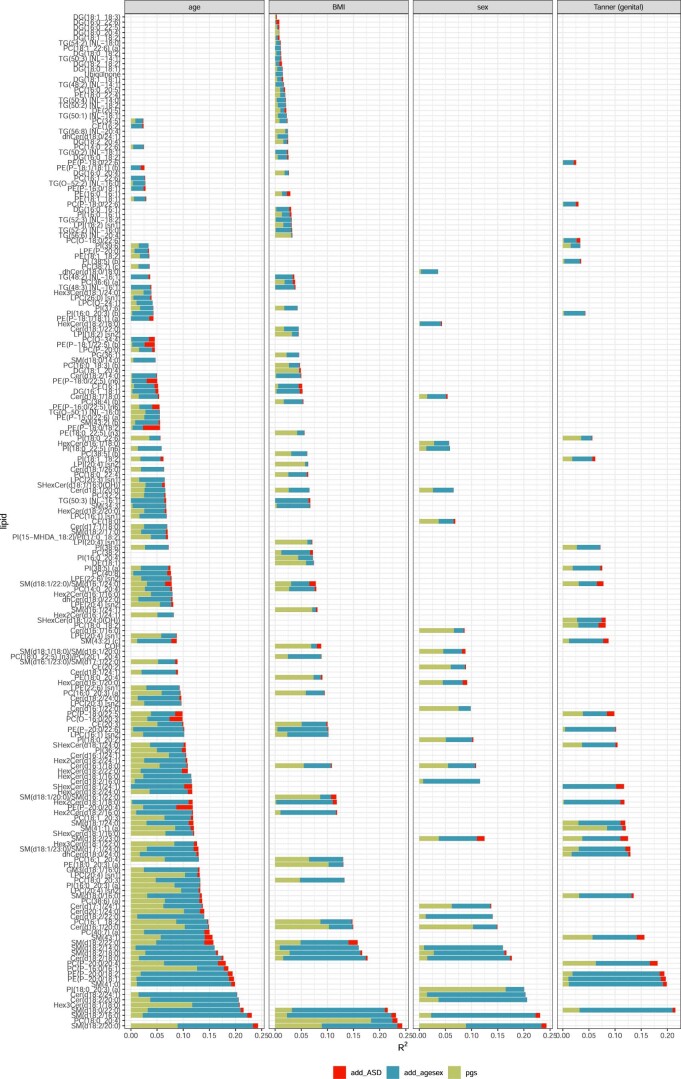
Bar plot of variance (R^2^) in lipid concentration associated with polygenic score (PGS), demographic variables and ASD diagnosis, for lipid species significantly associated with age, BMI (z-score), sex and Tanner score. ANOVA model: lipid concentration ~ PGS + (age and sex) + ASD diagnosis.

Next, we dissected the relative contributions of common genetic variation (lipid PGS) and demographic, batch, dietary and neurodevelopmental factors to lipid variance. For neurodevelopment-associated lipids, up to 13% of variance could be explained by lipid PGS, demographic variables and the neurodevelopmental trait of interest (Fig. 4a and Extended Data Fig. 7). Batch variables generally made little contribution (Supplementary Figs. 12–14). Adding dietary principal components explained significant variance in lipid concentrations with a total *R*^2^ of up to 25% for the neurodevelopmental traits (Supplementary Figs. 12 and 15).

### Environmental associations of the neurodevelopmental lipidome

**Extended Data Fig. 8:**
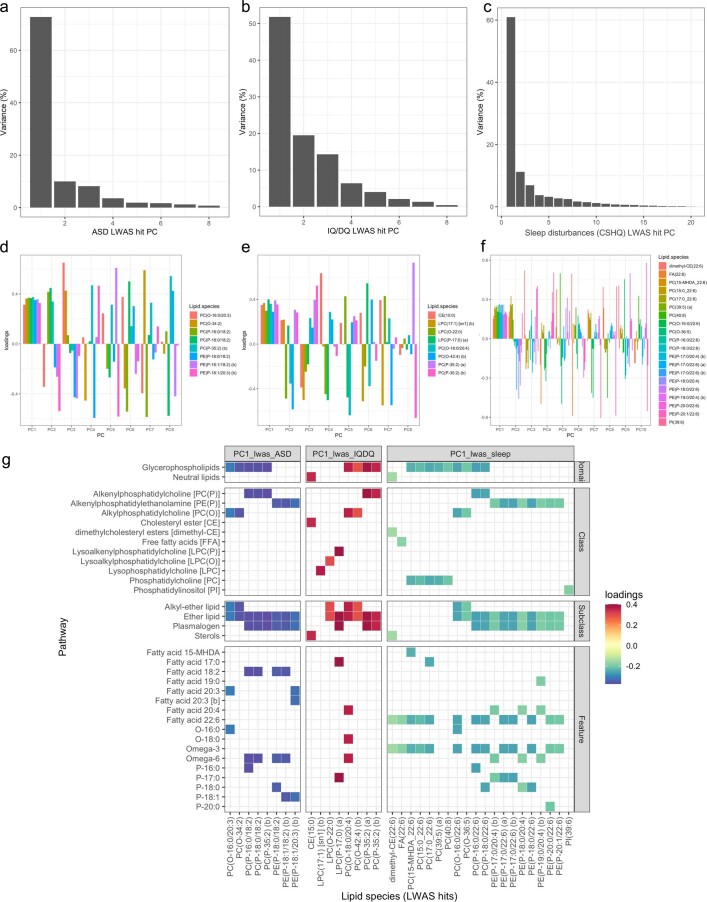
Validation of the LWAS PC1 as ‘lipidome profiles’ associated with ASD diagnosis, IQ/DQ and sleep disturbances. A LWAS PC1 was calculated for each neurodevelopmental trait by performing PCA on the statistically significant LWAS lipid species. **a**-**c**) Scree plots demonstrating that LWAS PC1 captures >50% of variance in each set of trait-associated lipids; **d**-**f**) Plot of PCA loadings, demonstrating that PC1 captures increased levels of all trait-associated lipids. **g**) Heatmap demonstrating that PCs are representative of LC-PUFAs: there are strong loadings for the annotations corresponding with linoleic acid for the ASD LWAS hits PC1 (that is, fatty acid 18:2 and Omega-6), arachidonic acid (that is, fatty acid 20:4 and Omega-6) for the IQ/DQ LWAS hits PC1, and DHA (that is, fatty acid 22:6 and Omega-3) and arachidonic acid for the sleep LWAS hits PC1.

We investigated the potential contributions of environmental variables to neurodevelopment-associated lipidome profiles. To capture a trait’s lipidome profile in a single variable, we performed principal component analysis (PCA) on the significant LWAS associations (species level) and took PC1. We verified that each trait’s lipidome profile explained the majority (50–70%) of variance in the focal trait’s LWAS associations (Extended Data Fig. 8), that they primarily captured the intended variable (Supplementary Figs. 16–18), that the direction of effect of the loadings matched the LWAS effect direction and that, as expected, the lipidome profiles were strongly associated with their respective focal traits (for ASD, *b* = −0.83 (s.e. = 0.18; *P* = 8.7 × 10^−6^; *n* = 694); for IQ/DQ, *b* = 0.02 (s.e. = 0.003; *P* = 1.1 × 10^−9^; *n* = 642); for sleep disturbances, *b* = −0.073 (s.e. = 0.014; *P* = 3.2 × 10^−7^; *n* = 607)).

**Fig. 5:**
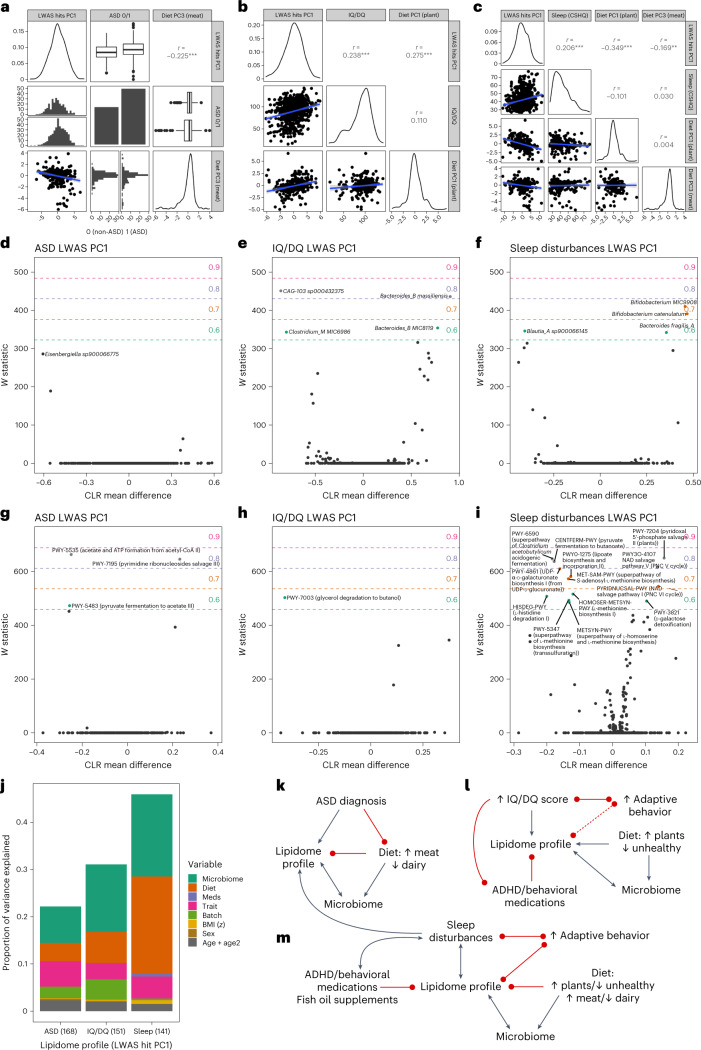
Relationships between neurodevelopmental traits, their lipidome profiles (LWAS hit PC1s) and dietary and microbiome variables. **a**–**c**, Relationships between ASD diagnosis (**a**), IQ/DQ (**b**) sleep disturbances (**c**), and their respective lipidome profiles and dietary profiles. The ASD and sleep disturbances lipidome profiles were multiplied by −1 to align the direction of effect. For the ASD plots (**a**), the right half of the second column corresponds to the ASD group (left half corresponds to the non-ASD group), as does the bottom half of the second row (top half of the second row corresponds to the non-ASD group). Upper triangle of **a**–**c** provides Pearson’s correlation coefficients and asterisks denote significance thresholds: **P <0.01, ***P <0.001. Box and whisker plots denote quartiles. **d**–**i**, Differentially abundant microbiome species (**d**–**f**) and MetaCyc pathways (**g**–**i**) for the neurodevelopmental lipidome profiles ASD LWAS PC1 (**d**,**g**), IQ/DQ LWAS PC1 (**e**,**h**) and sleep disturbances LWAS PC1 (**f**,**i**) (covariates: age, age^2^, sex, batch, injection order and dietary PC1–PC3). The microbiome analysis sample size was *n* = 188. The *x*-axis (CLR mean difference) indicates the effect size on the centered log-ratio transformed scale, whereas *y*-axis (W statistic) indicates the degree of statistical significance, whereby W statistic > 0.7 indicates robust significance, whereas > 0.6 corresponds to nominal significance. **j**, Dissection of variance in the neurodevelopmental lipidome profiles. The results are from ANOVA models of the trait-specific lipidome profile ~ age + age^2^ + sex + BMI + batch (batch, injection order and storage duration) + neurodevelopmental trait + significantly associated medications (meds) + significantly associated dietary principal components (**a**–**c**) + significant microbiome features (**d**–**i**). **k**–**m**, Proposed models of the relationships between neurodevelopmental lipidome profiles (for ASD diagnosis (**k**), IQ/DQ (**l**), sleep problems (**m**)), neurodevelopmental traits, diet, microbiome, medications and adaptive function. The dashed line indicates that the lipidome association is not independent (that is, the association between adaptive function and the IQ/DQ lipidome profile can be explained by IQ/DQ lipidome associations). Bidirectional arrows indicate either bidirectional relationships or insufficient evidence (previous or otherwise) to suggest a direction of causality. The trait-specific lipidome profiles were the variables of interest, so analyses were not exhaustively performed between other variable pairs. The black arrows represent positive associations and the red arrows represent negative associations.

For ASD, we identified significant three-way interactions between the ASD lipidome profile, ASD diagnosis and reduced meat consumption (captured by dietary PC3), suggesting that the ASD lipidome profile could be partially attributed to reduced meat intake among the ASD group (linear model (ASD lipidome profile ~ dietary PC3 + ASD diagnosis + covariates): *b* = −0.34 (s.e. = 0.12; *P* = 4.3 × 10^−3^; *n* = 261) for dietary PC3 and *b* = 0.88 (s.e. = 0.32; *P* = 7.0 × 10^−3^) for ASD diagnosis) (Fig. 5a,k). These findings are consistent with the LWAS annotations of reduced linoleic acid, for which meat is a primary source^27^. Variance in the ASD lipidome profile was also associated with stool microbiome features (genetic potential for decreased gut microbial acetate production and increased pyrimidine ribonucleoside salvage) (Fig. 5d,g,k, Supplementary Fig. 19 and Supplementary Tables 19 and 20). Medications made limited contributions to the ASD lipidome profile.

The IQ/DQ lipidome profile was independently and more strongly associated with a plant-based, healthy diet (dietary PC1) than IQ/DQ itself (linear model (IQ/DQ lipidome profile ~ dietary PC1 + IQ/DQ + covariates): *b* = 0.42 (s.e. = 0.10; *P* = 1.7 × 10^−5^; *n* = 235) for dietary PC1 and *b* = 1.4 × 10^−2^ (s.e. = 0.6 × 10^−2^; *P* = 2.8 × 10^−2^; *n* = 235) for IQ/DQ). There was no direct association between the dietary and IQ/DQ measures (linear model (IQ/DQ ~ dietary PC1 + covariates): *b* = 1.66 (s.e. = 0.98; *P* = 0.09; *n* = 235) for dietary PC1), suggesting convergent and independent associations with the IQ/DQ lipidome profile (Fig. 5b,l). The IQ/DQ lipidome profile was associated with reduced stool microbiome genetic potential for glycerol degradation to butanol, as well as increased *Bacteroides_B massiliensis* and decreased *CAG-103 sp000432375* (Fig. 5e,h,l, Supplementary Fig. 19 and Supplementary Tables 21 and 22). While IQ/DQ was associated with multiple medications, only ADHD/behavioral medications were robustly associated with a lower IQ/DQ lipidome profile, after conditioning on the measured IQ/DQ score (ANOVA (IQ/DQ lipidome ~ IQ/DQ + medications): *R*^2^ = 1.4% and *P* = 2.2 × 10^−3^ for ADHD/behavioral medications).

The sleep disturbances lipidome profile had the most complex interactions, being associated with decreased intake of a plant-based healthy diet (linear model (sleep disturbances lipidome profile ~ dietary PC1 + sleep problems + covariates): *b* = −0.91 (s.e. = 0.15; *P* = 1.4 × 10^−9^; *n* = 261) for dietary PC1) and meat (linear model (sleep disturbances lipidome profile ~ dietary PC3 + sleep problems + covariates): *b* = −0.46 (s.e. = 0.17; *P* = 8.3 × 10^−3^; *n* = 261) for dietary PC3) (Fig. 5c,m). The effects of sleep disturbances and diet on the sleep disturbances lipidome profile were primarily independent (joint model: *b* = 0.08 (s.e. = 0.02; *P* = 1.1 × 10^−3^; *n* = 201) for sleep disturbances, *b* = −0.90 (s.e. = 0.17; *P* = 4.4 × 10^−7^; *n* = 201) for dietary PC1 and *b* = −0.49 (s.e. = 0.18; *P* = 6.6 × 10^−3^; *n* = 201) for dietary PC3) (Fig. 5c,m). The sleep disturbances lipidome profile was associated with increased stool microbiome metabolic potential for pyridoxal 5′-phosphate salvage and nicotinamide adenine dinucleotide (NAD) salvage and decreased metabolic potential for *Clostridium acetobutylicum* acidogenic fermentation, pyruvate fermentation to butanoate, *S*-adenosyl-l-methionine biosynthesis, UDP-α-d-galacturonate biosynthesis and lipoate biosynthesis and incorporation, in addition to positive associations with *Bifidobacterium MIC 9908* and *Bifidobacterium catenulatum* (Fig. 5f,i, Supplementary Fig. 19 and Supplementary Tables 23 and 24). There were overlapping associated microbiome features between the sleep disturbances lipidome profile and both dietary PC1 and dietary PC3 (Fig. 5f,i and Supplementary Figs. 19 and 20). ADHD/behavioral medication and fish oil/DHA supplements were negatively associated with the sleep disturbances lipidome profile and explained significant variance independently of the measured sleep disturbances score (ANOVA (sleep disturbances lipidome ~ sleep disturbances + medications): *R*^2^ = 1.7% and *P* = 9.5 × 10^−4^ for ADHD/behavioral medication and *R*^2^ = 3.1% and *P* = 7.0 × 10^−6^ for fish oil/DHA supplements). The positive association of ADHD medications with sleep disturbances (here and in other studies^28^) is expected given that these medications are stimulants that interfere with sleep, whereas fish oil supplementation may improve sleep^29^ and can also lower triglycerides^30^.

**Extended Data Fig. 9:**
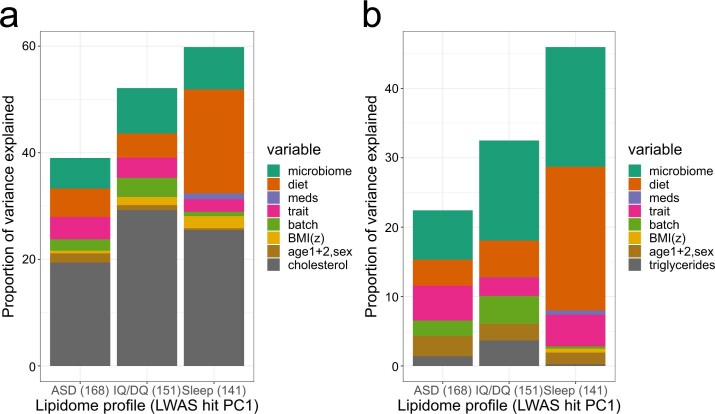
Dissection of variance in neurodevelopmental trait lipidome profiles after conditioning on total lipidome (a) cholesterol and (b) triglycerides. Similar to Fig. 5, results are from ANOVA models of the trait-specific lipidome profile ~ age + age^2^ + sex + BMI + batch (batch, injection order, storage duration) + neurodevelopmental trait + significantly-associated medications + significantly-associated dietary PCs (Fig. 5a–c) + significant microbiome features (Fig. 5d–i).

To compare the relative strengths of relationships between trait lipid profiles and associated variables, we performed ANOVA (Fig. 5j), noting that this restricted the dataset to individuals with complete data. The neurodevelopmental traits, diet and the microbiome each made sizeable contributions to variance in the neurodevelopmental lipidome profiles (Fig. 5j). With regard to variance in the observed neurodevelopmental traits, the lipidome profile consistently explained significant variance, whereas dietary variables (dietary PC3) significantly increased variance for ASD diagnosis only, implying a close relationship between ASD and dietary differences. Medications overall made small contributions to variance in trait lipidome profiles because relatively few individuals were taking these medications (*n* = 19 ADHD/behavioral and *n* = 27 fish oil/DHA). In sensitivity analyses conditioning all other covariates on clinical lipid levels (Extended Data Fig. 9), the primary results persisted.

### Interplay between neurodevelopmental traits and the lipidome

We hypothesized that the ASD lipidome may reflect co-occurring conditions better than ASD itself (given the strong association between diet and the ASD lipidome in this cohort) and investigated sleep as a mediating factor. The relationship between ASD and the ASD lipidome profile was attenuated when adjusting for sleep disturbances and demographic and batch covariates (*R*^2^ = 1.4% (*P* = 7.7 × 10^−2^) to *R*^2^ = 0.96% (*P* = 2.7 × 10^−2^); Methods). Conversely, for the relationship between sleep disturbances and the respective lipidome profile, conditioning on ASD diagnosis and covariates had little effect on the primary association (*R*^2^ = 3.3% (*P* = 3.6 × 10^−5^) to *R*^2^ = 3.1% (*P* = 6.0 × 10^−5^); Methods).

Next, we investigated whether omics data relate to adaptive function for children with autism. We used the VABS-II composite score (only collected within the ASD group), which is strongly correlated with increased IQ/DQ and decreased sleep disturbances (univariate linear models: *b* = 0.36 (s.e. = 0.03; *P* = 3.5 × 10^−33^; *n* = 331) for IQ/DQ and *b* = −0.32 (s.e. = 0.07; *P* = 1.3 × 10^−5^; *n* = 356) for sleep disturbances). We found that the sleep disturbances lipidome profile explained significant variance in adaptive function independent of the measured sleep disturbances score (*R*^2^ = 3.2% (*P* = 3.2 × 10^−2^) to *R*^2^ = 1.7% (*P* = 1.7 × 10^−2^)). In contrast, the relationship between adaptive function and the IQ/DQ lipidome profile was dependent on IQ/DQ score (*R*^2^ = 2.3% (*P* = 2.9 × 10^−3^) to *R*^2^ = 1.3 × 10^−5^ (*P* = 0.94)).

### Lipidome outliers

Finally, we investigated group differences in variance for measured lipids, reasoning that centrality (for example, mean) measures may not capture the intrinsic heterogeneity of ASD (for example, only a subset of the ASD group may have altered lipid profiles). Furthermore, lipid levels have a relatively oligogenic genetic architecture, which could overlap ASD-associated genetic regions (some of which include large regions of structural genetic variation).

**Extended Data Fig. 10:**
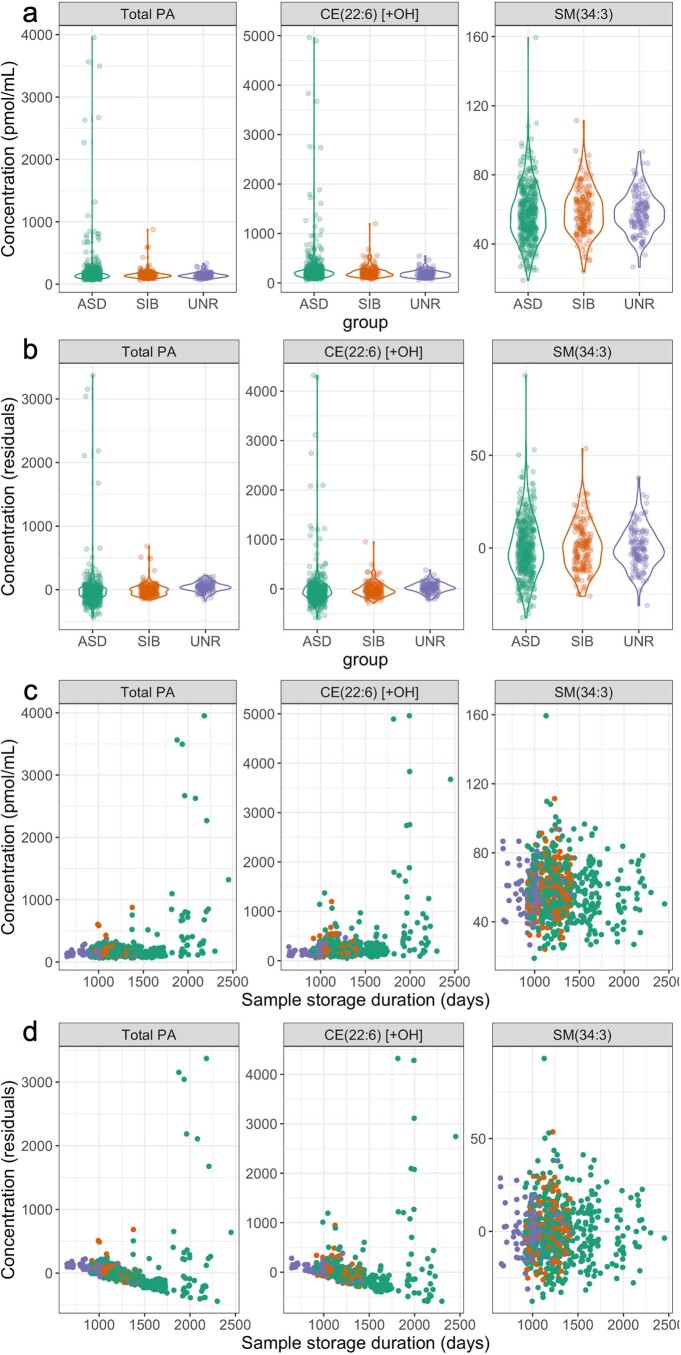
Group differences in lipid variance. (**a**, **b**) Violin plots for lipid classes and species with significant differences in variance (Levene’s test), before (**a**) and after (**b**) regressing lipid concentration against age, age^2^, sex, batch, injection order and storage duration. (**c**) Assessing the relationship between lipid classes and species with significant differences in variance and sample storage duration before (**c**) and after (**d**) regressing out covariates.

First, we investigated whether the ASD group had greater variance in lipids versus the combined SIB plus UNR group. After regressing out covariates (age, age^2^, sex, batch, injection order and storage duration) and excluding storage duration outliers, we found that the sphingomyelin SM(34:3) was significantly more variable in the ASD group after multiple testing correction (Supplementary Table 25). The other significantly variable lipids appeared to be driven by sample degradation, despite adjustment for sample storage duration (Extended Data Fig. 10).

**Fig. 6:**
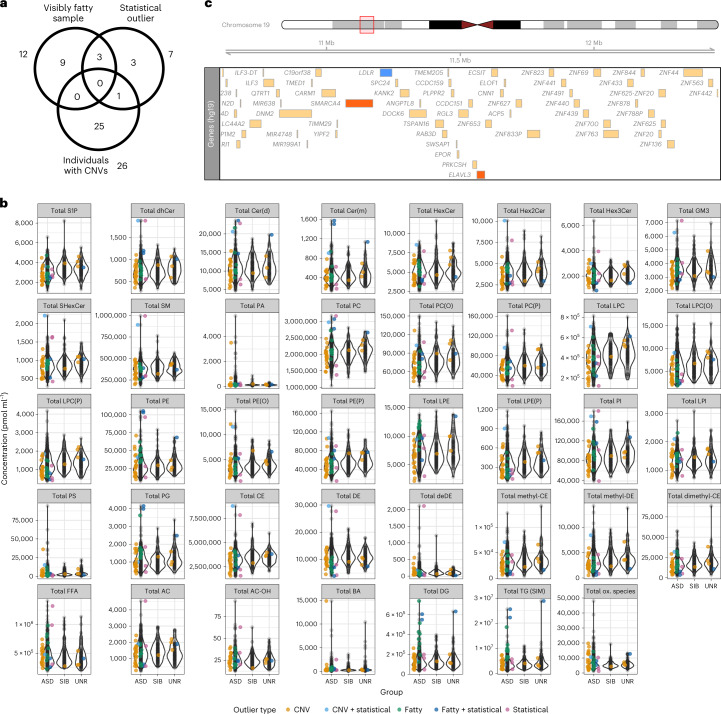
Outlier analysis. **a**, Venn diagram showing the overlap between three groups of outliers: visibly fatty samples (total *n* = 12), statistical outliers (total *n* = 7) and individuals with large, rare CNVs (total *n* = 26). **b**, Violin plots showing the distribution of lipid class concentrations by group. Coloured points indicate where outliers sit within the overall distribution and denote the outlier category. AC, acylcarnitine; BA, bile acid; Cer, ceramide; DE, dehydrocholesteryl ester; deDE, dehydrodesmosterol ester; DG, diacylglycerol; dhCer, dihydroceramide; GM3, GM3 ganglioside; HexCer, hexosylceramide; Hex2Cer, dihexosylceramide; Hex3Cer, trihexosylceramide; methyl-CE, methylcholesteryl ester; methyl-DE, methyldehydrocholesteryl ester; PA, phosphatidic acid; PE, phosphatidylethanolamine; PG, phosphatidylglycerol; S1P sphingosine-1-phosphate; SHexCer, sulfatide; ox., oxidized. **c**, Locus plot showing the CNV deletion region for the individual who was in both the CNV and statistical outlier groups. Blue indicates the *LDLR* gene, which is well known for its association with lipid traits. Red indicates the *SMARCA4* and *ELAVL3* genes, which are high-confidence ASD genes in the Simons Foundation Autism Research Initiative database.

Next, we investigated three partially overlapping groups for which there was a priori expectation of outlying lipid levels: (1) statistical outliers (*n* = 7); (2) visibly fatty plasma samples (*n* = 12); and (3) individuals with copy number variants (CNVs; either clinically significant or large, rare CNVs, both called using genotyping array data^31^; *n* = 26) (Fig. 6a,b).

Four of the seven statistical outlier samples had biological explanations. Three were among the *n* = 12 visibly fatty plasma samples and had the three highest concentrations of total deoxyceramide and triglycerides and three of the four highest total diacylglycerol levels (Fig. 6b). Two of these three outliers with fatty plasma were siblings, only one of whom reported the current use of methylphenidate, suggesting a shared effect (genetic or environmental) that is not explained by this medication. Statistical outliers were enriched for samples that were also fatty (OR = 26.5; 95% CI = 4.0–135.4; *P* = 6.4 × 10^−4^; Fisher’s exact test comparing three out of 12 statistical outliers that were fatty with seven out of 758 statistical outliers in the total sample).

The fourth outlier sample belonged to a participant with a chr19p13.2 CNV deletion (chr19:10609319–12464435; Fig. 6c) encompassing the low-density lipoprotein receptor (*LDLR*) gene that has key roles in cholesterol regulation. This CNV also spans ASD-associated genes in the Simons Foundation Autism Research Initiative’s SFARI Gene database^32^, including *ELAVL3* and *SMARCA4*. Rare genetic variation in *LDLR* causes familial hypercholesterolemia and common *LDLR* variants are associated with high cholesterol^24^. Furthermore, others have found a five-exon cluster of the *LDLR* gene that carries ASD-segregating variation^9^. This participant’s lipidome was consistent with high levels of LDL particles, with the highest plasma concentrations of cholesteryl ester and dehydrocholesterol ester, and was among the top five highest concentrations for free cholesterol. This was accompanied by high concentrations of a number of sphingolipid classes (dihydroceramides, di-hexosylceramides, sulfatide, ceramide(d), GM3 ganglioside and sphingomyelin) that are typically enriched in LDL particles, as well as a number of phospholipid classes (phosphatidylcholine, lysophosphatidylcholine, alkyl- phosphatidylethanolamine and phosphatidylinositol) (Fig. 6b).

Of the *n* = 12 visibly fatty plasma samples, *n* = 7 had among the ten highest concentrations of diacylglycerols and *n* = 5 were among the ten highest for triacylglycerols and alkyldiacylglycerols, suggesting that these lipid classes may be related to fatty plasma appearance. It is possible that dietary differences or a fatty meal preceding sample collection could explain lipidome differences; however, we were unable to test this as only two out of 12 individuals had matching dietary data.

## Discussion

In a large neurodevelopmental lipidomics study, we leveraged extensive phenotypic and omics data to dissect genetic, environmental and behavioral influences on the neurodevelopmental lipidome. The pediatric plasma lipidome was associated with neurodevelopmental traits, including ASD diagnosis, IQ/DQ and sleep disturbances, although there were still stronger associations with age, puberty stage, sex, BMI and dietary traits. ASD diagnosis was modestly associated with lower cholesterol levels, whereas there was no association of IQ/DQ and sleep disturbances with either cholesterol or triglyceride levels. Neurodevelopmental traits were associated with LC-PUFA-containing lipids: specifically, ASD with decreased linoleic acid, IQ/DQ with increased arachidonic acid and sleep disturbances with decreased DHA and arachidonic acid. Furthermore, shared genetic signal within the *FADS* gene cluster^33^ suggested that arachidonic acid metabolism (or even omega-6 fatty acids in general) may have a pleiotropic or mediating relationship with sleep disturbances. As the genetic signal for DHA was weaker, we did not perform SMR and thus do not rule out a causal effect of DHA on sleep. While previous observational^34–37^ and small randomized controlled trials^38–41^ have investigated omega-3 or DHA supplementation in ASD, our results suggest that sleep disturbances could be the more relevant target^29^. We have focused our discussion on LC-PUFAs because they are interpretable; however, the species-level associations that we report remain largely uncharacterized and warrant further study.

We investigated the relationship between neurodevelopmental lipidome profiles and environmental factors (including diet, the gut microbiome and medication). Whereas the ASD lipidome profile appeared to be (at least in part) a consequence of diet, lipidome profiles for sleep disturbances and low IQ/DQ converged with unhealthy dietary patterns (independent of the IQ/DQ and sleep phenotypes) and with a potential mediating role for the microbiome. These findings suggest potential mechanisms with links between cardiometabolic disease and both sleep disturbances^42^ and ASD diagnosis^10^. While medications were extensively associated with these neurodevelopmental traits, only ADHD/behavioral medications and fish oil/DHA supplements affected neurodevelopmental lipidome profiles beyond that explained by the trait itself. The sleep disturbances lipidome profile was nominally associated with poorer adaptive function independent of the severity of sleep disturbances. The microbiome analysis identified an association between sleep disturbances and higher microbiome potential for NAD salvage, which is notable given that NAD^+^ levels have been linked to circadian cycle control^43–46^. There was also a decrease in microbiome potential for l-methionine biosynthesis and its derivative *S*-adenosyl-l-methionine. The latter is involved in the synthesis of neurotransmitters, including dopamine, serotonin and noradrenaline, and is being investigated as a potential therapy for depression^47,48^ (whose diagnostic criteria includes sleep disturbances).

Our findings have potential clinical implications. There may be a role for dietary LC-PUFAs or omega-3 or -6 supplementation in sleep disturbances. We also highlight the imperative to screen for and manage sleep disturbances among children with neurodevelopmental differences^49^, having found convergence in the sleep disturbances lipidome with dietary habits that may predispose to long-term health problems, as well as possible associations with poorer adaptive function. Furthermore, a participant diagnosed with ASD with a large CNV deletion in the chr19p13.2 region (which includes the *LDLR* gene and multiple high-confidence ASD genes) had a dramatically altered lipid profile reflecting higher levels of LDL particles.

Our results also have implications for ASD biomarker research. We advocate for biobanking, deep phenotyping and careful consideration of covariates to make biological inference about intermediate traits such as the lipidome, where differences reflect genetic, environmental, behavioral and technical effects. As demonstrated here and in previous work^50^, the omics signal for ASD diagnosis is weak compared with other factors, such as age, genetics, diet (which confounded ASD lipidome associations in this study) and co-occurring conditions (for example, sleep mediated some of the ASD lipidome association). Overall, omics studies in children with a pre-existing diagnosis are better suited to interrogating biological associations of co-occurring conditions and quality of life, which are of direct interest to autistic people, rather than the current focus on diagnostic biomarkers.

There are some limitations to this work. The sample collection protocol did not include fasting (and indeed this would be difficult to achieve, particularly within this population). We attempted to overcome this by performing sensitivity analyses with cosine-transformed sample collection times, to account for cyclical patterns in lipid concentrations, and did not find major differences in the results. Clinical lipids were not directly measured and were instead inferred using lipidomics measures. We used conservative methods (ANCOM version 2.1), multiple testing correction thresholds (see Methods) and covariate inclusion (including sex and storage duration, although there was some confounding with ASD diagnosis) throughout this analysis, which may have induced false negatives. The sleep disturbances and IQ/DQ findings are most accurately interpreted in an ASD-specific context (where sleep disorders commonly co-occur), and it is unclear how our results translate to the broader pediatric population. Our more complex analyses drawing on multiple phenotypic data domains relied on smaller datasets due to missingness, to the detriment of statistical power. Furthermore, our external validation attempts were limited by the absence of other large ASD lipidomics datasets. We instead took a genetic replication approach using SMR applied to external GWAS summary statistics, but this approach is imperfect as the lipidome GWAS (BHS) data were derived from an adult sample, and lipid regulation may differ in pediatric samples. The SMR results are also subject to the assumptions of Mendelian randomization. Additionally, our other validation approach—to predict lipidome levels in the external ABCD dataset—was underpowered as PGSs or individual-level TWASs explain only a fraction of heritable variance in a lipid trait, which in turn can explain only a fraction of variance in a neurodevelopmental trait. These limitations will probably only be overcome with larger and better-matched datasets and it is unlikely that methodological improvements in estimating TWAS weights are the key limiting factor.

Future, larger studies with longitudinal designs and recruitment from the broader population will be important to establish a fundamental understanding of the pediatric and neurodevelopmental lipidome. Further work is required to replicate the association between reduced LC-PUFA metabolism (that is, arachidonic acid and DHA) and sleep disturbances, to determine whether this statistical result reflects causality, and thus whether it is clinically relevant. Longitudinal studies are required to confirm relationships between childhood sleep disturbances, pediatric lipidome profiles and long-term cardiometabolic health, both in autistic people and the general population.

In conclusion, in this detailed analysis of a deeply phenotyped dataset, we highlight complex relationships between neurodevelopment, physical health, genetics (the *FADS* gene cluster and *LDLR*) and the environment (diet, the microbiome and medications). Our results point toward metabolic convergence for sleep disturbances and poor diet in autistic children, with implications for long-term wellbeing and quality of life.

## Methods

### Ethics approvals

All families provided informed consent to be included in this study. For the AAB, ethics approvals were as follows. At the New South Wales recruitment site, ethics approval was through the Sydney Children’s Hospital Network Human Research Ethics Committee (HREC) (approval number HREC/14/SCHN/269). At the Queensland recruitment sites, ethics approval was through the Mater Health Services HREC (approval number HREC/14/MHS/212) and The University of Queensland (approval number 2014001079). At the Victoria recruitment site, ethics approval was through La Trobe University (approval number HEC16/104). At the Western Australia site, ethics approval was through the Princess Margaret Hospital for Children (approval number 2014029EP) and The University of Western Australia (approval number RA/4/1/8184). For the QTAB Project, ethics approval was through the Children’s Health Queensland HREC (approval number HREC/16/QRCH/270) and The University of Queensland (approval number 2016001784/ HREC/16/QRCH/270).

### Statistics and reproducibility

No method was used to predetermine sample size. As a data-driven project, our aim was instead to maximize the sample size within budget constraints. Randomization was performed within the lipidomics data acquisition process to mitigate batch effects. As a data-driven, exploratory project, there were no interventions to randomize to. The investigators were not blinded to allocation during the clinical and survey assessment relating to phenotypic data for the AAB. However, investigators were blinded in the lipidomics sample preparation and data acquisition process. An in-depth explanation of sample exclusion by analysis is provided in the ‘Study participants’ and ‘Approach to outliers’ sections below.

### Data

#### Study participants

This study included a total of 765 participants (predominantly recruited from the AAB, but with a smaller number from the QTAB Project): 485 with a diagnosis of ASD, 160 undiagnosed siblings without recorded ASD diagnosis and 120 unrelated undiagnosed children (96 from the AAB and 24 from QTAB) (Fig. 1).

Seven statistical outlier samples (six ASD and one UNR) were identified that met two criteria: (1) exceeding the 99th percentile of an extremeness score, calculated for each individual and representing the number of *z*-transformed metabolites that were >3 standard deviations from the mean; and (2) a distance to the origin in PCA in the 99th percentile of the distribution. These outlier samples were excluded from all analyses except the outlier analysis (see the section ‘Lipidome outliers’), as four (potentially) had biological rather than technical explanations (for example, none were observed to have batch processing hemolysis) (see the section ‘Approach to outliers’). We also excluded one participant with Smith–Magenis syndrome within the UNR group. Overall, in the main analyses (that is, excluding the ‘Lipidome outliers’ section), there were a total of 758 participants (479 ASD, 160 SIB and 119 UNR). Supplementary Table 1 summarizes the data in this sample across the three groups.

We also identified a subset of *n* = 64 participants in the ASD group for whom the samples had been stored for a longer period of time than other samples (≥2,500 days; storage outliers; Supplementary Figs. 1 and 2). Preliminary analyses suggested an important relationship between storage duration and lipid profiles (specifically, an increase in oxidized species). Since these storage outliers all had ASD diagnoses, they were excluded from analyses of ASD diagnosis (leaving *n* = 694 participants). However, they were retained for analyses of other traits (for example, age, IQ/DQ and CSHQ total score) after sensitivity analyses confirmed that they did not significantly affect conclusions. We note that these older samples tended to have less phenotypic data collected (*n* = 17 with IQ/DQ, *n* = 59 with CSHQ total score) and none had provided dietary data, so they were already excluded from many analyses for these reasons.

A total of *n* = 12 participants had plasma samples that were visibly fatty. These were included in all analyses but were also examined as a group of interest for whom there was an a priori expectation of a different lipid profile.

#### Lipidomics

Lipidomics was performed as described previously^51^ with modifications. Analysis of plasma extracts was performed on an Agilent 6495C Triple Quadrupole mass spectrometer with an Agilent 1290 series high-performance liquid chromatography system and a single ZORBAX Eclipse Plus C18 column (2.1 × 100 mm; 1.8 µm; Agilent) with the thermostat set at 45 °C. Samples were randomized to processing batch and injection order.

Mass spectrometry analysis was performed in both positive and negative ion mode with dynamic scheduled multiple reaction monitoring. The running solvent consisted of solvent A (50% H_2_O, 30% acetonitrile and 20% isopropanol (vol/vol/vol) containing 10 mM ammonium formate and 5 μM medronic acid) and solvent B (1% H_2_O, 9% acetonitrile and 90% isopropanol (vol/vol/vol) containing 10 mM ammonium formate).

The following mass spectrometer conditions were used: gas temperature = 150 °C; gas flow rate = 17 l min^−1^; nebulizer = 20 psi; sheath gas temperature = 200 °C; capillary voltage = 3,500 V; and sheath gas flow = 10 l min^−1^. Isolation widths for Q1 and Q3 were set to unit resolution (0.7 atomic mass units).

For the chromatography, we used a stepped linear gradient with a 16 min cycle time per sample and a 1 µl sample injection. The sample analytical gradient started with a flow rate of 0.4 ml min^−1^ at 15% B and increased to 50% B over 2.5 min, then to 57% over 0.1 min, 70% over 6.4 min, 93% over 0.1 min and 96% over 1.9 min, and then ramped to 100% over 0.1 min. The solvent was then held at 100% B for 0.9 min (total = 12.0 min). Equilibration was started as follows. The solvent was decreased from 100% B to 15% B over 0.2 min and held for a final run time of 16 min. We included three quality control sample types: pooled quality control, technical quality control and NIST 1950 SRM samples. Pooled quality control samples were included at a rate of 1:20 per sample during the lipid extraction process. These provided an indicator for variance across both the extraction and mass spectrometry analysis. The technical quality control samples were a pre-extracted set of pooled samples identical in composition. These were injected from an independent vial at a rate of 1:20 injections to measure variation across the mass spectrometry run. NIST 1950 SRM is a commonly used standardized reference material for aligning the lipidomic data between different studies and was injected at a rate of 1 per 40 samples. As an indicator of sample variability, we calculated percentage coefficients of variation for the three quality control sample types: technical quality control (6.7%), pooled quality control (7.6%) and the standardized NIST 1950 SRM (7.4%). These quality control types have been described previously^51^.

MassHunter Quantitative B08 was used to quantify lipid concentrations from mass spectrometry data. Relative quantification of lipid species was determined by comparison with the relevant internal standard. Lipid class total concentrations were calculated as the sum of individual lipid species concentrations, except in the case of classes triglycerides (TG) and akyldiacylglycerols (TG(O)), for which we measured both neutral loss and single ion monitoring (SIM) peaks and subsequently used the more numerous but less structurally resolved SIM species concentrations for summation purposes when examining lipid totals.

Blood samples are difficult to collect in children, and specifically those on the autism spectrum. To improve recruitment, a standardized protocol for collection time of day was not enforced, nor were participants required to fast before sample donation.

Overall, the lipidomics assay panel quantified 793 species (out of 825 in the full Baker Institute panel at the time of data acquisition) grouped into 41 lipid classes. The lipid species are the most granular level of lipid ontology and make up the lipid classes. There are also additional categories used in this ontology, including subclasses, features and domains, which are not strictly collapsed within each other. We also inferred clinical lipid levels with total cholesterol, calculated as the sum of the free cholesterol lipid species and the cholesteryl ester lipid class. Triglyceride levels were approximated using the triglyceride (SIM) lipid class measure. Note that the lipidome includes highly correlated data at the species and class hierarchies (Extended Data Fig. 1).

We excluded *n* = 10 lipid species for which plate processing batch and injection order for the generation of lipid profiles explained >10% of the variance in lipid concentration (in a linear model of lipid concentration regressed against these confounders). We also confirmed that these excluded lipids had negligible association with ASD diagnosis in a model of lipid concentration ~ ASD diagnosis (all explaining ≤1% of variance). Hence, after quality control, 783 lipid species remained.

#### Overview of metadata and covariates

We focused on three neurodevelopmental traits: (1) ASD diagnosis; (2) IQ/DQ composite score (which we obtained by aggregating composite scores from the fourth edition of the Wechsler Intelligence Scale for Children^52^ (for older children in the AAB), the age-corrected NIH Toolbox Cognitive Function composite score (which can be interpreted similarly to a full-scale score^53^; for the QTAB study) and the nonverbal developmental quotient score from the Mullen Scales of Early Learning^54^ (for younger children in the AAB) as a proxy for cognitive ability and developmental delay); and (3) sleep disturbances, measured using the CSHQ^55^ total sleep disturbance score (which captures sleep disturbances across eight subdomains: bedtime resistance, sleep onset delay, sleep duration, sleep anxiety, night waking, parasomnias, sleep-disordered breathing and daytime sleepiness). As benchmarking traits and positive controls, we also performed analyses on age, Tanner score (genital; representing pubertal stage) and BMI *z* score (calculated using the R zscorer package^56^, which draws on the World Health Organization growth chart references for age and sex).

Lipidome measures are molecular traits with both genetic and environmental influences, so lipidomic analysis requires careful consideration of covariates. For the neurodevelopmental traits, we generally considered demographic (age, age^2^ and sex) and batch variables (lipidomics batch and injection order and sample storage duration) and collection time (with cosine transformation to model periodicity) to be noise covariates (explanatory variables) that could affect lipid levels and were therefore adjusted for in most analyses. For the traits age and Tanner score (genital), we did not include age and age^2^ covariates. For BMI (*z* score), we did not include age, age^2^ and sex covariates, as these are accounted for within the population-normed *z* score.

We also examined the interplay of lipidomics data with PGSs for lipid, dietary and microbiome data as explanatory variables of interest. These datasets are described in more detail below.

#### PGSs

We obtained summary statistics for genome-wide-significant SNPs from a GWAS of *n* = 490 overlapping lipid traits investigated in the BHS^24^ (one GWAS per lipid species), which investigated a near-identical plasma lipid panel from the Baker Heart and Diabetes Institute Metabolomics Group. Briefly, their study identified 733 genomic regions with genome-wide-significant evidence (*P* ≤ 5 × 10^−8^) for association with lipid levels. Given the sample size of 6,057 individuals, this necessarily reflects that many associations had a large effect size. We used the genome-wide-significant loci to construct PGSs for *n* = 490 lipid traits. For each lipid species trait, we performed linkage disequilibrium clumping with default settings (variants within a 250 kilobase distance with *R*^2^ ≥ 0.10) to identify an independent set of SNPs, using the AAB/QTAB imputed genotypes as a linkage disequilibrium reference. With this set of SNPs, we generated PGSs with the beta statistics from the original GWAS in PLINK^57^ (version 1.90) using the --score function, which multiplies each SNP effect size by an individual’s allele dosage, then sums across all independent loci. We focused our analysis on European participants, whom we had identified in previous work^31^, so that ancestries were matched to the BHS GWAS dataset, which increased the predictive ability. We standardized the PGSs within the European dataset (including parents and children who provided SNP genotyping samples but did not have lipidomics data measured here) to have a mean of 0 and a standard deviation of 1.

#### Dietary data

Dietary data from the Australian Eating Survey (AES)^58,59^ were available for a subset of *n* = 264 children with lipidomics data, of whom 261 remained in the main lipidomics dataset (ASD = 123, SIB = 60 and UNR = 78) after excluding outliers. We focused on two forms of dietary data: (1) AES variables quantifying food groups such as proteins, fats (including subtotals of saturated, polyunsaturated and monounsaturated fats), cholesterol and total carbohydrates (including a subtotal for sugar); and (2) dietary profiles that were generated by performing compositionally aware PCA on data representing the percentage of energy from various food groups (that is, vegetables, fruit, meat, alternative proteins, grains, dairy, sweet drinks, packed snacks, confectionery, baked products, takeaway, condiments and fatty meats). To generate dietary profiles (referred to as dietary PCs), we applied a centered log-ratio transformation (to account for the compositional nature of the data) and then performed PCA. This data processing has previously been described within the AAB/QTAB dataset^50^, but principal components were regenerated here for the *n* = 264 individuals for whom there were both lipidomics and dietary data.

#### Microbiome data

Stool metagenomics data^50^ (including both taxonomic and functional count data) were available for a subset of *n* = 169 children in the AAB and QTAB cohorts (ASD = 90, SIB = 43 and UNR = 39) with matching lipidomics and dietary data (and after excluding outliers). We used metagenomics data (described previously^50^) that included species and multiple functional annotations; for the latter, we focused on the MetaCyc pathways as these had the most easily interpretable functional descriptions. In this analysis, we filtered for common microbiome features by focusing on microbiome features that were present in ten or more individuals in the subset of *n* = 169 individuals with overlapping lipidomics data. This resulted in *n* = 511 species and *n* = 764 MetaCyc pathways.

#### Medication data

Six self- or parent-reported medication/supplement categories were considered in this study: ADHD/behavioral, antipsychotics, antidepressants/anxiolytics, antiepileptics, sleep medications or supplements and fish oil/DHA supplements.

#### Batch and technical variables

We considered three batch and technical variables, reflecting lipidomics data generation (processing batch and injection order), storage duration (see the section ‘Approach to outliers’) and collection time of day (as many lipids exhibit a diurnal pattern and plasma samples were not donated using a fasting protocol; Supplementary Fig. 1).

To model periodicity in the collection time of day (*n* = 657 with available data), we applied the following transformation, dividing by 2,400 for simplicity as the data were provided in 24-h time.

$$\sin \left( {2\pi \times \frac{{{\rm{collection}}\,{\rm{time}}}}{{2,400}}} \right)$$

### Sex and gender reporting

This research included *n* = 500 boys and *n* = 265 girls. There were more males than females in this sample as ASD is diagnosed more frequently in boys and the majority of participants were in the ASD group. Our analyses focused on biological sex rather than gender (confirmed using previously analyzed genetic data^31^). We also statistically accounted for biological sex by including this as a covariate in all analyses except where specified (for example, sensitivity analyses without covariates). We performed analyses to investigate the relationships between sex and the lipidome (Fig. 2b for variance component analysis; Supplementary Table 10 for LWASs), as well as the relationship between sex and neurodevelopmental phenotypes (see the section ‘Overview of the dataset’). In Supplementary Table 1, we have provided the sex breakdown in each of the ASD, SIB and UNR groups. Individual-level data are available by application to the AAB and QTAB (see the ‘Data availability’ statement). We did not perform additional sex-stratified analyses as this would result in an underpowered analysis while also increasing the multiple testing burden.

### Approach to outliers

The *n* = 7 statistical outliers (see the section ‘Study participants’) were excluded for all analyses except for those within the ‘Lipidome outliers’ section, in which they were specifically interrogated for potential biological explanations. The rationale for this was that including these outliers when investigating mean group differences (that is, OREML analyses, LWASs, PGSs and trait lipidome associations) could bias the results.

For the analyses investigating mean group differences related to ASD diagnosis, we excluded *n* = 64 storage duration outliers, defined as samples with a storage duration of ≥2,500 days. This was motivated by our observation that the ASD OREML analysis without covariates (nocov; Fig. 2b) had a significantly higher *R*^2^ value than the analysis including demographic and batch variables (covdemo). Closer inspection of the data revealed that storage time was confounded with ASD diagnostic status (Supplementary Fig. 2), which was because the AAB initially recruited only children with an ASD diagnosis (who typically have co-occurring intellectual disability and sleep disturbances) before expanding to include undiagnosed children. Furthermore, we found that lipid species and classes that are known to correlate with sample degradation (for example, the LPC(O-18:0)/PC(O-18:0/20:4) ratio and the oxidized species class) were associated with ASD diagnostic status. To ameliorate this confounding effect, we excluded individuals with a sample storage of ≥2,500 days in the ASD analyses. However, other phenotypes did not suffer the same imbalance in sample storage time (Supplementary Fig. 2) and we confirmed in sensitivity analyses that excluding these individuals did not significantly affect our conclusions; hence, there was no need to exclude storage time outliers in these other analyses. We also note that samples from individuals with matched dietary data had shorter storage times, so including dietary data as covariates effectively excluded the storage time outliers.

To additionally decrease the effect of outliers on our results, we performed a rank-based inverse normal transformation of the lipidomics data in the OREML, LWAS and trait lipidome analyses.

### Statistical analysis

#### ASD diagnosis and inferred clinical lipids

To determine whether inferred lipidome cholesterol explained significant variance in ASD diagnosis beyond the contributions of potential confounding variables, we used a likelihood ratio test to compare the following two models:

$$\begin{array}{l}{\rm{Model}}\,0:{\rm{diagnosis}}\sim {\rm{age}} + {\rm{age}}^2 + {\rm{sex}} + {\rm{batch}}_{{\rm{lipidomics}}} \\+ {\rm{injection}}\,{\rm{order}}\end{array}$$

$$\begin{array}{ccccc}\\ {\rm{Model}}\,1:{\rm{diagnosis}}\sim {\rm{age}} + {\rm{age}}^2 + {\rm{sex}} + {\rm{batch}}_{{\rm{lipidomics}}} \\+ {\rm{injection}}\,{\rm{order}} + {\rm{cholesterol}}_{{\rm{lipidome}}}\\ \end{array}$$

We then performed sensitivity analyses to investigate lifestyle and clinical factors that could mediate the relationship between ASD diagnosis and lower plasma lipidome cholesterol levels, and their potential relationship with other lifestyle and clinical variables.

First, we examined the effect of dietary cholesterol intake among *n* = 261 participants for whom both datasets were available (about one-third of the total lipidomics dataset). We generated three logistic regression models to assess the conditional associations between diagnosis and dietary versus lipidome cholesterol:

$${\rm{Model}}\,0:{\rm{diagnosis}}\sim {\rm{age}} + {\rm{age}}^2 + {\rm{sex}} + {\rm{injection}}\,{\rm{order}}$$

$$\begin{array}{l}{\rm{Model}}\,1:{\rm{diagnosis}}\sim {\rm{age}} + {\rm{age}}^2 + {\rm{sex}}\\ + {\rm{injection}}\,{\rm{order}} + {\rm{cholesterol}}_{{\rm{dietary}}}\end{array}$$

$$\begin{array}{l}{\rm{Model}}\,2:{\rm{diagnosis}}\sim {\rm{age}} + {\rm{age}}^2 + {\rm{sex}} + {\rm{injection}}\,{\rm{order}} \\ + {\rm{cholesterol}}_{{\rm{dietary}}} + {\rm{cholesterol}}_{{\rm{lipidome}}} \end{array}$$

Second, we performed a sensitivity analysis excluding participants with current or previous self- or parent-reported antipsychotic or ADHD/behavioral medication usage (*n* = 630 included in the analysis, for whom self-report was available on *n* = 468, of whom *n* = 35 self-reported antipsychotic usage and *n* = 99 reported medications prescribed for ADHD or challenging behavior). We repeated the likelihood ratio test comparing:

$${\rm{Model}}\,0:{\rm{diagnosis}}\sim {\rm{age}} + {\rm{age}}^2 + {\rm{sex}} + {\rm{injection}}\,{\rm{order}}$$

$$\begin{array}{l}{\rm{Model}}\,1:{\rm{diagnosis}}\,\sim \,{\rm{age}} + {\rm{age}}^2 + {\rm{sex}} \\+ {\rm{injection}}\,{\rm{order}} + {\rm{cholesterol}}_{{\rm{lipdome}}}\end{array}$$

Third, we excluded participants with self- or parent-reported fish oil or DHA use and repeated the above likelihood ratio test, leaving *n* = 626.

#### Variance component analysis (per trait)

We estimated the proportion of variance in clinical phenotypes associated with the lipidomics data using the OREML method implemented in the OSCA package^22^ (version 0.46).

Using a mixed linear model fitting all lipids as random effects:

$${\bf{y}} = {\bf{C}}{\bf{\upbeta}} + {\bf{Wu}} + {\bf{e}}$$

where **Cβ** represents fixed-effect covariates, **W** represents a matrix of standardized lipidome measures for all samples, and the random effects ${\mathbf{u}}\sim N\left( {0,{\mathbf{I}}\sigma _u^2} \right)$ and ${\mathbf{e}}\sim N\left( {0,{\mathbf{I}}\sigma _e^2} \right)$ represent the joint effects of all probes on the phenotype and error, respectively. The variance–covariance matrix for *y* is below, and OREML solves for:

$${{{\mathrm{var}}}}\left( {\bf{y}} \right) = {\bf{WW}}^\prime \sigma _u^2 + {\bf{I}}\sigma _e^2 = {\bf{A}}_{{{\mathrm{o}}}}\sigma _{\rm{o}}^2 + {\bf{I}}\sigma _e^2$$

where $\sigma _o^2$ represents the variance explained by the omics dataset (the value of interest) and **A**_*o*_ is the omic data-based relationship matrix, where diagonal elements tend toward 1 and off-diagonal elements represent the pairwise correlation of lipid measures between two individuals.

We applied a rank-based inverse normal transformation to each lipid measure and considered various sets of covariates: no covariates (nocov); the covariates age, age^2^ (except for when age, Tanner stage and BMI were taken as the clinical phenotypes), sex (except when the BMI *z* score and sex were taken as the clinical phenotypes), batch, injection order and storage duration (covdemog); and, as sensitivity analyses, adding either dietary profiles (covdemogdiet) or collection time of day (covdemogtime). We considered three categories of dietary variables: dietary profiles (dietary PC1–PC3, from PCA of per-food-group percentage energy contribution; see above and described previously^50^); macronutrients (cholesterol, protein, fats, sugars and carbohydrate); and dietary diversity (measured using the Shannon index applied to the AES food-level data). For the analysis of dietary profiles, we included as covariates age, age^2^, sex, batch, injection order and storage duration. For the other dietary variables, we only included the covariates sex, batch, injection order and storage duration and instead performed a sensitivity analysis including energy intake as a covariate (covdemogenergy), reasoning that energy intake is strongly correlated with age and is more relevant to dietary data.

For all traits, we excluded the *n* = 7 participants identified as statistical outliers in the lipidomics data quality control. For the ASD diagnosis analysis, we additionally excluded the *n* = 64 storage duration outliers. We tested the effect of excluding the *n* = 64 storage outliers before and after performing the inverse normal transformation to the lipidomics dataset, finding negligible difference. Furthermore, excluding these outliers had minimal effects on the results for all traits other than ASD diagnosis.

We performed sensitivity analyses accounting for the time of sample collection and dietary data, as these variables are likely to be important but were only available in a subset of participants. We were unable to perform a sensitivity analysis including total cholesterol and total triglyceride levels due to collinearity between these covariates.

Our multiple testing strategy is described in the section ‘Multiple testing strategy’. Briefly, we took a phenome-wide approach, using Bonferroni–Hochberg correction across all 18 traits (neuro/developmental and dietary) in the primary analysis.

#### LSEA

Lipid set annotations were provided by the Baker Heart and Diabetes Institute, corresponding to domain, class, subclass and feature. We used these annotations in correlated random variable LSEA (C.G. et al, in preparation), which tests for enrichment of pathways across the entire lipidome while accounting for the correlation structure between features. We first calculated association *t*-statistics of lipid concentrations against outcomes. Covariate-adjusted lipid correlations were calculated by regressing covariates (age, age^2^, sex, batch, injection order and storage duration; for all analyses except those excluding age and age^2^ when age, Tanner stage or BMI (*z* score) were the traits of interest and those excluding sex when BMI (*z* score) or sex were the traits of interest) from the trait of interest against the adjusted lipid concentrations and generating a correlation matrix from the residuals. For each lipid set, the LSEA statistic was calculated as the sum of *t*-statistics, adjusting for lipid correlation (taking as the denominator the square root of the sum of the adjusted correlation matrix specific to the lipid set). The *P* value was approximated as the chi-squared of the LSEA statistic squared with one degree of freedom. Under the null hypothesis, the squared LSEA statistic follows the *F* statistic, which tends to the chi-squared statistic with large sample size.

#### LWASs

We performed LWASs using the linear/logistic models implemented in OSCA^22^ (version 0.46). We performed these analyses for ASD diagnosis, IQ/DQ, sleep disturbances, age, Tanner stage and BMI, using the same transformations, subsets of participants and covariate combinations (demographic and batch) as in the OREML analysis.

As sensitivity analyses to control for false positives, we performed linear or logistic models with and without covariates (age, age^2^, sex, batch, injection order and storage duration for all traits; the exceptions were the following: excluding age and age^2^ as covariates for age, Tanner and BMI and excluding sex as a covariate for BMI and sex). We had also investigated OSCA-MOA (mixed linear model-based omic association)—a mixed model method that accounts for the correlation structure between participants—but found that the median test statistic was lower than expected under a null hypothesis, implying that MOA was susceptible to false negatives when applied to these highly correlated lipidomics data. We also performed sensitivity analyses adjusting for clinical lipids, to determine lipidome changes independent of total quantities of lipoproteins. For these analyses, we included additional covariates representing inferred total cholesterol (the sum of free cholesterol + total cholesteryl ester lipids) and total triglyceride levels (total triglyceride (SIM)). We performed an additional sensitivity analysis adjusting for collection time of day (see the section ‘Batch and technical variables’) for the individuals for whom these data were available (*n* = 657).

We adjusted for multiple testing in a manner that was sensitive to the strong correlational structure within the lipidomics dataset (see the section ‘Multiple testing strategy’). Instead of applying Bonferroni correction (which would be overly conservative), we applied PCA to the lipidomics datasets (by lipid class and by lipid species, both with inverse normal transformation) and identified the number of principal components that explained 99% of the variance in the data as the effective number of variables^60–63^. For our multiple testing threshold, we divided *P* = 0.05 by the effective number of variables (*n* = 32 for lipid classes versus a total of *n* = 41, and *n* = 302 for lipid species versus a total of *n* = 783), analogous to Bonferroni correction. In addition, we performed sensitivity analyses, taking as the multiple testing denominator the number of principal components explaining 95% of the variance (*n* = 23 for lipid classes and *n* = 129 for lipid species). Multiple testing correction was applied per trait lipidome analysis (for example, separately for each combination of traits and lipid class/species analyses).

For each trait, we then conducted a linear model analysis, fitting simultaneously all significantly associated lipids and applying post-hoc backwards stepwise regression (using the R package MASS, which optimizes the model based on the Akaike information criterion) to identify the most associated set of lipids accounting for the correlation between them.

We additionally used the lipid pathway annotations to label the LWAS significant hits.

#### GWAS replication

For a handful of lipid traits, we ran GWAS to examine whether genetic control was similar in this pediatric dataset to that of adults from the BHS lipidome GWAS^24^. We used GCTA (version 1.93.2 beta) MLMA^64^, using inverse normal-transformed lipidome data for European participants from AAB/QTAB and including age, age^2^, sex, batch, injection order, storage duration and 20 genotyping principal components representing population stratification.

The purpose of this analysis was to replicate the large genetic effects on lipid levels identified in the BHS GWAS^24^ in the European subset of the AAB/QTAB cohort, which is pediatric and one-tenth of the size (*n* = 646).

#### Dissection of variance in lipid concentration

Focusing on the lipid species that were significantly associated with clinical traits (and for which GWAS summary statistics were also available), we dissected the variance in lipid concentrations explained by genetic and clinical data.

We quantified the variance explained in each lipid to understand the relative contributions of covariates to lipid traits. This was achieved by calculating *R*^2^ in a linear regression model. We performed ANOVA of the following models, quantifying *R*^2^ for each term:

1. Lipid trait ~ PGS + age, age^2^ and sex + group
2. Lipid trait ~ PGS + age, age^2^ and sex + dietary variables (protein, saturated fats, polyunsaturated fats, monounsaturated fats, cholesterol and sugars) + group
3. Lipid trait ~ PGS + age, age^2^ and sex + batch variables + time of day + group

#### SMR

We applied the SMR (version 1.03) method^65^ to external GWAS summary statistics to test for associations between: (1) two lipid species identified in our LWAS analyses (PC(O-18:0/20:4 and PE(P-19:0/20:4)(b))^24^ and their associated neurodevelopmental traits (IQ^25^ and sleep duration^26^); (2) the same two lipidome traits^24^ and gene expression; and (3) gene expression and the neurodevelopmental traits. For the lipid trait analyses, we selected rs99780 as the instrument to test the IQ/PC(O-18:0/20:4) association and rs102274 for the sleep disturbances/PE(P-19:0/20:4)(b) association. To investigate whether specific genes mediated the lipid trait associations, we performed SMR investigating gene–lipid and gene–trait associations using eQTLGen summary statistics^66^ for genes in the window of chr11:61050000–62200000, using the same SNP instruments as for the lipid trait SMR. For each analysis, the HEIDI test^65^ was used to determine whether putative relationships between traits were due to genetic linkage, as opposed to pleiotropy or causality.

#### Lipid PGS prediction in AAB/QTAB and ABCD

For the LWAS hits, we also investigated whether genetically predicted lipid levels (that is, lipid PGSs) could predict the neurodevelopmental traits.

Within the AAB/QTAB dataset, we did not find significant associations between the neurodevelopmental traits and lipid PGSs, but the direction of effect was consistent for 17 out of 24 lipid species.

As a replication analysis to support our main results, we turned to the ABCD dataset to genetically predict levels of the two lipids used in the SMR analysis: PC(O-18:0/20:4) and PE(P-19:0/20:4)(b). As equivalent traits for IQ and sleep disturbances in the AAB/QTAB dataset, we used the NIH Toolbox Total Cognition Score and Sleep Disturbance Scale Total Sleep Score, respectively, among 4,952 European participants.

In this analysis, we regenerated PGSs by filtering the full BHS GWAS summary statistics for the PC(O-18:0/20:4) and PE(P-19:0/20:4)(b) lipid species to SNPs in the AAB/QTAB and ABCD datasets, then applying the same *P* value clumping and thresholding method, as described in the main analysis. As the *P* ≤ 5 × 10^−8^ threshold only yielded one SNP for prediction, we selected the *P* value threshold that explained the greatest proportion of variance in the lipid trait within the AAB/QTAB dataset. For PC(O-18:0/20:4), we took the threshold *P* ≤ 5 × 10^−6^ (AAB/QTAB dataset: *R*^2^ = 4.6% and *P* = 3.7 × 10^−8^) and for PE(P-19:0/20:4)(b), we selected *P* ≤ 5 × 10^−8^ (AAB/QTAB dataset: *R*^2^ = 1.5% and *P* = 1.6 × 10^−3^).

In the ABCD dataset, we regressed IQ against the PC(O-18:0/20:4) PGS using a linear model and regressed sleep disturbances against the PE(P-19:0/20:4)(b) PGS using a gamma log-link function, including covariates for age, sex, genotyping principal components and parental socioeconomic status.

#### Individual-level TWAS in AAB/QTAB and ABCD

We performed an individual-level TWAS analysis, which involved summing TWAS weights to predict gene expression in the *FADS* gene cluster where there was individual-level genotyping data. The rationale for this analysis was that both lipids PC(O-18:0/20:4) and PE(P-19:0/20:4)(b) have a highly oligogenic genetic architecture (at least in adult datasets^24^); hence, TWASs can help to fine map causal genes underlying these lipids, and may provide supporting evidence for lipid–neurodevelopmental trait associations.

First, in the AAB/QTAB dataset, we confirmed that predicted *FADS* cluster gene expression corresponded with lipid levels of PC(O-18:0/20:4) and PE(P-19:0/20:4)(b). We performed an ANOVA analysis (lipid concentration ~ age + age^2^ + sex + batch + injection order + TWAS estimate + trait) to quantify the variance associated with genetically predicted gene expression, conditioning on covariates. As there was differing availability of genes between TWAS weight datasets, we used TWAS Elastic Net weights from ref. ^67^ for *FADS1* (generated using Elastic Net weights from the Young Finns Study; 21 non-zero SNPs in the AAB/QTAB dataset), *FADS2* (Netherlands Twin Registry; 19 SNPs with Elastic Net weights) and *TMEM258* (GTEx version 7; *n* = 6 SNPs). For prefrontal cortex prediction, we used weights generated from PsychENCODE^68^, from which only *FADS1* (26/36 SNPs in the AAB/QTAB dataset, of which 17 had matching alleles) and *TMEM258* (6/11 SNPs available) were available. To genetically predict transcription levels, we multiplied TWAS weights by allele dosages for Europeans in the AAB/QTAB and ABCD datasets and then took their sum.

Given that the AAB/QTAB dataset was relatively small (*n* = 646 Europeans), we turned to the ABCD dataset (*n* = 4,592 Europeans) to test for associations between genetically predicted *FADS* cluster gene expression and clinical phenotypes that were proxies for IQ (NIH Toolbox Total Cognition Score) and sleep disturbances (Sleep Disturbance Scale Total Sleep Score).

#### LWAS hits and dietary and microbiome variables

To reduce dimensionality, for each neurodevelopmental trait (ASD diagnosis, IQ/DQ and sleep disturbances), we performed PCA on the LWAS species-level associations, referring to PC1 as the lipidome profile for that trait. We used linear models to examine relationships between these lipidome profiles, dietary principal components and measures of the focal traits.

Separately, we investigated the relationships between the three trait lipidome profiles and microbiome species and MetaCyc pathways. We performed analyses in ANCOM version 2.1, taking LWAS PC1 as the dependent variable and including the covariates age, age^2^, sex, batch, injection order and storage duration. In ANCOM version 2.1, features passing the detection threshold ≥0.7 correspond to significant associations, although a detection threshold of ≥0.6 may also be used. As a sensitivity analysis, we investigated the effect of including dietary principal components as additional covariates.

We used ANOVA to determine the proportion of variance in each neurodevelopmental trait’s lipidome profile that could be attributed to each explanatory variable (in the following order): age + age^2^ + sex + batch (batch number, injection order and storage duration) + medications associated with that trait lipidome profile + associated dietary principal components + associated microbiome species or MetaCyc pathways determined using the nominally significant associations from ANCOM version 2.1 (detection threshold ≥ 0.6).

#### Interplay of neurodevelopmental traits and the lipidome

We compared two ANOVA models to explore the association between ASD and the ASD lipidome profile, with and without conditioning on sleep disturbances:

$$\begin{array}{l} {\rm{Lipidome}}\,{\rm{profile}}_{{\rm{ASD}}}\sim {\rm{age}} + {\rm{sex}} + {\rm{batch}}_{{\rm{plate}}} + {\rm{batch}}_{{\rm{injection}}\,{\rm{order}}} \\ + {\rm{collection}}\,{\rm{time}}_{{\rm{cos}}\,{\rm{transform}}} + {\rm{storage}}\,{\rm{time}} + {\rm{ASD}} \end{array}$$

$$\begin{array}{l} {\rm{Lipidome}}\,{\rm{profile}}_{{\rm{ASD}}}\sim {\rm{age}} + {\rm{sex}} + {\rm{batch}}_{{\rm{plate}}} + {\rm{batch}}_{{\rm{injection}}\,{\rm{order}}} \\ + {\rm{collection}}\,{\rm{time}}_{{\rm{cos}}\,{\rm{transform}}} + {\rm{storage}}\,{\rm{time}} + {\rm{sleep}} + {\rm{ASD}} \end{array}$$

We performed a similar analysis for sleep disturbances, this time conditioning on ASD diagnosis:

$$\begin{array}{l} {\rm{Lipidome}}\,{\rm{profile}}_{{\rm{sleep}}}\sim {\rm{age}} + {\rm{sex}} + {\rm{batch}}_{{\rm{plate}}} + {\rm{batch}}_{{\rm{injection}}\,{\rm{order}}} \\ + {\rm{collection}}\,{\rm{time}}_{{\rm{cos}}\,{\rm{transform}}} + {\rm{storage}}\,{\rm{time}} + {\rm{sleep}} \end{array}$$

$$\begin{array}{l} {\rm{Lipidome}}\,{\rm{profile}}_{{\rm{sleep}}}\sim {\rm{age}} + {\rm{sex}} + {\rm{batch}}_{{\rm{plate}}} + {\rm{batch}}_{{\rm{injection}}\,{\rm{order}}} \\ + {\rm{collection}}\,{\rm{time}}_{{\rm{cos}}\,{\rm{transform}}} + {\rm{storage}}\,{\rm{time}} + {\rm{ASD}} + {\rm{sleep}}\end{array}$$

To investigate the relationship between VABS-II composite score, IQ/DQ and sleep disturbances and their associated lipidome profiles (note that the VABS-II was only assessed within the ASD group), we performed the following ANOVA models and assessed whether the trait-associated lipidome profile explained additional variance in the VABS-II composite score beyond the neurodevelopmental trait alone:

$${\rm{VABS}}_{{\rm{composite}}}\sim {\rm{IQ}}/{\rm{DQ}} + {\rm{lipidome}}\,{\rm{profile}}_{{\rm{IQ}}/{\rm{DQ}}}$$

$${\rm{VABS}}_{{\rm{composite}}}\sim {\rm{sleep}} + {\rm{lipidome}}\,{\rm{profile}}_{{\rm{sleep}}}$$

#### Group differences in variance

We tested for differences in variance between groups (ASD versus non-ASD) using Levene’s test. We accounted for covariates (age, sex, batch, injection order and storage duration) by regressing these effects out from each lipid concentration measure and taking the residuals. For this analysis, we included the outliers that had been identified using the quality control pipeline, which we justified due to finding that many of these outliers had a biological explanation. However, we excluded the *n* = 64 participants who were storage duration outliers, as: (1) storage duration was considered to be an undesirable batch effect; and (2) all of these outliers were in the ASD group, meaning that they may bias the group comparison. Our multiple testing strategy for this analysis is described in the section ‘Multiple testing strategy’.

### Multiple testing strategy

#### Approach when considering multiple lipids within one trait

Here we were investigating whether any specific lipids were associated with a given trait.

The null hypothesis was that no lipids were associated with trait *X*.

For the LWAS analysis at the lipid species level (Extended Data Figs. 2 and 3 and Supplementary Figs. 6–10) and the difference in variance analysis (Extended Data Fig. 10), we performed *n* = 783 tests for each trait. For the lipid class level, we performed *n* = 41 for each trait (Extended Data Fig. 3). Recognizing that the lipidome is highly correlated (Extended Data Fig. 1), we sought a multiple testing correction method that would account for this correlation (which conventional methods such as Bonferroni and Benjamini–Hochberg correction do not) while being sufficiently conservative. We took an approach that is used widely in the lipidomics field (for example, refs. ^62,63^) and that has historical precedence in gene-based analyses, which similarly contend with highly correlated data (for example, refs. ^60,61^). To this end, we:

1. Applied PCA to the lipidomics dataset
2. Estimated the effective number of independent lipids by taking the number of principal components accounting for >99% of variance in the dataset (*n* = 302 for the lipid species and *n* = 32 for the lipid classes)
3. Calculated the corrected *P* value threshold (essentially, Bonferroni correction for the number of independent lipids) as:

$$P_{{\rm{corrected}}} = \frac{{0.05}}{{{\rm{Effective}}\,{\rm{number}}\,{\rm{of}}\,{\rm{lipids}}}}$$

We applied multiple testing correction within each trait, as opposed to across all traits, consistent with current conventions in the lipidomics field (for example, ref. ^24^) and in other omics fields (for example, ref. ^69^). For the lipid species data, significance was declared at *P* < 1.66 × 10^−4^ and for the lipid classes it was declared at *P* < 1.56 × 10^−3^.

We also performed an analysis investigating the association of inferred clinical lipid levels (cholesterol and triglycerides) with three neurodevelopmental traits (ASD diagnosis, IQ/DQ and sleep disturbances) (Fig. 2a). Using the approach described above, our multiple testing strategy was to correct for two lipids per trait.

We also performed extensive sensitivity analyses assessing different combinations of covariates (Supplementary Figs. 6–10). We did not account for these sensitivity analyses in our multiple testing for two reasons: (1) these analyses were performed with the intention of understanding other variables that may contribute to the primary association(s); and (2) correcting for multiple testing would be unreasonably conservative given that the primary and sensitivity analyses are essentially the same (that is, the dependent variable is identical).

#### Approach when considering the lipidome as a whole (OREML)

Here we were taking a phenome-wide approach, testing the null hypothesis that no traits were significantly associated with the lipidome.

For the OREML analysis (Fig. 2b and Supplementary Table 3), we performed multiple testing correction across all tested traits (a total of 18 traits: ASD, IQ/DQ, sleep disturbances (CSHQ), age, Tanner score (genital), BMI (*z* score), sex, VABS-II adaptive motor domain, stool consistency (Bristol Stool Chart), protein, fats, carbohydrate, sugars, cholesterol, dietary PC1, PC2 and PC3 and dietary diversity. In this case, we applied a Benjamini–Hochberg correction to control the FDR across the 18 tests with the covdemog set of covariates (Fig. 2b; that is, the covariates age, age^2^ (except for when age, Tanner stage and BMI *z* score were taken as the clinical phenotypes), sex (except when BMI *z* score or sex were taken as the clinical phenotypes), batch, injection order and storage duration). We did not perform Bonferroni correction as these traits are correlated and this approach would be overly conservative. As data were not complete across all phenotypes, we were not able to perform the effective number of variables approach described above.

Again, we performed extensive sensitivity analyses to complement the main analyses and did not include these tests in multiple testing for the same reasons described above.

### Reporting summary

Further information on research design is available in the Nature Portfolio Reporting Summary linked to this article.

## Online content

Any methods, additional references, Nature Portfolio reporting summaries, source data, extended data, supplementary information, acknowledgements, peer review information; details of author contributions and competing interests; and statements of data and code availability are available at 10.1038/s41591-023-02271-1.

## Supplementary information

Supplementary Information Supplementary Figs. 1–20.

Reporting Summary

Supplementary Tables Supplementary Tables 1–25.

## Data Availability

The AAB datasets (lipidomics, SNP genotyping, stool metagenomics and phenotype data) supporting the conclusions of this article are available by application to the AAB within the Autism CRC (https://www.autismcrc.com.au/biobank). These data are not publicly available for ethical reasons. Applications are reviewed by a board including autistic people. The QTAB dataset used in these analyses is available with mediated access through The University of Queensland eSpace repository at 10.48610/dc9bf34. The ABCD dataset is available by application through the National Institute of Mental Health Data Archive (10.15154/1523041). BHS lipidomics GWAS results are available at https://metabolomics.baker.edu.au/.

## Code availability

Code is publicly available at https://github.com/cyap7/ASD_lipidomics_AAB_QTAB.
