# Supplementary material for: Interactions between the lipidome and genetic and environmental factors in autism
Source: Nat Med. 2023 Apr 19;29(4):936–49. doi: 10.1038/s41591-023-02271-1 (PMC10115648; doi:10.1038/s41591-023-02271-1)
Supplement: Supplementary file 1 — Supplementary Figs. 1–20. [file 41591_2023_2271_MOESM1_ESM.pdf]

nature medicine

Article https://doi.org/10.1038/s41591-023-02271-1

# Interactions between the lipidome and genetic and environmental factors in autism

In the format provided by the authors and unedited

# Table of contents


SUPPLEMENTARY FIGURE 1 2

SUPPLEMENTARY FIGURE 2 3

SUPPLEMENTARY FIGURE 3 4

SUPPLEMENTARY FIGURE 4 5

SUPPLEMENTARY FIGURE 5 6

SUPPLEMENTARY FIGURE 6 7

SUPPLEMENTARY FIGURE 7 8

SUPPLEMENTARY FIGURE 8 9

SUPPLEMENTARY FIGURE 9 10

SUPPLEMENTARY FIGURE 10 11

SUPPLEMENTARY FIGURE 11 12

SUPPLEMENTARY FIGURE 12 13

SUPPLEMENTARY FIGURE 13 14

SUPPLEMENTARY FIGURE 14 15

SUPPLEMENTARY FIGURE 15 16

SUPPLEMENTARY FIGURE 16 17

SUPPLEMENTARY FIGURE 17 18

SUPPLEMENTARY FIGURE 18 19

SUPPLEMENTARY FIGURE 19 20

SUPPLEMENTARY FIGURE 20 21

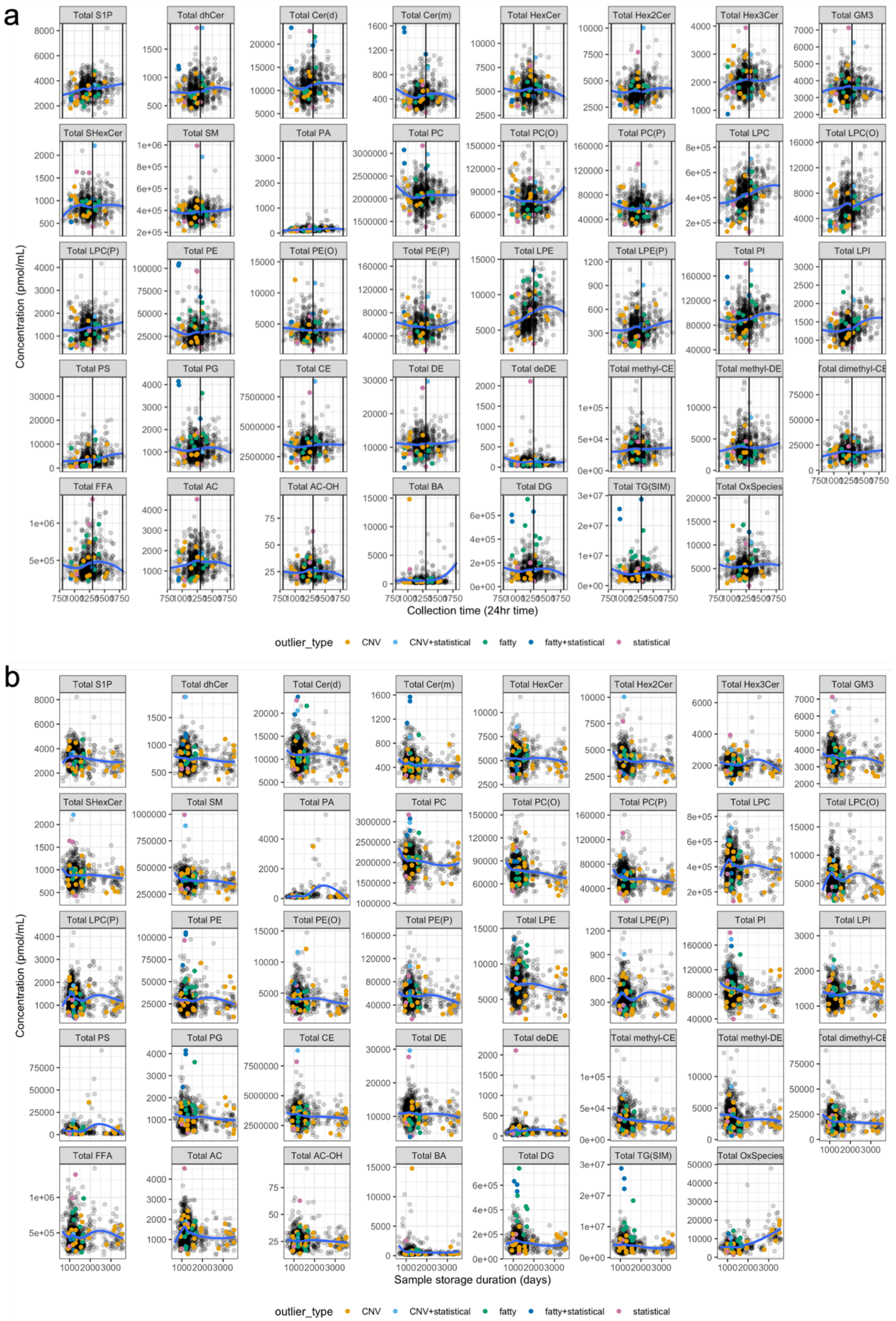


*Supplementary Figure 1: Relationship between a) collection time and b) storage duration and lipid class levels. Coloured points denote outliers: "CNV" denotes individuals identified to have a large CNV, "statistical" denotes outliers defined using lipidomics data QC (see Methods), "fatty", denotes samples that were observed to be visibly fatty. Note that all samples had storage duration data available, but not all samples had collection time data available (i.e., older samples)).*

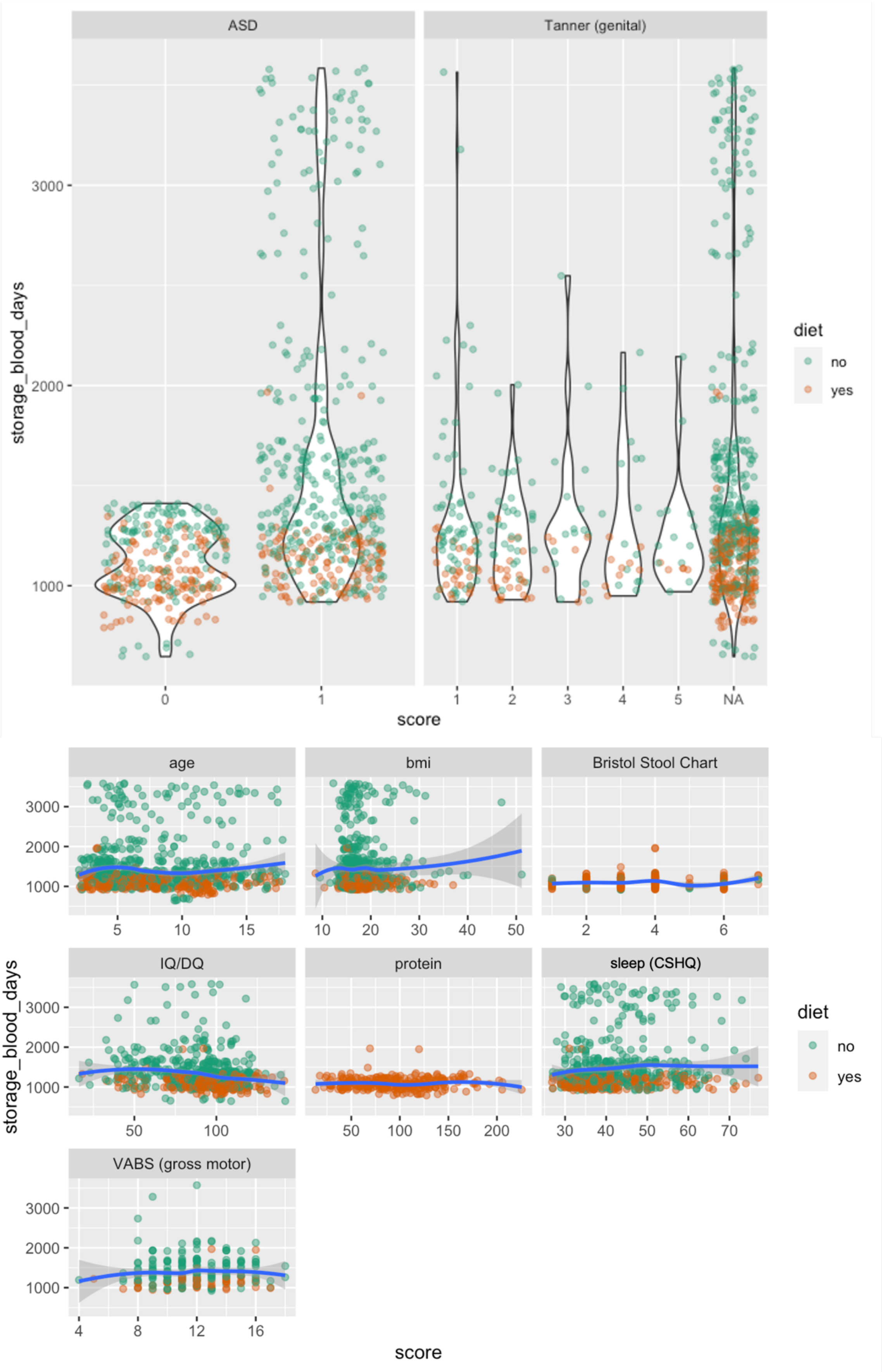


*Supplementary Figure 2: Relationship between storage time outliers and key phenotypes of interest, in relation to the decision to exclude individuals who had had samples stored for >2500 days in the ASD analyses, as storage time confounded ASD diagnostic status. However, other phenotypes did not suffer the same imbalance in sample storage time, so there was no need to exclude storage time outliers in these other analyses. Coloured points indicate individuals with available dietary variables, and these samples tended to have had shorter storage time. Hence, including dietary data as covariates effectively excluded the storage time outliers.*

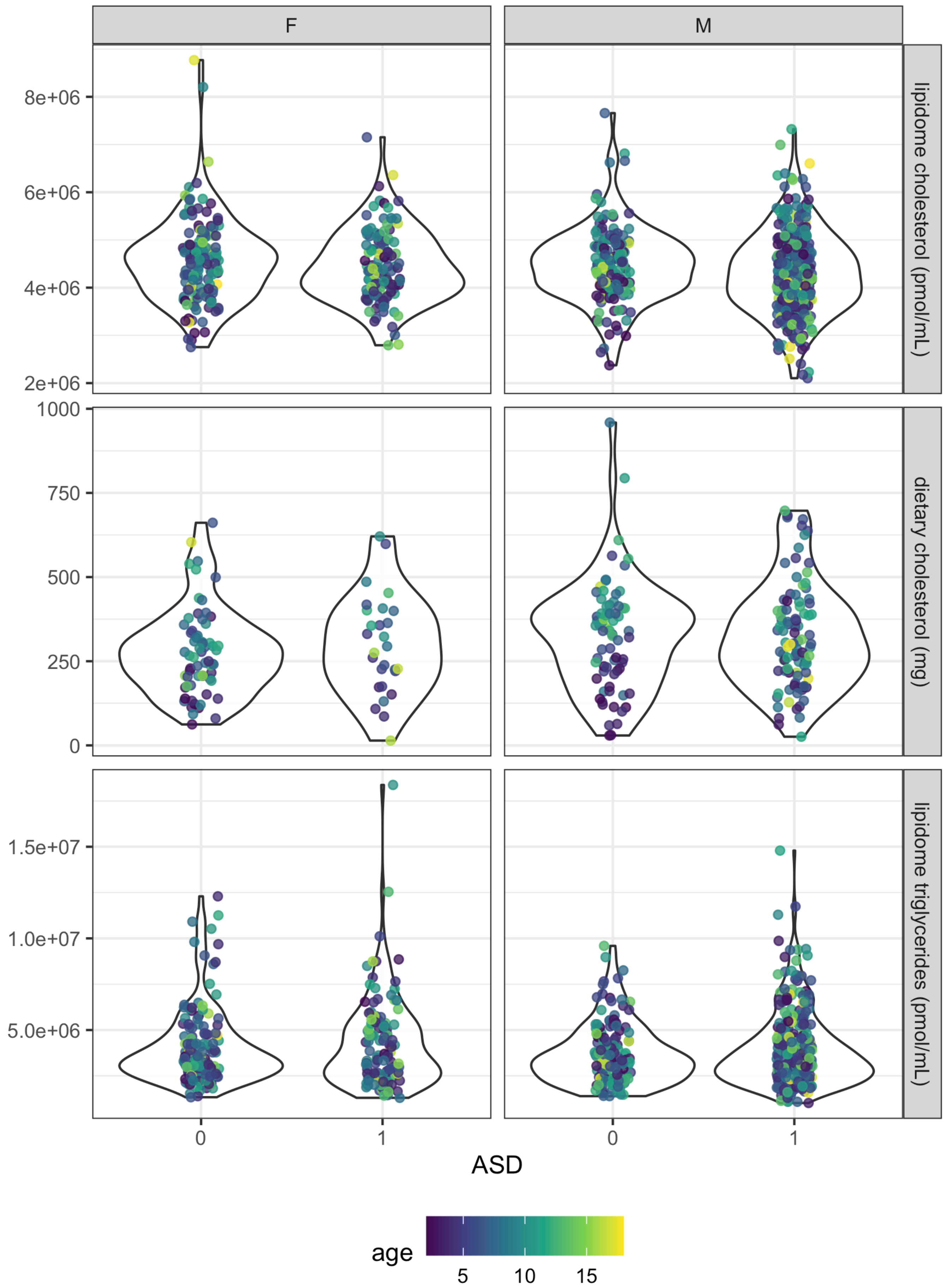


*Supplementary Figure 3: Relationship between ASD diagnosis and inferred lipidome cholesterol, dietary cholesterol, and inferred lipidome triglycerides, stratified by sex. ASD diagnosis is coded 1=ASD / 0=nonASD (siblings + unrelated undiagnosed children).*

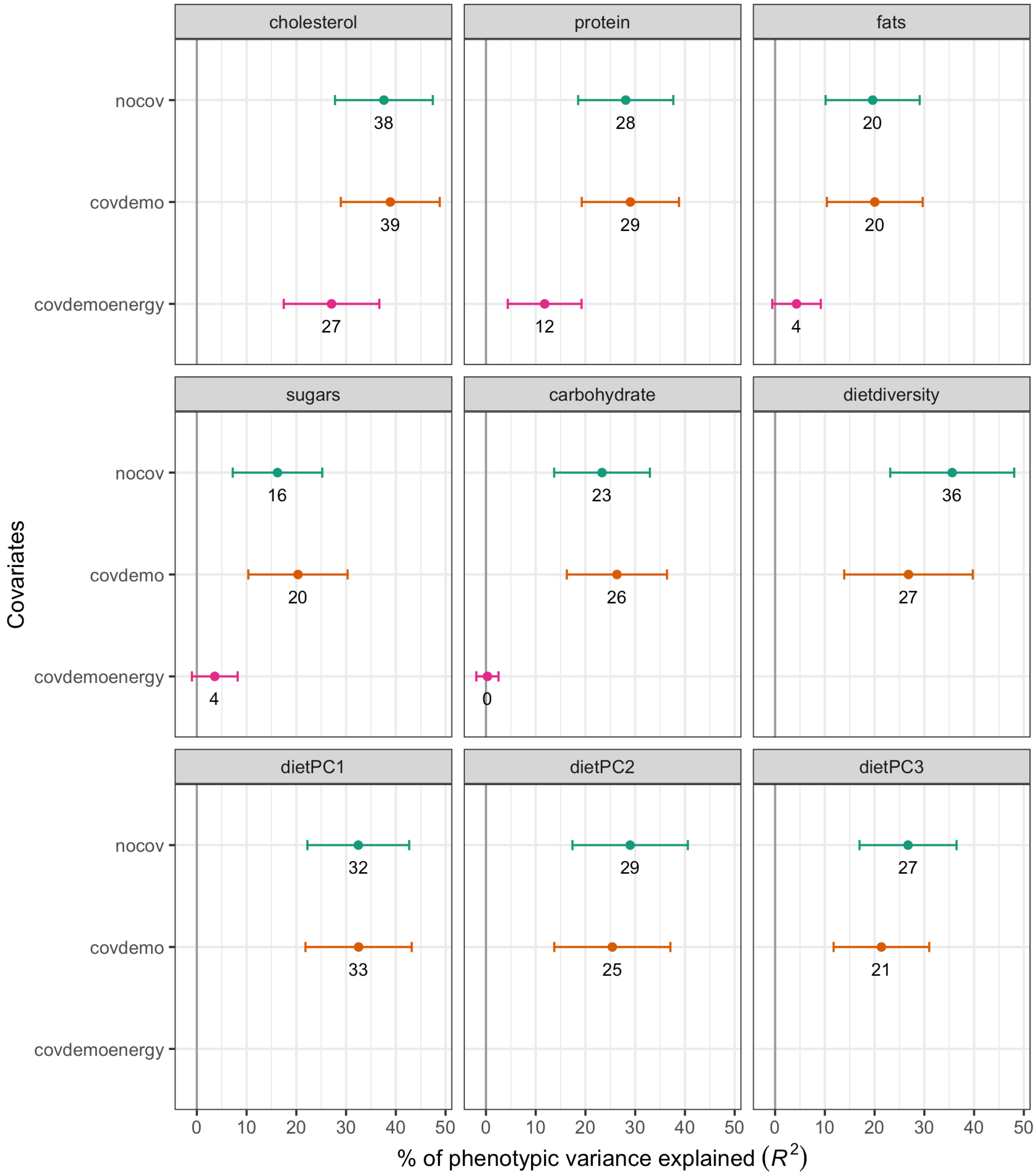


*Supplementary Figure 4: Additional results for dietary variables from the OREML variance component analysis. Point indicates % variance estimate and error bars denote standard error. Sensitivity analyses for different covariate combinations are shown: no covariates ("nocov"); demographic, batch and storage duration covariates ("covdemo"); demographic, batch, storage duration and dietary energy consumption ("covdemoenergy"). For the "covdemo" analysis (i.e., the primary analysis), the sample sizes were n=260 for all of the dietary traits, except for the dietary PC traits with n=261. Note that cholesterol and protein intake results are robust to adjustment for total energy intake, whereas other relationships were attenuated by including energy intake as an additional covariate. This suggests that cholesterol and protein intake changes lipid composition (therefore modifying the correlation between participants, which OREML uses to estimate $R^2$) whereas fat intake globally changes lipid levels (which would not affect correlation between participants).*

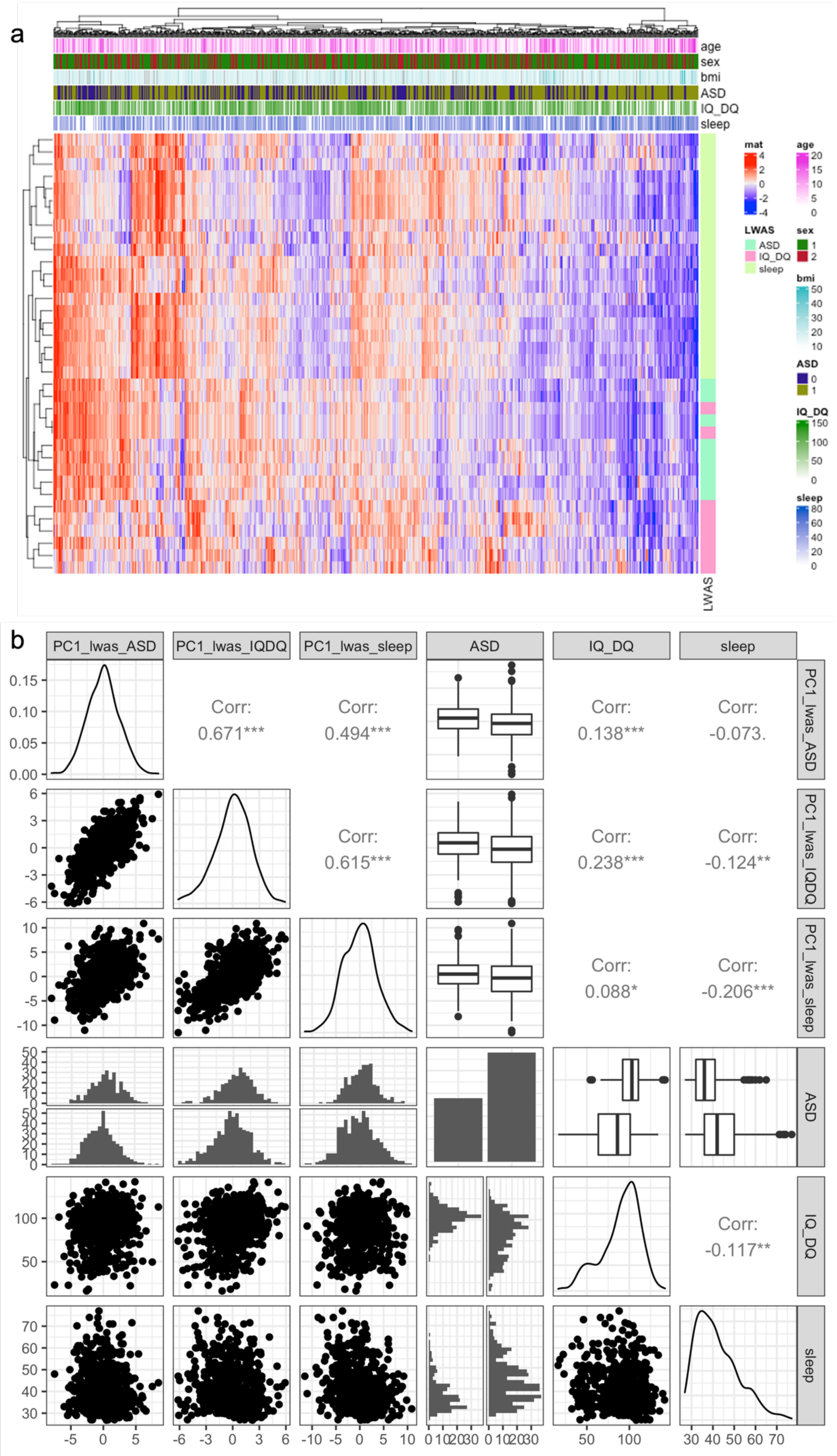


*Supplementary Figure 5: Exploratory data analysis of neurodevelopment-associated lipids. a) Clustered heatmap of neurodevelopment-associated lipids as identified by LWAS and relationship with phenotypes. Sex is coded as 1: male and 2: female. b) Relationships between lipidome profiles for the neurodevelopmental traits of interest (ASD, IQ/DQ and sleep disturbances) and phenotypic measures of ASD, IQ/DQ and sleep disturbances. Box plots provide the median and quartiles of the distribution. Sample size: n=758.*

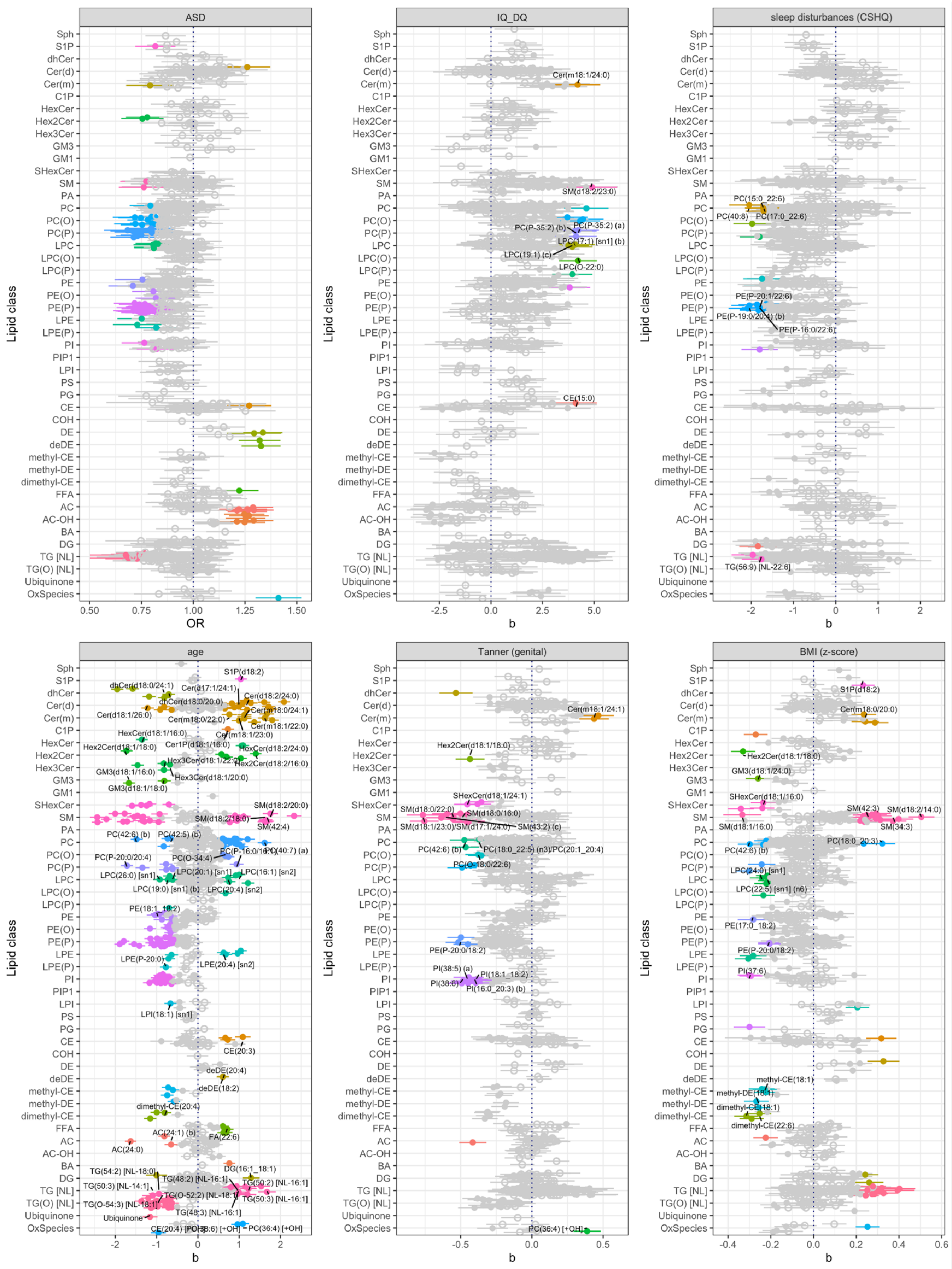


*Supplementary Figure 6: Species-level LWAS (linear model) results including clinical lipids as a fixed effect covariate. Point denotes effect estimate and error bars denote standard error. All analyses included covariates of sex, injection order and storage time; the ASD, IQ/DQ and sleep disturbances LWAS also included age and age² as covariates. The ASD analysis excluded storage time outliers. Open points represent species with association p>0.05, filled points represent species with association p<=0.05. Colour denotes species passing multiple testing correction (dividing by the effective number of independent lipids; see Methods). Text denotes lipid species retained in a backwards stepwise regression model with covariates, representing the effective number of independent LWAS hits. Sample sizes per analysed trait: ASD n=694, IQ/DQ n=642, sleep disturbances n=611; age n=758; Tanner (genital) score n=224; BMI n=715.*

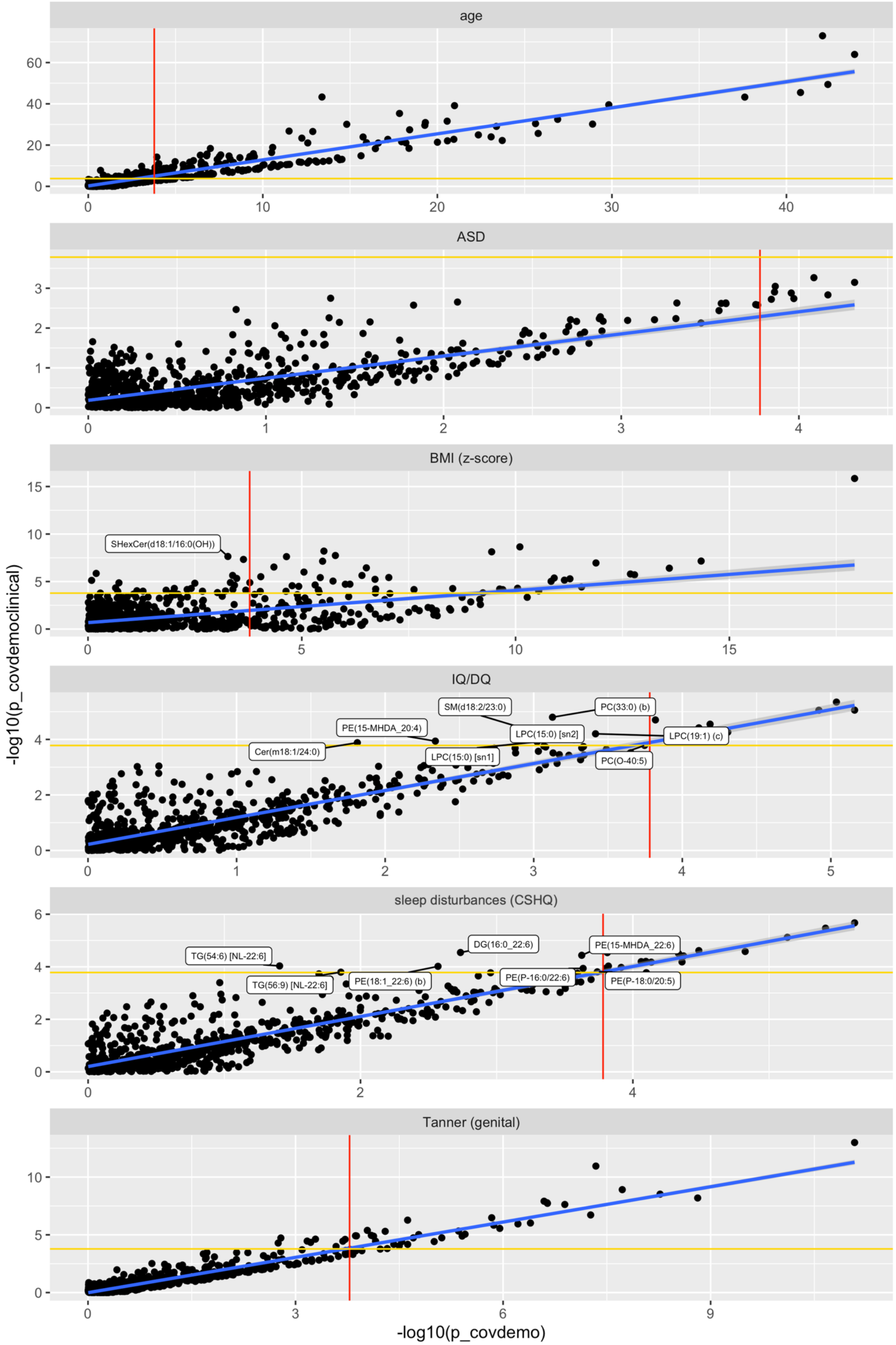


*Supplementary Figure 7: Relationship between species-level LWAS p-values without (Extended Data 1) and with (Supplementary Figure 6) adjustment for clinical lipids. Red and yellow lines denote p-value significance threshold, adjusting for the effective number of lipids.*

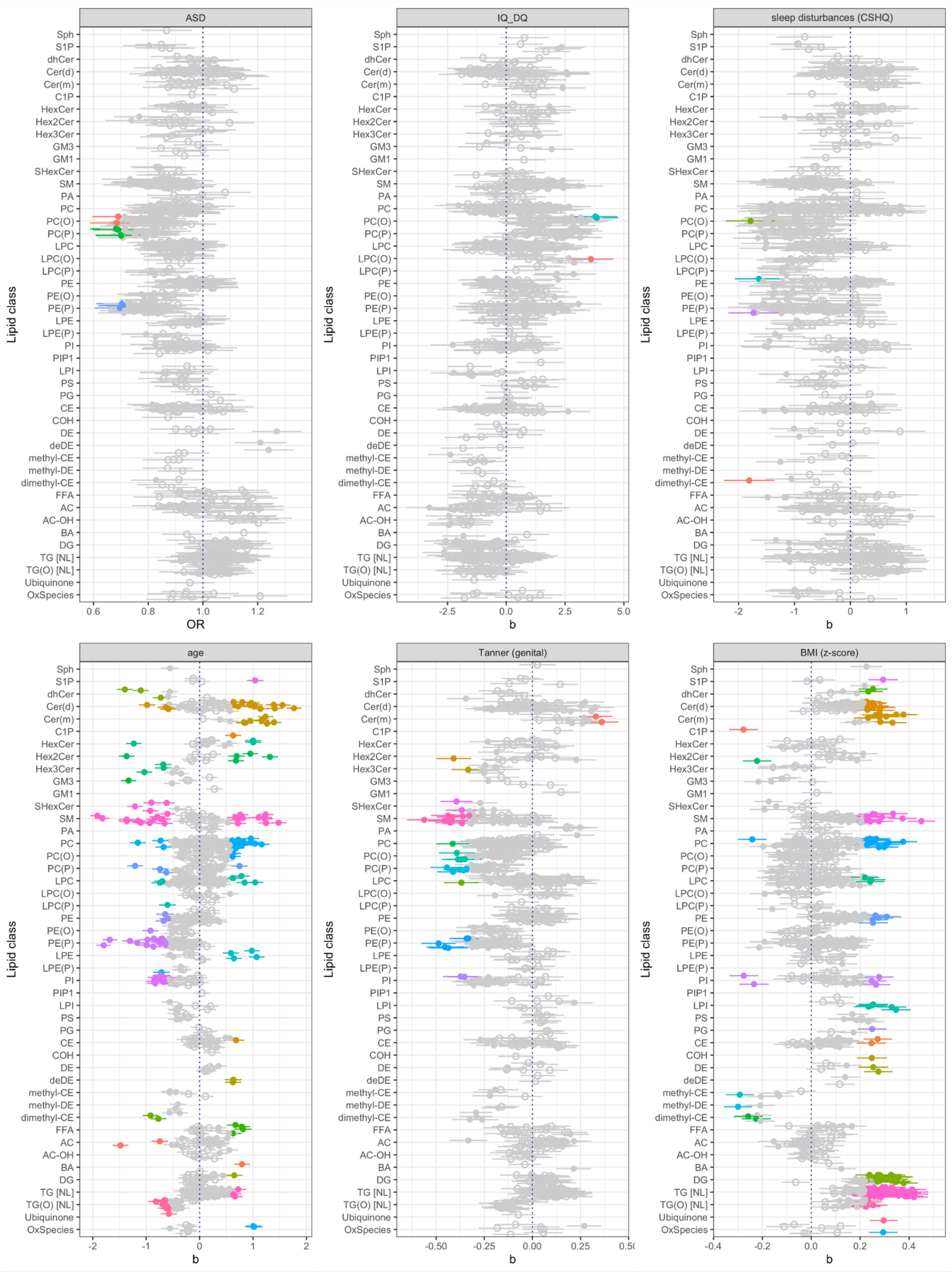


*Supplementary Figure 8: Species-level LWAS (linear model) results including collection time (cosine-transformed) as a covariate in addition to the covariates included in the main analysis in Extended Data 2 (all analyses included the covariates sex, injection order and storage time; the ASD, IQ/DQ and sleep disturbances LWAS also included age and age² as covariates). Point denotes effect estimate and error bars denote standard error. Colour denotes species passing multiple testing correction (dividing by the effective number of independent lipids; see Methods). Sample sizes per analysed trait: ASD n=694, IQ/DQ n=642, sleep disturbances n=611; age n=758; Tanner (genital) score n=224; BMI n=715.*

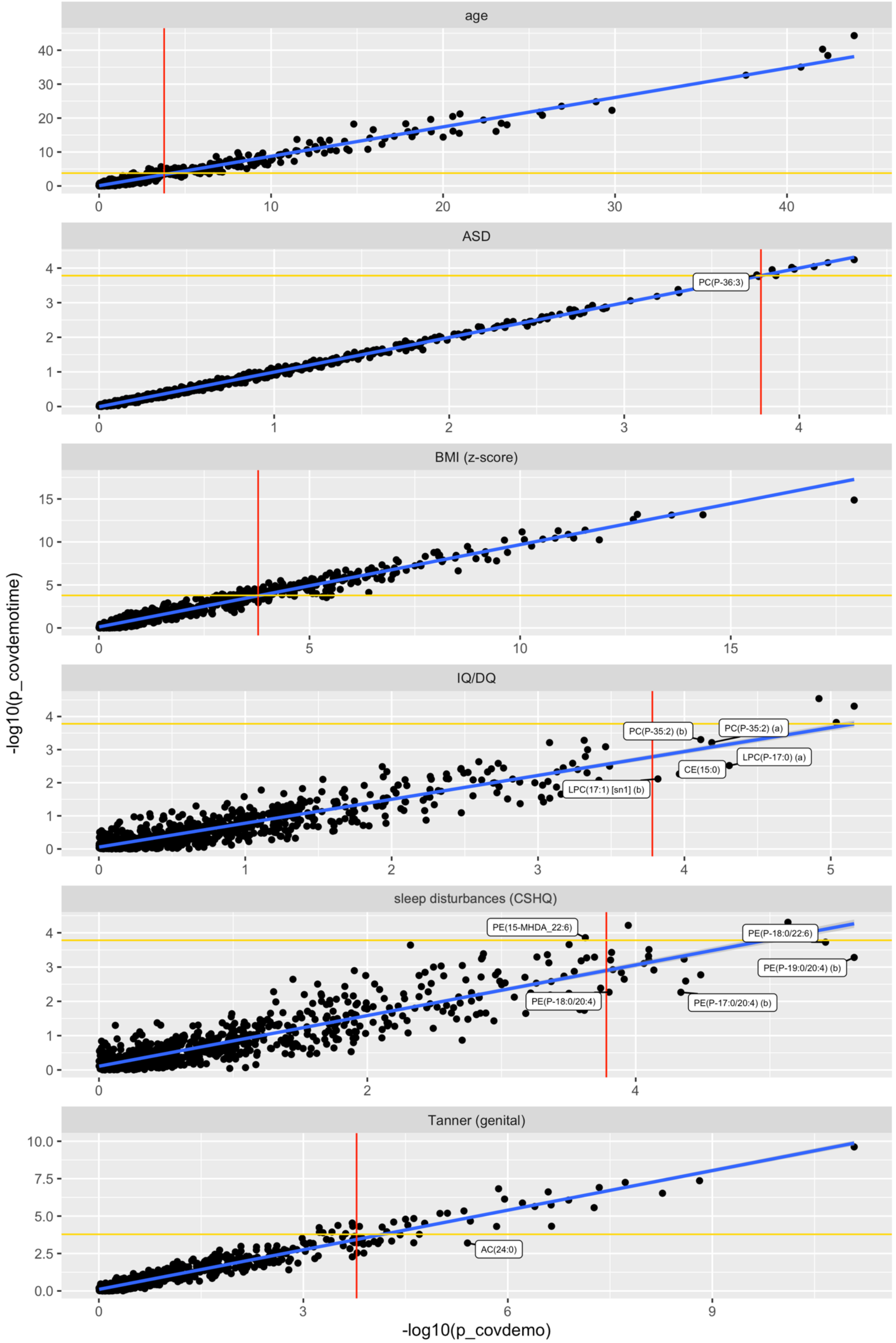


*Supplementary Figure 9: Relationship between species-level LWAS p-values without (Extended Data 2) and with (Supplementary Figure 8) adjustment for cosine-transformed time of day. Red and yellow lines denote p-value significance threshold, adjusting for the effective number of lipids.*

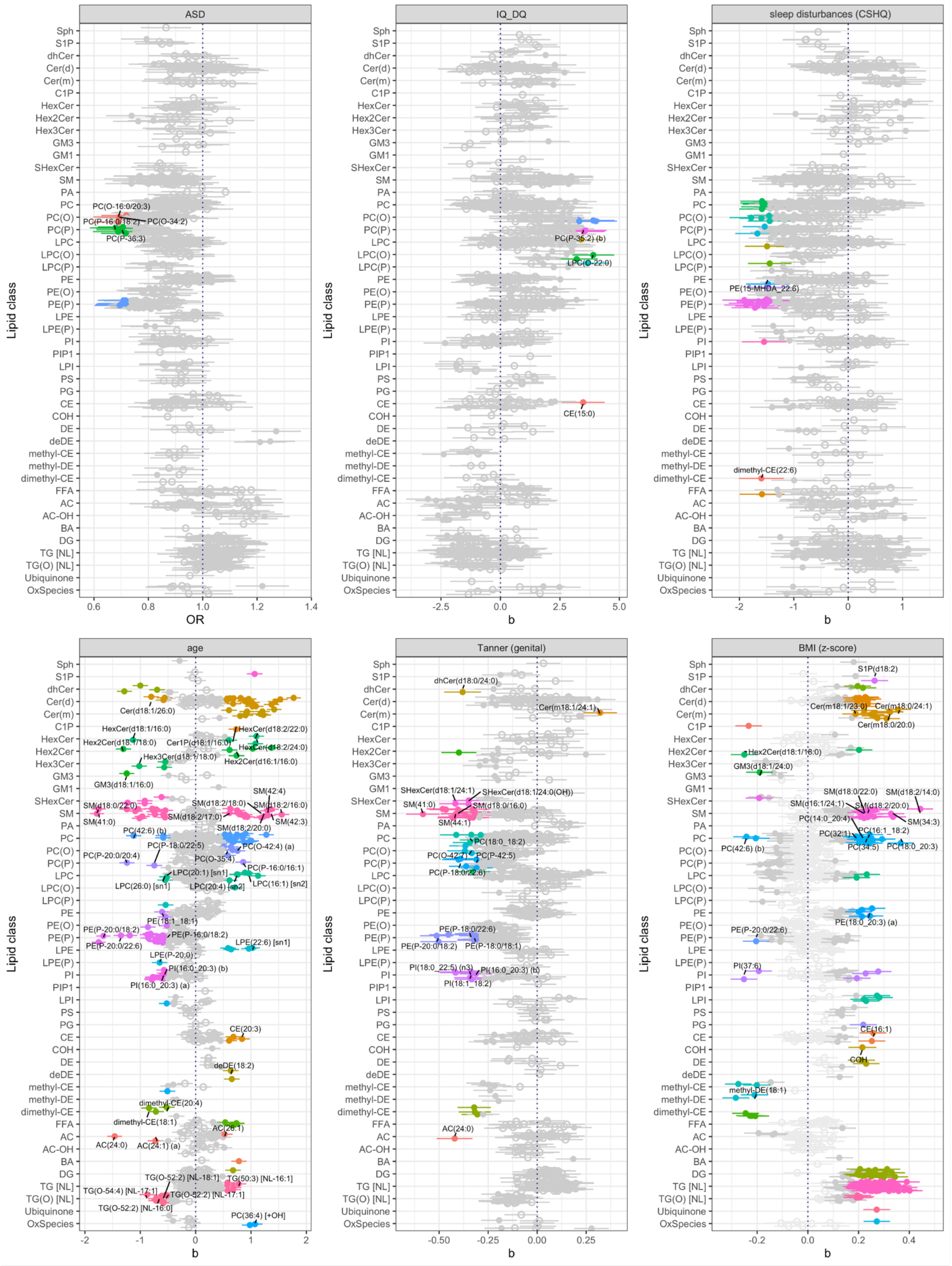

*Supplementary Figure 10: Species-level LWAS (linear model) results lowering the denominator of the multiple testing correction threshold to 95% of the effective number of lipids (see Methods). Point denotes effect estimate and error bars denote standard error. Colour denotes species passing multiple testing correction (dividing by the effective number of independent lipids; see Methods). Sample sizes per trait: ASD n=694, IQ/DQ n=642, sleep disturbances n=611; age n=758; Tanner (genital) score n=224; BMI n=715.*

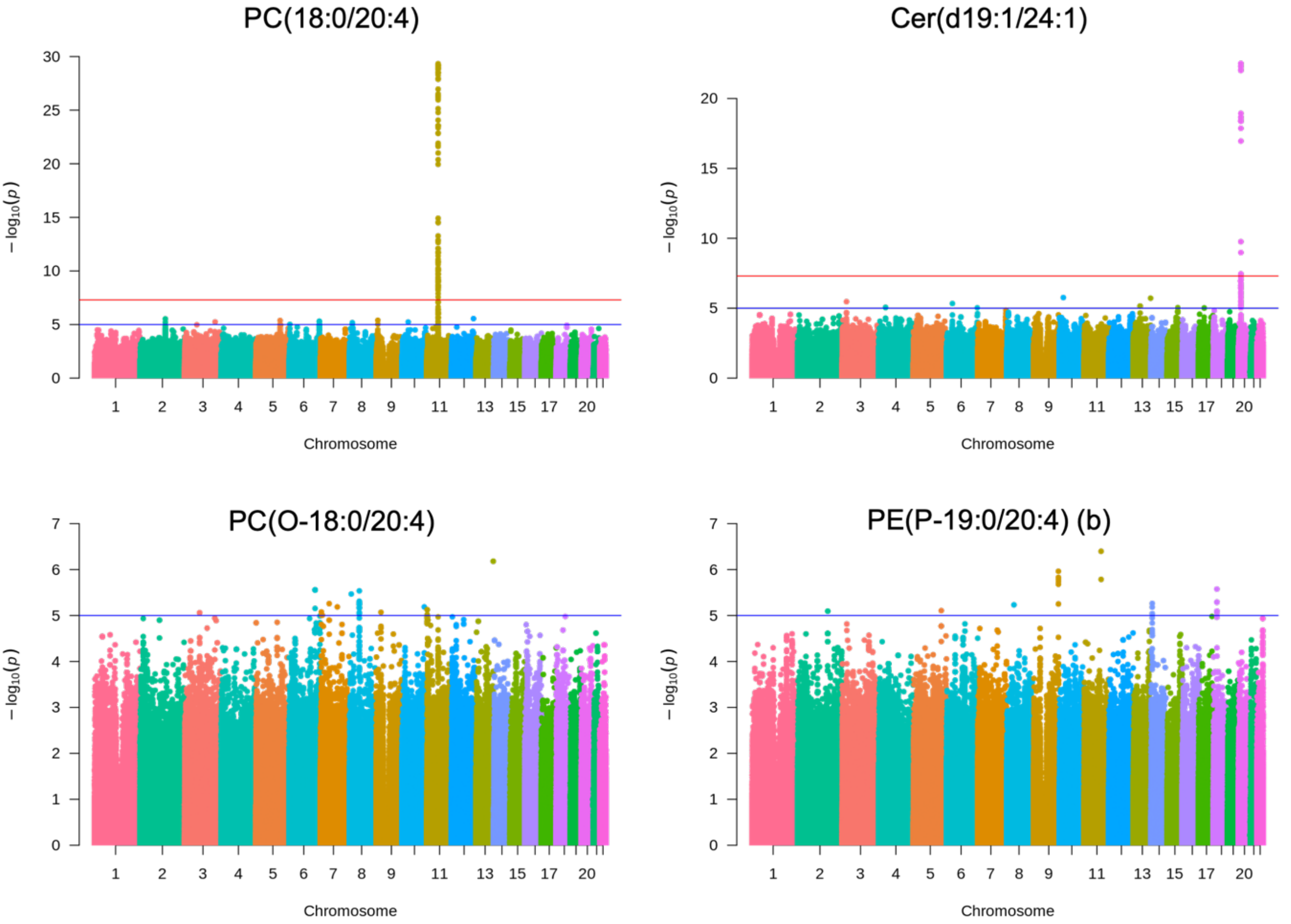


*Supplementary Figure 11: Manhattan plots for GWAS within the AAB+QTAB European subset (n=646) for the following lipids: PC(18:0/20:4), Cer(d19:1/24:1), PC(O-18:0/20:4), PE(P-19:0/20:4) (b). Cer(d19:1/24:1) and PC(18:0/20:4) were chosen for having large proportions of variance explained by the corresponding PGS constructed using the BHS reference and predicted into AAB+QTAB European subset, and the GWAS signal in AAB+QTAB recapitulated the major locus in the BHS GWAS. PC(O-18:0/20:4) and PE(P-19:0/20:4) (b) were chosen as lipids of interest associated with IQ/DQ and sleep problems, respectively. Here, the genetic signal in AAB+QTAB was less clear, which is consistent with the smaller proportions of variance explained by PGS in these lipid traits (Figure 4d). Specifically regarding the FADS gene region SNPs used as instruments in trait-lipid SMR (Figure 4), rs102274 (used for the sleep disturbances/PE(P-19:0/20:4)(b) analysis) was nominally significant (b=-0.23, SE=0.06, p=1.1e-4), whereas rs99780 (used in the IQ/PC(O-18:0/20:4) analysis) was not associated in this paediatric dataset (b=-0.10, SE=0.06, p=7.6e-2).*

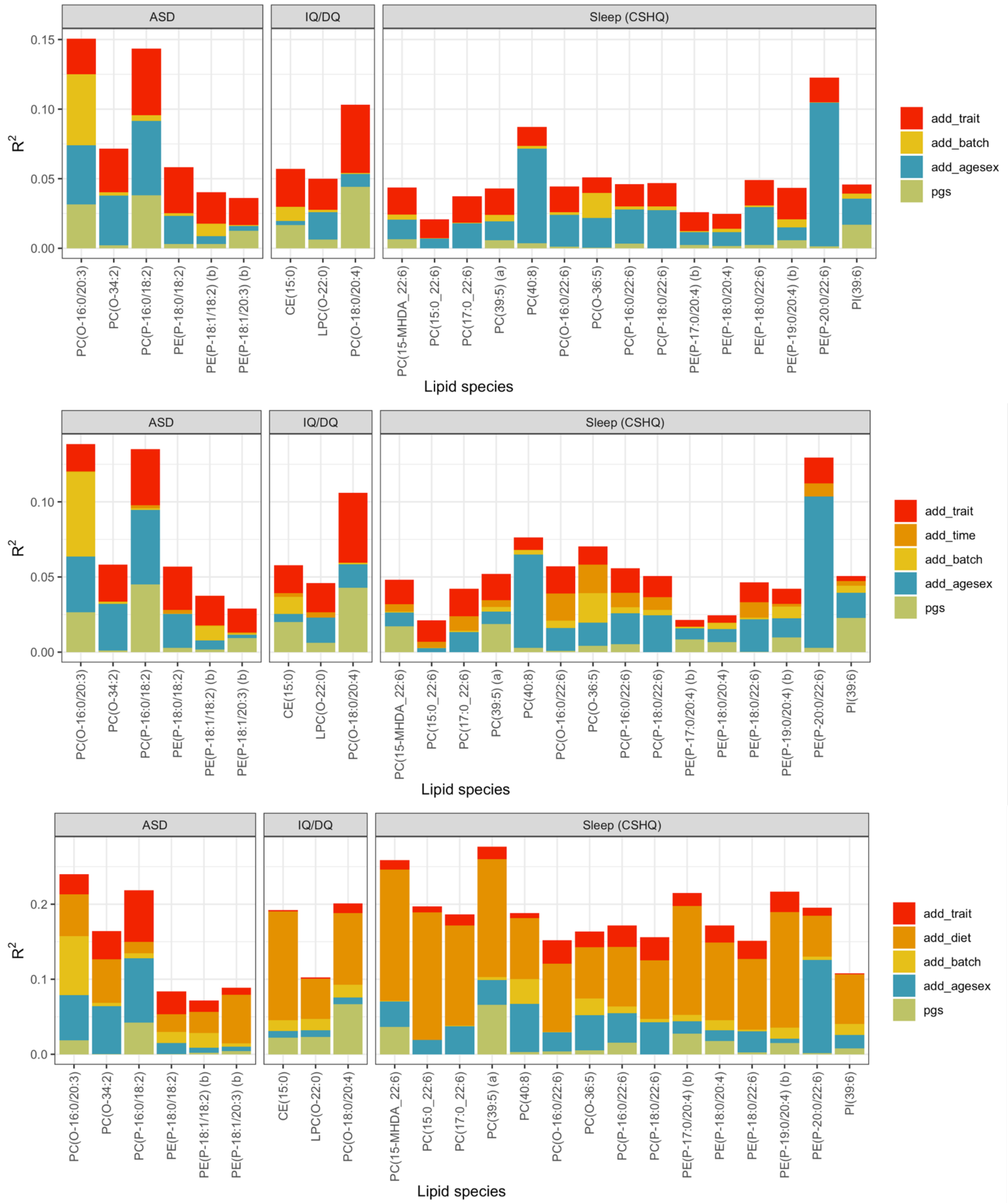


*Supplementary Figure 12: Sensitivity analyses of the variance component analysis for neurodevelopment-associated lipids in Figure 4d. Variance is compartmentalised into contributions from a) PGS + age and sex + batch + neurodevelopmental trait of interest; b) PGS + age and sex + batch + sample collection time of day + neurodevelopmental trait of interest; c) PGS + age and sex + batch + diet + neurodevelopmental trait of interest for lipid species associated with ASD, IQ/DQ and sleep disturbances.*

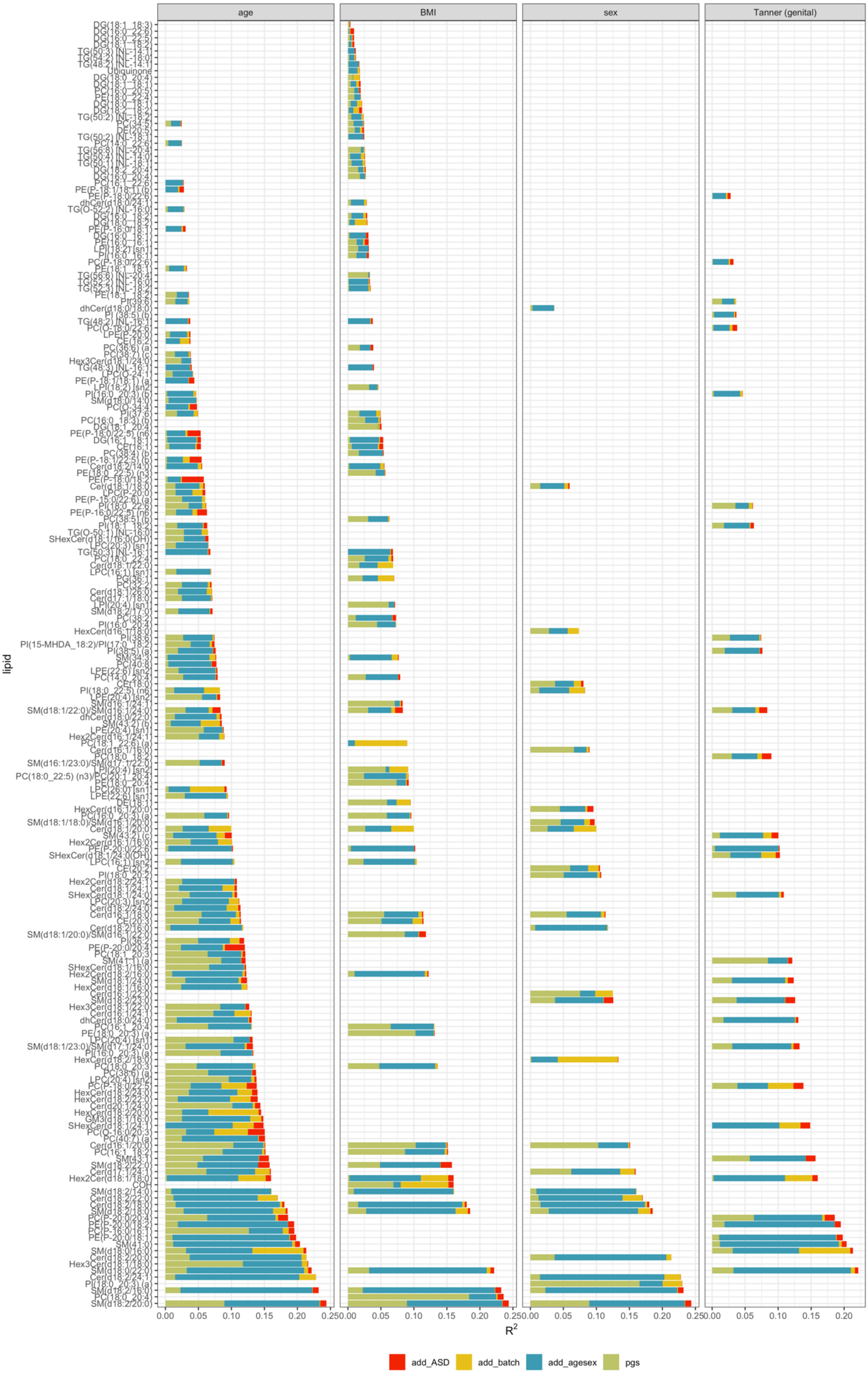


*Supplementary Figure 13: Sensitivity analyses of the variance component analysis for lipids associated with age, BMI z-score, sex and Tanner stage (genital). Variance in lipid concentration is compartmentalised into contributions from PGS + age and sex + batch + ASD diagnosis.*

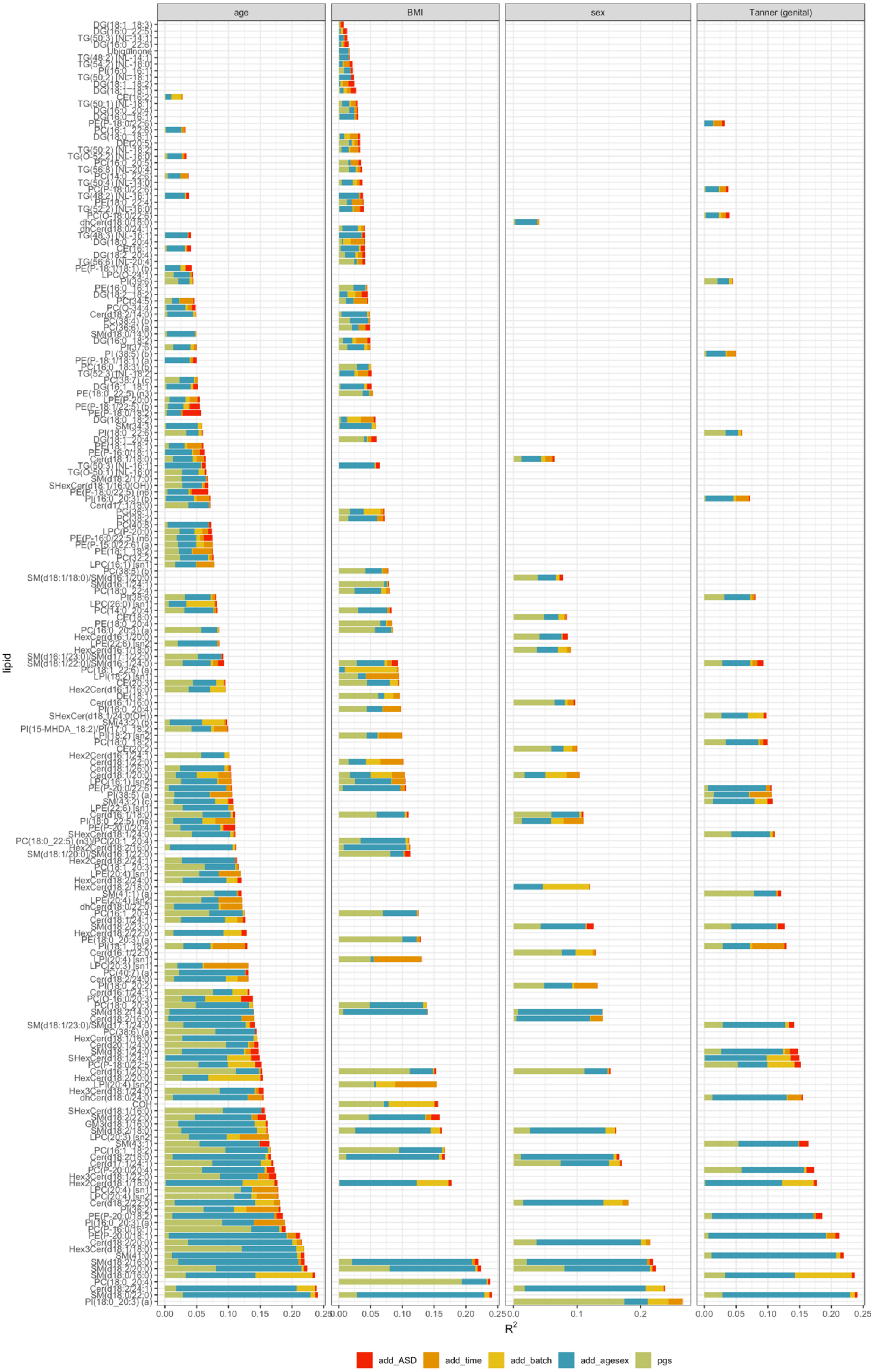


*Supplementary Figure 14: Sensitivity analyses of the variance component analysis for lipids associated with age, BMI z-score, sex and Tanner stage (genital). Variance in lipid concentration is compartmentalised into contributions from PGS + age and sex + batch + sample collection time of day + ASD diagnosis. Note that this analysis only included individuals with complete sample collection time data.*

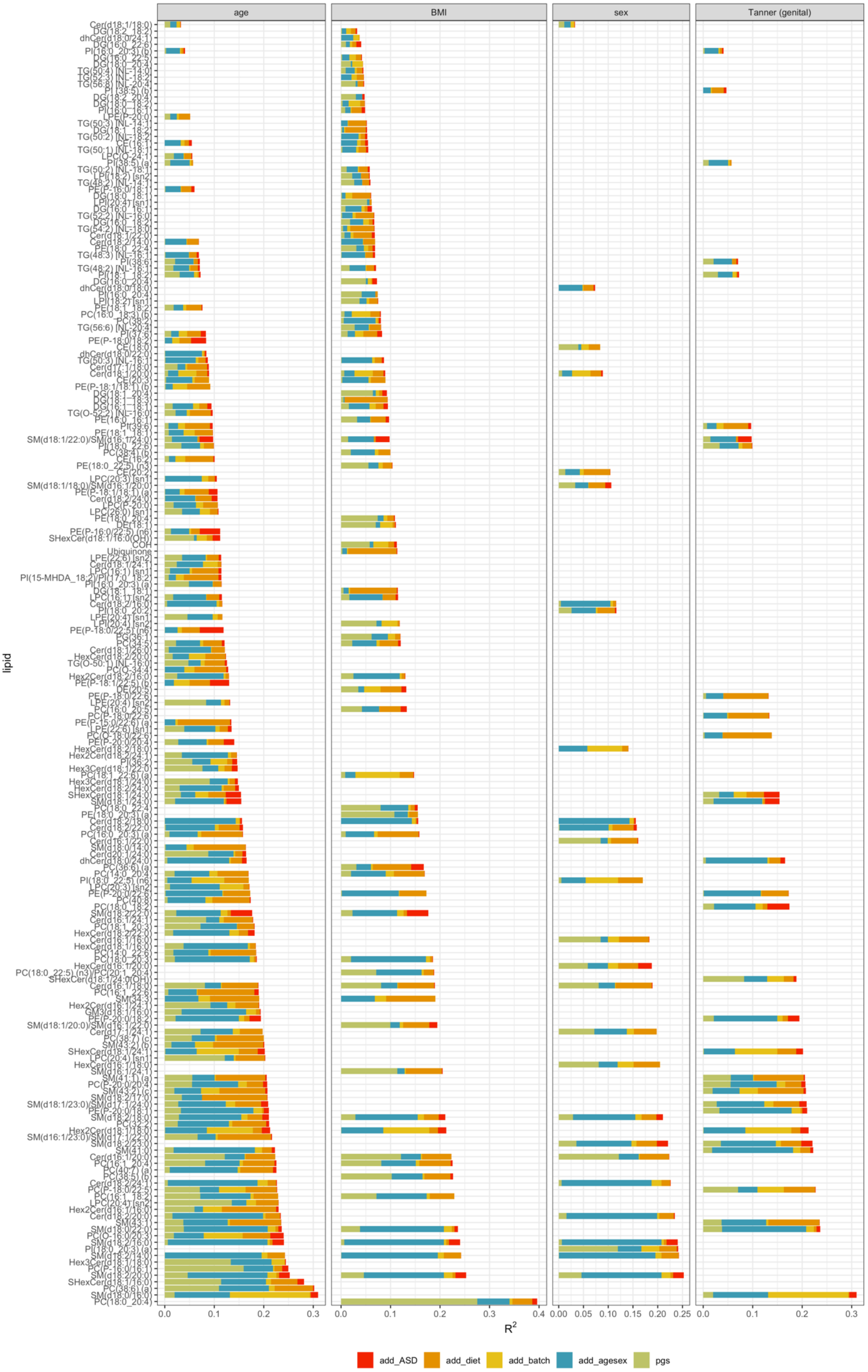


*Supplementary Figure 15: Sensitivity analyses of the variance component analysis for lipids associated with age, BMI z-score, sex and Tanner stage (genital). Variance in lipid concentration is compartmentalised into contributions from PGS + age and sex + batch + dietary PCs 1-3 + ASD diagnosis. Note that this analysis only included individuals with complete dietary data.*

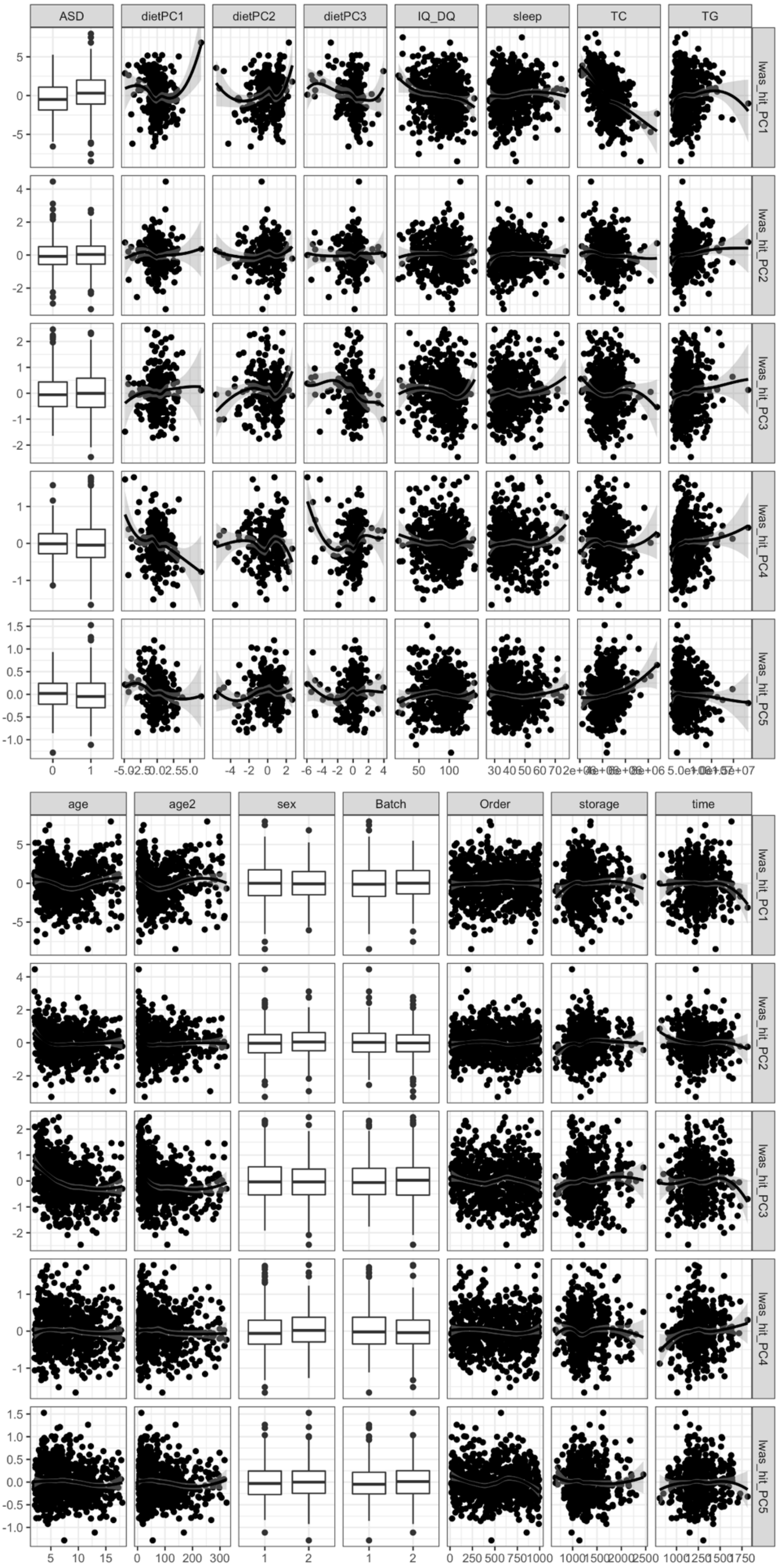


*Supplementary Figure 16: Association between PC1-5 of ASD-associated lipids (PC1 is taken as the "ASD lipidome profile" in the main analysis) and phenotypes including neurodevelopmental phenotypes and covariates used in analyses. "TC" denotes total cholesterol, "TG" denotes triglycerides. Box plots provide the median and quartiles of the distribution. Maximum sample sizes per trait: ASD n=694, IQ/DQ n=642, sleep disturbances n=611; age n=758; Tanner (genital) score n=224; BMI n=715; dietPC1-3 n=261.*

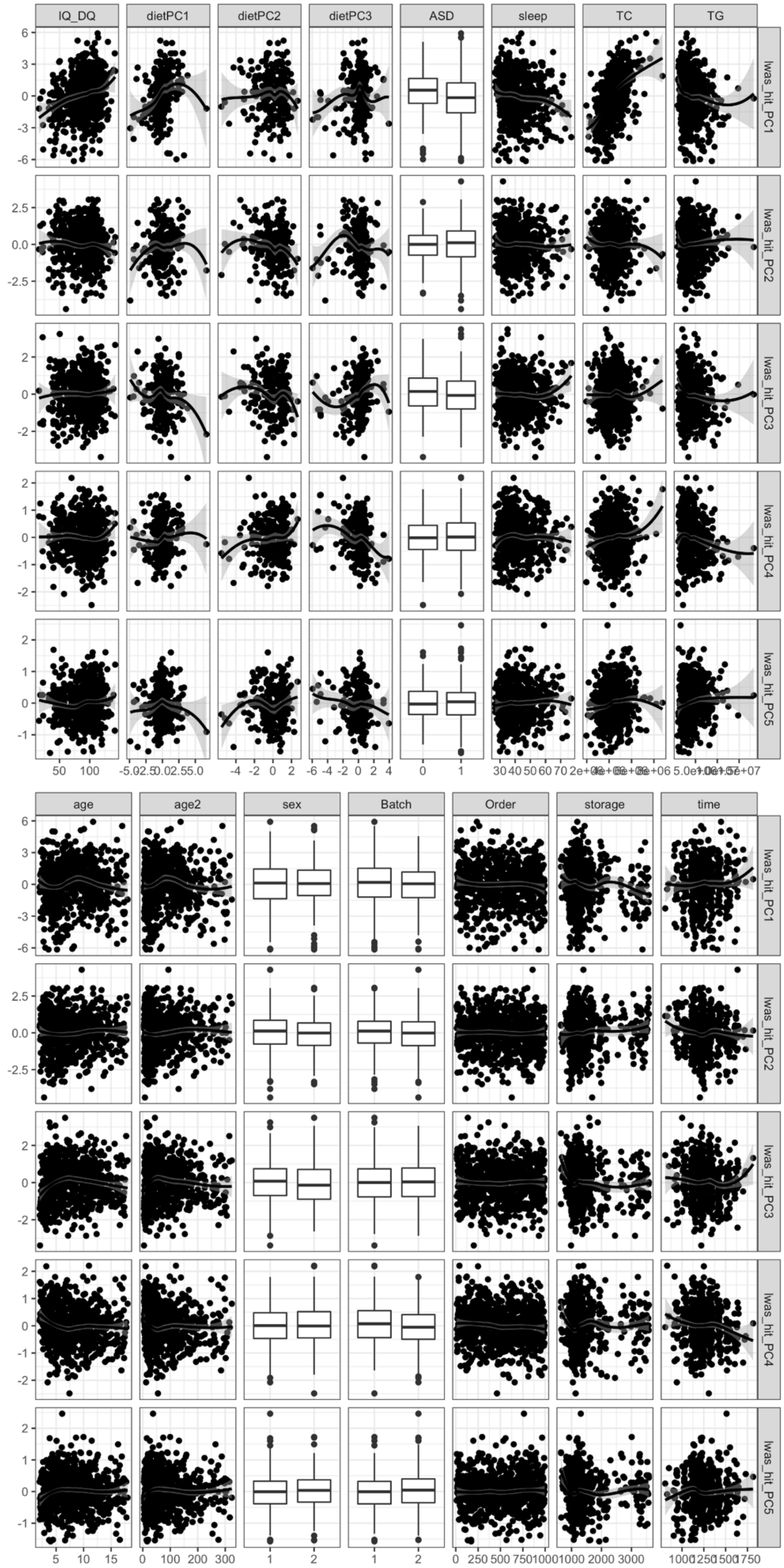


*Supplementary Figure 17: Association between PC1-5 of IQ/DQ-associated lipids (PC1 is taken as the "IQ/DQ lipidome profile" in the main analysis) and phenotypes including neurodevelopmental phenotypes and covariates used in analyses. "TC" denotes total cholesterol, "TG" denotes triglycerides. Box plots provide the median and quartiles of the distribution.Maximum sample sizes per trait: ASD n=694, IQ/DQ n=642, sleep disturbances n=611; age n=758; Tanner (genital) score n=224; BMI n=715; dietPC1-3 n=261.*

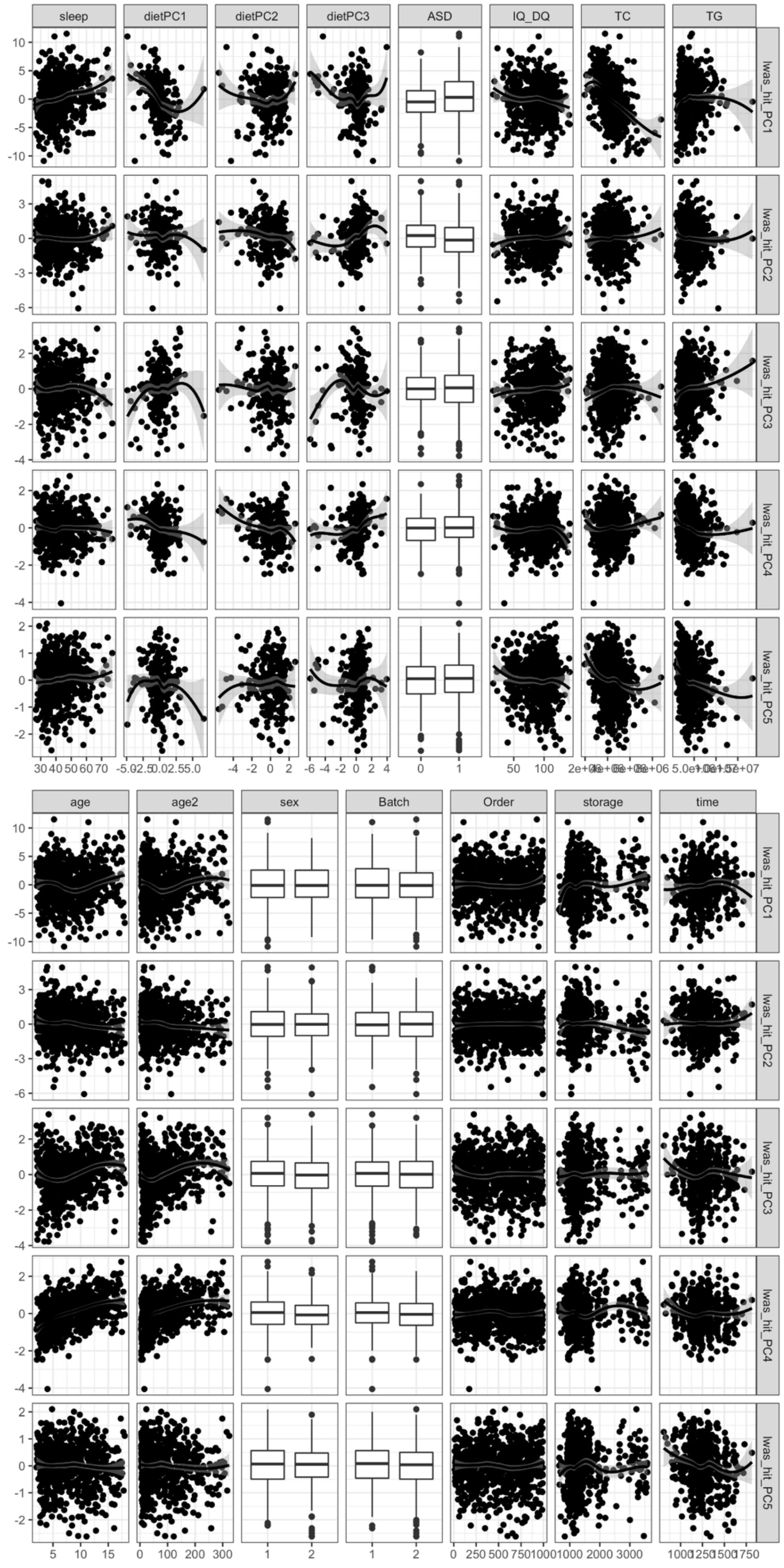


*Supplementary Figure 18: Association between PC1-5 of sleep problem-associated lipids (PC1 is taken as the "sleep disturbances lipidome profile" in the main analysis) and phenotypes including neurodevelopmental phenotypes and covariates used in analyses. "TC" denotes total cholesterol, "TG" denotes triglycerides. Box plots provide the median and quartiles of the distribution. Maximum sample sizes per trait: ASD n=694, IQ/DQ n=642, sleep disturbances n=611; age n=758; Tanner (genital) score n=224; BMI n=715; dietPC1-3 n=261.*

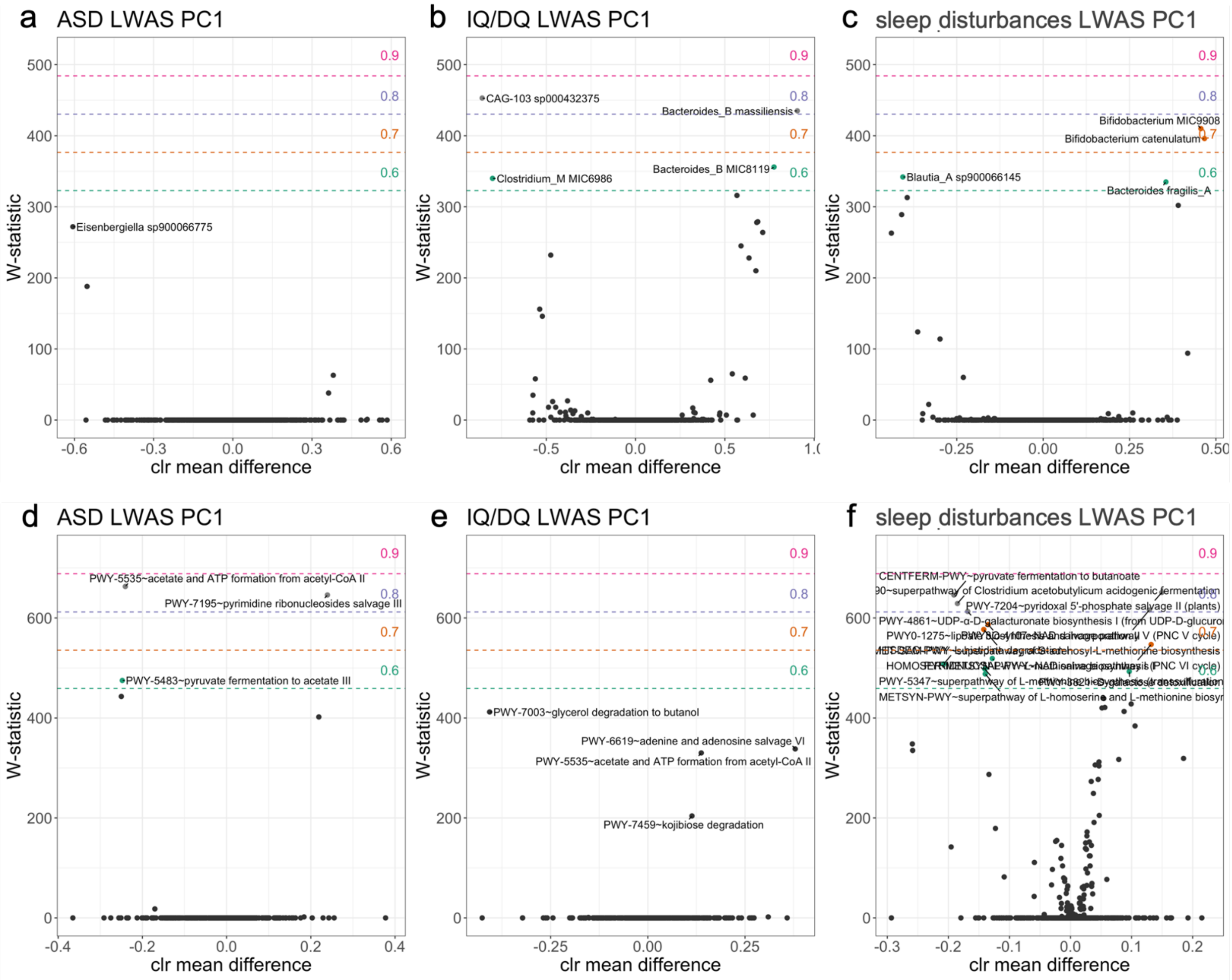


*Supplementary Figure 19: ANCOM sensitivity analysis for stool microbiome species and MetaCyc pathways associated with lipidome profiles for ASD, IQ/DQ and sleep disturbances, including only demographic and batch covariates (no dietary variables) to help examine for overlap with diet-associated microbiome features (Supplementary Figure 20). Sample size n=188.*

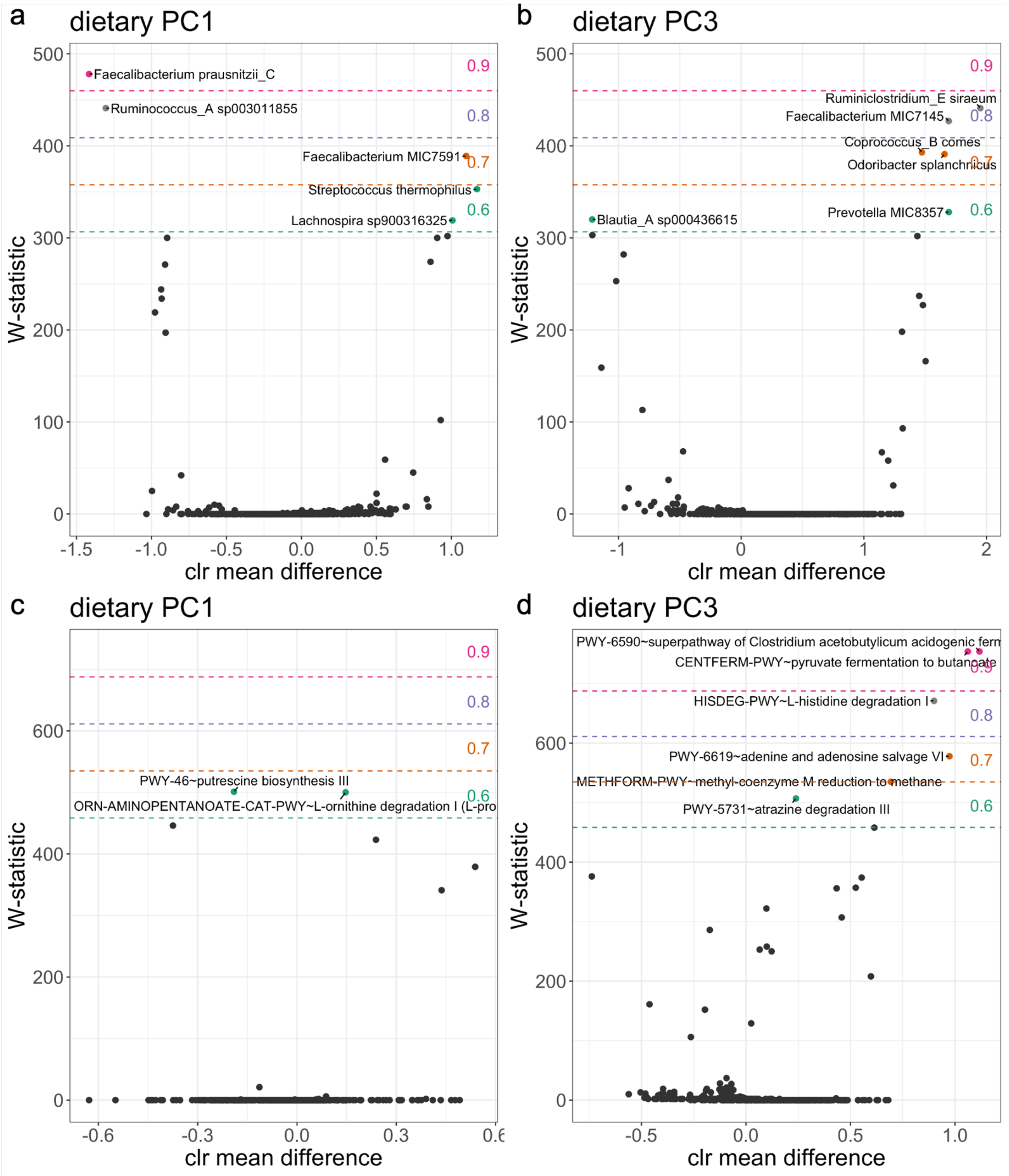


*Supplementary Figure 20: ANCOM analysis for stool microbiome species and MetaCyc pathways associated with the dietary profiles (dietary PC1 and PC3), including demographic and batch covariates. Sample size n=188.*
