# Supplementary material for: Interactions between the lipidome and genetic and environmental factors in autism
Source: Nat Med. 2023 Apr 19;29(4):936–49. doi: 10.1038/s41591-023-02271-1 (PMC10115648; doi:10.1038/s41591-023-02271-1)
Supplement: Supplementary file 2 — Reporting Summary [file 41591_2023_2271_MOESM2_ESM.pdf]

nature portfolio

Corresponding author(s): Chloe Yap, Jacob Gratten

Last updated by author(s): Feb 13, 2023

Reporting Summary

Nature Portfolio wishes to improve the reproducibility of the work that we publish. This form provides structure for consistency and transparency in reporting. For further information on Nature Portfolio policies, see our Editorial Policies and the Editorial Policy Checklist.

Statistics

For all statistical analyses, confirm that the following items are present in the figure legend, table legend, main text, or Methods section.

n/a	Confirmed	
☐	☒	The exact sample size (*n*) for each experimental group/condition, given as a discrete number and unit of measurement
☐	☒	A statement on whether measurements were taken from distinct samples or whether the same sample was measured repeatedly
☐	☒	The statistical test(s) used AND whether they are one- or two-sided<br>*Only common tests should be described solely by name; describe more complex techniques in the Methods section.*
☐	☒	A description of all covariates tested
☐	☒	A description of any assumptions or corrections, such as tests of normality and adjustment for multiple comparisons
☐	☒	A full description of the statistical parameters including central tendency (e.g. means) or other basic estimates (e.g. regression coefficient) AND variation (e.g. standard deviation) or associated estimates of uncertainty (e.g. confidence intervals)
☐	☒	For null hypothesis testing, the test statistic (e.g. *F*, *t*, *r*) with confidence intervals, effect sizes, degrees of freedom and *P* value noted<br>*Give P values as exact values whenever suitable.*
☒	☐	For Bayesian analysis, information on the choice of priors and Markov chain Monte Carlo settings
☒	☐	For hierarchical and complex designs, identification of the appropriate level for tests and full reporting of outcomes
☐	☒	Estimates of effect sizes (e.g. Cohen's *d*, Pearson's *r*), indicating how they were calculated

Our web collection on statistics for biologists contains articles on many of the points above.

Software and code

Policy information about availability of computer code

Data collection	All phenotypic (observational and parent-reported) data was collected on hard copy record forms and entered remotely by each data collection site into databases hosted centrally by Wesley Medical Research using RedCap. Data officers performed a 100% audit on all data entered for accuracy against scanned copies of de-identified record forms.<br><br>Lipidomic profiling was performed using liquid chromatography on electrospray ionisation-tandem mass spectrometry and analysed using MassHunter Quant B08 (Agilent Technologies).
Data analysis	We used custom code in R 4.2.0 for analysis. Code is publicly available at https://github.com/cyap7/ASD_lipidomics_AAB_QTAB.<br>We used the following other software packages: OSCAv0.46, GCTAv1.93.2beta, PLINKv1.90, SMRv1.03.

For manuscripts utilizing custom algorithms or software that are central to the research but not yet described in published literature, software must be made available to editors and reviewers. We strongly encourage code deposition in a community repository (e.g. GitHub). See the Nature Portfolio guidelines for submitting code & software for further information.

Data

Policy information about availability of data

All manuscripts must include a data availability statement. This statement should provide the following information, where applicable:

- Accession codes, unique identifiers, or web links for publicly available datasets
- A description of any restrictions on data availability
- For clinical datasets or third party data, please ensure that the statement adheres to our policy

The AAB datasets (lipidomics, SNP genotyping, stool metagenomics, phenotype data) supporting the conclusions of this article are available by application to the Australian Autism Biobank within the Cooperative Research Centre for Living with Autism (Autism CRC): https://www.autismcrc.com.au/biobank. These data are not publicly available for ethical reasons, and applications are reviewed by a board including autistic people.
The QTAB dataset used in these analyses is available with mediated access through the UQ eSpace repository at https://doi.org/10.48610/dc9bf34.
The ABCD dataset is available by application through the NIMH Data Archive: http://dx.doi.org/10.15154/1523041.
Busselton Health Study lipidomics GWAS results are available at https://metabolomics.baker.edu.au/

Human research participants

Policy information about studies involving human research participants and Sex and Gender in Research.

Reporting on sex and gender	We made reference only to sex (biological attribute) in this article, which was determined by self-reporting and confirmed with genetic sex. We included a total of 500 males and 265 females; this male preponderance is expected in autism, which is more common in males. We have provided the sex breakdown per group (ASD/SIB/UNR) in Supplementary Table 1. We have included analyses taking sex as the dependent variable.<br><br>In our analysis, we have included sex as a covariate in all analyses (except where explicitly stated and these would be as sensitivity analyses). We have also directly investigated associations between sex and the lipidome (Figure 2b, Supplementary Table 10), and specifically assessed for associations between sex and neurodevelopmental traits in (what is currently) Supplementary Note 2.<br><br>We did not perform sex-stratified analyses as this would result in an underpowered analysis while also increasing the multiple testing burden.
Population characteristics	A detailed breakdown of demographics per participant group is provided in Supplementary Table 1. We considered three participant groups: those with ASD diagnosis (n=485), undiagnosed siblings (SIB; n=160) and undiagnosed, unrelated children (UNR; n=120) The mean age was 8.0 (SD=3.9), and this was approximately similar across the ASD, SIB and UNR groups. These children were similar with respect to BMI. Children in the ASD group on average had lower IQ (mean 92, SD=24) than the SIB (mean=102, SD=14) and UNR (mean=102, SD=15) groups, which is to be expected given the association of intellectual disability with ASD. Children in the ASD group had higher rates of sleep problems (mean=44, SD=10) than the SIB (mean=38, SD=8) and UNR (mean=37, SD=7), which is also to be expected as autistic children are more likely to have sleep problems.
Recruitment	The AAB participants include children with an ASD diagnosis (recruited from autism clinics and research centres across Australia's four largest cities: Sydney, Melbourne, Brisbane, Perth; no exclusion criteria), and their siblings ("SIB") without a diagnosis (Alvares et al., 2018). The group of unrelated children ("UNR") without a diagnosis were recruited by the AAB (recruited from the community; exclusion criteria: having an ASD diagnosis) and QTAB (typically developing children recruited from the community). The requirement to provide biological samples may have selected for children who were better able to tolerate this (eg. those with fewer behavioural challenges), which may limit the generalisability of the results. Investigators attempted to include this subset of individuals by timing biological sample collection with other procedures, but this was not always possible.
Ethics oversight	• NSW: Sydney Children's Hospital Network HREC, approval number HREC/14/SCHN/269.<br>• QLD: Mater Health Services HREC, approval number HREC/14/MHS/212; the University of Queensland, approval number 2014001079; QTAB Project: Children's Health Queensland HREC, approval number HREC/16/QRCH/270; The University of Queensland, approval number 2016001784/ HREC/16/QRCH/270<br>• VIC: La Trobe University, approval number HEC16/104<br>• WA: Princess Margaret Hospital for Children approval number 2014029EP; the University of Western Australia approval number RA/4/1/8184

Note that full information on the approval of the study protocol must also be provided in the manuscript.

Field-specific reporting

Please select the one below that is the best fit for your research. If you are not sure, read the appropriate sections before making your selection.

☒ Life sciences ☐ Behavioural & social sciences ☐ Ecological, evolutionary & environmental sciences

For a reference copy of the document with all sections, see nature.com/documents/nr-reporting-summary-flat.pdf

Life sciences study design

All studies must disclose on these points even when the disclosure is negative.

Sample size	We chose sample size to maximise the number of individuals with complete phenotyping and multi-omics data, within the allocated budget. To our knowledge, this is the largest study of the lipidome in ASD to date.
Data exclusions	We have included a section on outliers and data exclusions in Supplementary Note 2, copied and pasted here for convenience:<br><br>The n=7 “statistical outliers” (see Methods) were excluded for all analyses except for those within the “Lipidome outliers” section in which they were specifically interrogated for potential biological explanations. The rationale for this is that including these outliers when investigating mean group differences (i.e., OREML, LWAS, PGS, trait lipidome associations) could bias the results.<br><br>For the analyses investigating mean group differences related to ASD diagnosis, we excluded n=64 “storage duration outliers”, defined as samples with storage duration >=2500 days. This was motivated by our observation that the ASD OREML analysis without covariates (“nocov”, Figure 2b) had significantly higher R2 than the analysis including demographic and batch variables (“covdemo”). Closer inspection of the data revealed that storage time was confounded with ASD diagnostic status (Supplementary Figure 2), which is because the AAB initially recruited only children with an ASD diagnosis (who typically have co-occurring intellectual disability and sleep problems), before expanding to include undiagnosed children. Furthermore, we found that lipid species and classes that are known to correlate with sample degradation (the ratio of LPC(O-18:0) and PC(O-18:0/20:4), and the oxidised species class) were associated with ASD diagnostic status. To ameliorate this confounder, we excluded individuals with sample storage >2500 days in the ASD analyses. However, other phenotypes did not suffer the same imbalance in sample storage time (Supplementary Figure 2), and we confirmed in sensitivity analyses that excluding these individuals did not have a significant affect our conclusions; hence, there was no need to exclude storage time outliers in these other analyses. We also note that individuals whom contributed dietary variables, had had shorter storage time, so including dietary data as covariates effectively excluded the storage time outliers.<br><br>To additionally reduce the effect of outliers on our results, we performed a rank-based inverse normal transform of the lipidomics data in the OREML, LWAS, and trait lipidome analyses.
Replication	In the absence of an equivalent dataset, we performed "genetic replication". We first used Summary Mendelian Randomisation to determine potential causality of lipid-trait relationship, using publicly-available GWAS summary statistics of similar traits. This analysis confirmed our initial results. Secondly, we imputed lipid levels in another paediatric dataset with SNP genotyping data (ABCD), again leveraging GWAS summary statistics for various lipid species. This analysis was more equivocal, which was attributed to statistical power (of the GWAS, target dataset, and traits of interest).
Randomization	Randomisation was performed for all stages of sample processing and data acquisition to avoid potential bias
Blinding	Investigators were blinded for biological sample processing and data acquisition. There were no interventions in this study to blind to.

Reporting for specific materials, systems and methods

We require information from authors about some types of materials, experimental systems and methods used in many studies. Here, indicate whether each material, system or method listed is relevant to your study. If you are not sure if a list item applies to your research, read the appropriate section before selecting a response.

Materials & experimental systems

n/a	Involved in the study
☒	☐ Antibodies
☒	☐ Eukaryotic cell lines
☒	☐ Palaeontology and archaeology
☒	☐ Animals and other organisms
☒	☐ Clinical data
☒	☐ Dual use research of concern

Methods

n/a	Involved in the study
☒	☐ ChIP-seq
☒	☐ Flow cytometry
☒	☐ MRI-based neuroimaging

March 2021
